# Supplementary material for: Hospital management of major stroke types in Chinese adults: a population-based study of 20 000 hospitalised stroke cases
Source: BMJ Open. 2021 Nov 16;11(11):e054265. doi: 10.1136/bmjopen-2021-054265 (PMC8596044; doi:10.1136/bmjopen-2021-054265)
Supplement: Author's manuscript [file bmjopen-2021-054265.draft_revisions.pdf]

BMJ Open is committed to open peer review. As part of this commitment we make the peer review history of every article we publish publicly available.

When an article is published we post the peer reviewers' comments and the authors' responses online. We also post the versions of the paper that were used during peer review. These are the versions that the peer review comments apply to.

The versions of the paper that follow are the versions that were submitted during the peer review process. They are not the versions of record or the final published versions. They should not be cited or distributed as the published version of this manuscript.

BMJ Open is an open access journal and the full, final, typeset and author-corrected version of record of the manuscript is available on our site with no access controls, subscription charges or pay-per-view fees (http://bmjopen.bmj.com).

If you have any questions on BMJ Open's open peer review process please email info.bmjopen@bmj.com

Hospital management of major stroke types in Chinese adults: a population-based study of 20,000 hospitalised stroke cases

Journal:	*BMJ Open*
Manuscript ID	bmjopen-2021-054265
Article Type:	Original research
Date Submitted by the Author:	09-Jun-2021
Complete List of Authors:	Qin, Haiqianq; China National Clinical Research Centre for Neurological Diseases, Tiantian Hospital Beijing<br>Turnbull, Iain; University of Oxford, Clinical Trial Service Unit and Epidemiological Studies, NDPH<br>Chen, Yiping; University of Oxford, Clinical Trial Service Unit and Epidemiological Studies, NDPH<br>Wright, Neil ; University of Oxford, Clinical Trial Service Unit and Epidemiological Studies, NDPH<br>Liu, Liping; China National Clinical Research Centre for Neurological Diseases, Tiantian Hospital Beijing, Department of Neurology<br>Pei, Pei; Chinese Academy of Medical Sciences and Peking Union Medical College<br>Tang, Wei; Pengzhou Traditional Chinese Medical Hospital<br>Xiang, Shengping; Pengzhou Traditional Chinese Medical Hospital<br>Guo, Yu; Chinese Academy of Medical Sciences and Peking Union Medical College<br>Zhao, Xingquan; China National Clinical Research Centre for Neurological Diseases, Tiantian Hospital Beijing<br>Clarke, Robert; University of Oxford,<br>Li, Liming; Chinese Academy of Medical Sciences and Peking Union Medical College<br>Wang, Yongjun; China National Clinical Research Centre for Neurological Diseases, Tiantian Hospital Beijing<br>Chen, Zhengming; University of Oxford, Clinical Trial Service Unit and Epidemiological Studies, NDPH
Keywords:	Neurology < INTERNAL MEDICINE, EPIDEMIOLOGY, Health policy < HEALTH SERVICES ADMINISTRATION & MANAGEMENT

SCHOLARONE™ Manuscripts

I, the Submitting Author has the right to grant and does grant on behalf of all authors of the Work (as defined in the below author licence), an exclusive licence and/or a non-exclusive licence for contributions from authors who are: i) UK Crown employees; ii) where BMJ has agreed a CC-BY licence shall apply, and/or iii) in accordance with the terms applicable for US Federal Government officers or employees acting as part of their official duties; on a worldwide, perpetual, irrevocable, royalty-free basis to BMJ Publishing Group Ltd ("BMJ") its licensees and where the relevant Journal is co-owned by BMJ to the co-owners of the Journal, to publish the Work in this journal and any other BMJ products and to exploit all rights, as set out in our licence.

The Submitting Author accepts and understands that any supply made under these terms is made by BMJ to the Submitting Author unless you are acting as an employee on behalf of your employer or a postgraduate student of an affiliated institution which is paying any applicable article publishing charge ("APC") for Open Access articles. Where the Submitting Author wishes to make the Work available on an Open Access basis (and intends to pay the relevant APC), the terms of reuse of such Open Access shall be governed by a Creative Commons licence – details of these licences and which Creative Commons licence will apply to this Work are set out in our licence referred to above.

Other than as permitted in any relevant BMJ Author's Self Archiving Policies, I confirm this Work has not been accepted for publication elsewhere, is not being considered for publication elsewhere and does not duplicate material already published. I confirm all authors consent to publication of this Work and authorise the granting of this licence.

For peer review only

Hospital management of major stroke types in Chinese adults: a population-based study of 20,000 hospitalised stroke cases

Haiqiang Qin MD*[1], Iain Turnbull MRCP* [2], Yiping Chen DPhil* [2,3], Neil Wright PhD[2], Liping Liu MD[1], Pei Pei BSc[4], Wei Tang BSc[6], Shengping Xiang BSc[7], Yu Guo MSc[4], Xingquan Zhao MD[1], Robert Clarke FRCP[2], Liming Li MPH[4,5], Yongjun Wang MD[1], Zhengming Chen DPhil[2] on behalf of the China Kadoorie Biobank Collaborative Group

Equal contribution

1. China National Clinical Research Centre for Neurological Diseases; Department of Neurology, Beijing Tiantan Hospital, Capital Medical University, Beijing, China
2. Clinical Trial Service Unit and Epidemiological Studies Unit (CTSU), Nuffield Department of Population Health, University of Oxford, Oxford, UK
3. Medical Research Council Population Health Research Unit (PHRU), Nuffield Department of Population Health, University of Oxford, Oxford, UK
4. Chinese Academy of Medical Sciences, Dong Cheng District, Beijing, China
5. Department of Epidemiology and Biostatistics, School of Public Health, Peking University Health Science Centre, Beijing, China
6. Emergency Department, Pengzhou Traditional Chinese Medical Hospital, Pengzhou, Sichuan, China
7. Pengzhou Tongyi Hospital, Pengzhou, Sichuan, China

Address for correspondence

Prof Robert Clarke
Clinical Trial Service Unit
NDPH
Big Data Institute Building
Old Road Campus
University of Oxford
E-mail: Robert.clarke@ndph.ox.ac.uk

Dr Yiping Chen
NDPH
Big Data Institute Building
Old Road Campus
University of Oxford
E-mail: yiping.chen@ndph.ox.ac.uk

Word count: Abstract n=295; text n=2,916
3 Tables, 2 Figure 1 eTable and 4 eFigures

CKB Research Tracker no. 2018-0071

Key words: Ischemic Stroke, hospital treatment, antiplatelet medication, blood pressure-lowering medication, lipid-lowering medication, traditional Chinese medicines

2 June 2021

For peer review only - http://bmjopen.bmj.com/site/about/guidelines.xhtml

Abstract

Background: Little is known about use of hospital treatments for stroke types in Chinese adults. We assessed the hospital management of stroke by pathological type, sex, age, calendar year, hospital tier, region and other factors.

Methods: The China Kadoorie Biobank recruited >0.5 million adults during 2004-08 from ten diverse areas and medical records were retrieved from 20,229 participants (n=261 hospitals) hospitalised with a first-ever incident stroke over an 11-year follow-up period.

Results: Among 20,299 first-ever stroke cases, 17,306 (85%) had ischemic stroke (IS; 7,123 non-lacunar [non-LACI], 6,690 lacunar [LACI], 3,493 had silent cerebral infarction [SCI]), 2,623 (13%) had intracerebral hemorrhage (ICH), and 370 (2%) had subarachnoid hemorrhage (SAH). Among IS cases, antiplatelet treatment was used by 64% (65% non-LACI, 66% LACI, 56% SCI), lipid-lowering by 50% (52% non-LACI, 53% LACI, 43% SCI), and blood pressure-lowering by ~42% of all IS types, with positive trends in use of these treatments by calendar year and hospital tier. Among ICH cases, 53% used blood pressure-lowering and 10% used lipid-lowering treatments, respectively. In contrast, traditional Chinese medicines (TCM) were used by 59% of IS (50% non-LACI, 62% LACI, 74% SCI), 38% of ICH, and 30% of SAH cases, with positive trends by calendar year and by hospital tier.

Conclusions: Among IS cases, use of antiplatelet and lipid-lowering medications and TCM increased in recent years and exceeded use of blood pressure-lowering treatment. In contrast, blood pressure-lowering treatment was most widely used for ICH, but still only half of all ICH cases had such treatment.

For peer review only - http://bmjopen.bmj.com/site/about/guidelines.xhtml

Strengths and Limitations

Strengths

- In a 12-year follow-up of 0.5 Million Chinese adults recruited into the China Kadoorie Biobank in 2004-2008, medical records were retrieved on 20,299 first stroke cases. All stroke cases were classified by pathological types by local neurologists and details of in-hospital management were recorded by stroke types.

- Among 17,306 (85%) hospitalised cases with first ischemic stroke, anti-platelet treatment was used by 64%, lipid-lowering by 50% and blood pressure-lowering by 42% with positive trends by calendar year and hospital tier.

- Among 2,623 (14%) with intra-cerebral haemorrhage, 53% used blood pressure-lowering treatment and 10% used lipid-lowering treatment.

- Traditional Chinese Medicines were used 59% of ischemic stroke cases and 38% of intra-cerebral haemorrhage stroke cases.

Limitations

- However, the study was unable to ascertain the extent to which these stroke cases continued their medication after discharge from hospital.

For peer review only - http://bmjopen.bmj.com/site/about/guidelines.xhtml

Introduction

Stroke is a major cause of death and disability worldwide, with three-quarters (~6.3 million) of deaths each year occurring in developing countries including China.[1-3] In China, stroke is now the leading cause of death and disability, accounting for almost 2 million deaths in 2017.[3-5] While mortality rates from stroke have declined substantially over the last several decades in high-income countries, the annual costs for its hospital treatment and rehabilitation were 40 billion Chinese Yuan (CNY) and approximately 6 billion US dollars (USD) in 2016, which are 10-fold higher than those for ischemic heart disease (IHD).[3] The average per-patient cost of hospitalisation for stroke cases in China in 2018 was 14,291 Chinese Yuan (CNY) (9,409 CNY [1,309 USD] for ischemic stroke and 19,149 CNY [2,772 USD] for hemorrhagic stroke).

In recent decades, evidence-based guidelines for the management of different stroke types have been developed, regularly updated, and widely disseminated in China whereas Traditional Chinese Medicine (TCM), for which no randomised evidence exists, continues to be used extensively in the hospital management of stroke cases.[6] Nevertheless, there is still substantial variation in access to and use of evidence-based treatments for stroke in different regions of China.[6] While its treatment has been extensively studied in Western countries,[7-9] little is known about the contemporary use of evidence-based medical and surgical treatments for hospitalised cases of stroke in China. Previous studies of hospital management of stroke in China have been constrained by small sample sizes and involvement of only large teaching or tertiary hospitals, or restricted to studies in large urban areas only.[10-15]

For peer review only - http://bmjopen.bmj.com/site/about/guidelines.xhtml

We examined the use of medical and surgical treatments for first-ever incident stroke cases by stroke pathological type, overall and by sex, age of stroke onset, calendar year, hospital tier, region and other risk factors during an 11-year follow-up of 0.5 million adults in the China Kadoorie Biobank (CKB). [16,17]

Methods

Study population

Details of the study design and methods for recruitment and follow-up used in the CKB have been previously reported.[16,17] In brief, the 2004-8 baseline survey was conducted in 10 (5 urban, 5 rural) diverse areas of China, chosen to maximise regional differences in risk exposures and burden of major diseases. In each area, all permanent residents aged 35-74 years who were without disability were invited to participate. A total of 512,726 individuals (including some outside the target age range) agreed to participate in the study.

Data on socio-demographic status, smoking and alcohol consumption, diet, physical activity and medical history were collected by trained health workers using laptop-based questionnaires at local study clinics. All participants also had clinical measurements recorded, including height, weight, waist and hip circumference, lung function, blood pressure and heart rate and a blood sample collected. Ethical approval for the China Kadoorie Biobank was obtained from the Oxford University Tropical Research Ethics Committee and the Chinese Center for Disease Control and Prevention Ethical Review Committee. All participants provided written informed consent for participating and for accessing their medical records during follow-up by study investigators.

For peer review only - http://bmjopen.bmj.com/site/about/guidelines.xhtml

After the baseline survey, the vital status of participants was monitored regularly through Disease Surveillance Points (DSP) death registries, supplemented by annual checks with local residential records and active confirmation through local street committees or village administrators.[17] The occurrence of stroke among participants was identified by electronic linkage, using a unique personal identification number, to national health insurance (HI) systems that cover >97% of CKB participants, and to death and disease-specific (stroke, IHD, cancer and diabetes) registries accessible in study areas.

Key medical records of hospitalised cases of first-ever primary stroke reported to the study were retrieved and stroke diagnoses were verified by local trained CKB staff at 261 treating hospitals. The retrieved documents (e.g. initial covering page and discharge summary of the first admission record, with reports of brain imaging and use of pre-specified medical and surgical treatments for stroke) were reviewed electronically by 26 accredited Chinese neurologists and stroke physicians from Tier III (top ranking) hospitals in China using bespoke IT systems. While indicators of medical and surgical treatments were pre-specified at 3 time points (first 24 hours after admission, during hospital stay, and at discharge) in the medical notes, information on timing was incomplete and consequently separate analysis of drug treatment during each phase was not possible. Hence, recorded hospital treatment in any of the three periods was used in the present analysis.

For peer review only - http://bmjopen.bmj.com/site/about/guidelines.xhtml

Classification of stroke pathological types

All reported and adjudicated diagnoses of stroke pathological types were checked centrally by study staff and standardised by 10th Revision of the International Statistical Classification of Diseases and Related Health Problems (ICD-10) codes using bespoke software. The major stroke pathological types examined were ischemic stroke (IS; ICD-10 I63, including lacunar infarction [LACI] and non-lacunar infarction [non-LACI]), intracerebral hemorrhage (ICH; I61), subarachnoid hemorrhage (SAH; I60), and unspecified stroke (I64). IS was defined as focal neurological dysfunction lasting for more than 24 hours with or without neuroimaging evidence of a cerebral infarct. LACI was classified by the evidence of an infarct <1.5 cm in diameter on CT (computed tomography) or MR (magnetic resonance) brain imaging with focal neurological deficits, including lacunar syndrome.[18] Silent cerebral infarct (SCI) was characterised by imaging evidence of cerebral infarction in the absence of a focal neurological deficit. Non-LACI was defined as any IS type excluding LACI.

ICH was defined as a neurological dysfunction caused by a hemorrhage into the brain parenchyma or the ventricular system, excluding those induced by injury, with or without neuroimaging evidence of hemorrhage. SAH was defined as a neurological dysfunction considered to be caused by hemorrhage into the subarachnoid space, excluding those induced by injury, with or without relevant neuroimaging evidence. All SCI cases were also reviewed centrally by two research clinicians with relevant training in neurology and included with other stroke cases in an “any stroke” endpoint.

For peer review only - http://bmjopen.bmj.com/site/about/guidelines.xhtml

Patient and public involvement

Prior to recruitment in CKB, local community leaders in China were consulted. The study findings 19 are reported in peer-review publications and any relevant public health messages disseminated 20 using local press, television and internet.

Data sharing

Data from this study can be accessed by bona fide researchers upon reasonable request.

Statistical analysis

The main analyses of the present study were restricted to adjudicated first-ever incident cases of stroke occurring until 31 December 2017 among 484,413 participants with no prior heart disease and/or stroke/TIA at baseline (n=23,129) or prior stroke cases before the first adjudicated stroke (n=5,184). The rates of incident stroke cases were standardised to the overall CKB population by sex, area, and age at diagnosis. Rates of use of medical and surgical treatments were estimated separately for any stroke and for stroke pathological types, by sex, and area (rural vs urban), separately. All analyses were conducted using R 3.5.0.

For peer review only - http://bmjopen.bmj.com/site/about/guidelines.xhtml

Results

Characteristics of cases with different stroke types

Among 484,413 participants included for analysis, the mean age (SD) at baseline was 51.5 (10.5) years, 59% were women, and 43% lived in urban areas (**Table 1**). During 4.72 million person-years of follow-up, 61,902 incident cases of stroke were reported, including 57,190 (92%) hospitalised cases. Overall, there were 20,299 first-ever hospitalised cases of primary stroke for which each diagnosis was confirmed by clinical adjudication, including 17,306 (85%) with IS (7,123 non-LACI, 6,690 LACI, and 3,493 SCI), 2,623 ICH, and 370 SAH, respectively. Overall, 93% of reported stroke types (92% for IS, 97% for ICH and 96% for SAH) had brain imaging.

Compared with those without stroke, individuals who experienced first-ever stroke during follow-up were older and more likely to be male, to reside in urban areas, and to have lower levels of education and household income at baseline (**Table 1**). Individuals with first-ever ICH were more likely than those with other stroke types to be men, to live in rural areas, and to have lower levels of full-time education (54.8% no formal/primary school: **Table 1**) and have low annual household income (30.6% less than 10,000 CNY: **Table 1**). Likewise, individuals with ICH had the highest proportion with self-reported hypertension (23.5%: **Table1**), and the highest mean levels of systolic blood pressure (SBP) (149 mmHg: **Table 1**). With the exception of LACI cases (who were more likely to be female and live in urban areas), individuals with non-LACI and LACI shared broadly similar baseline characteristics (**Table 1**). Additionally, the characteristics of individuals with LACI

For peer review only - http://bmjopen.bmj.com/site/about/guidelines.xhtml

and SCI were comparable. However, compared to LACI, SCI cases had lower mean levels of SBP (133 mmHg vs 138 mmHg). The baseline characteristics of individuals with SAH were generally similar to those with ICH (**Table 1**). Interestingly, the overall prevalence of atrial fibrillation was very low, albeit higher in non-LACI than in LACI cases (0.3% vs 0.1%: **Table 2**).There were no significant differences in baseline characteristics between adjudicated and unadjudicated stroke cases (**eTable 1**).

Hospital treatment in IS cases

Overall, antiplatelet agents (aspirin or clopidogrel) were administered to 63.7% of all hospitalised IS cases, but use was higher in cases with LACI and non-LACI (66.3% and 65.1%, respectively) versus SCI (55.6%) (**Table 2**). Dual antiplatelet therapy was much less commonly used (non-LACI 8.3%, LACI 6.2%, and SCI 2.6%). Intravenous thrombolytic treatments (rtPA and urokinase) were used in 1.2% of non-LACI and 0.3% of LACI cases. Overall, 42% of all IS cases received blood pressure-lowering treatments, with similar proportions by IS types (42.2%, 41.8%, and 40.8% for LACI, non-LACI, and SCI, respectively). Likewise, lipid-lowering treatments were used in only about half of all hospitalised cases of IS (52.6%, 51.5%, and 43.2% for LACI, non-LACI, and SCI, respectively). In contrast, 56% of IS cases (62.0%, 49.9%, and 73.6%, for LACI, non-LACI, and SCI, respectively) received TCM (**Table 2**).

For antiplatelet and lipid-lowering medications as well as TCM, the rates of use for IS increased with age, while no such increasing trends with age were observed for blood-pressure lowering treatments **(Figure 1)**. All medical treatments for IS, with the exception

For peer review only - http://bmjopen.bmj.com/site/about/guidelines.xhtml

of blood pressure-lowering therapy, increased by calendar year of follow-up between 2005 and 2017 **(Figure 2)**. While the use of blood pressure-lowering treatments did not differ significantly by hospital tiers, the use of TCM was higher in Tier III hospitals and increased by calendar year between 2005 to 2017 (**Figure 2**). Similarly, age-specific use of antiplatelet, blood pressure-lowering, and lipid-lowering agents and TCM among IS cases did not vary appreciably by sex or area of residence (**eFigures 1-6**), but use of TCM increased by hospital tier in both men and women and in rural and urban areas. Carotid endarterectomy or stenting were very rarely undertaken (**Table 2**).

Hospital management of ICH and SAH

Among participants hospitalised with ICH, 3.9% had surgical clot evacuation. A small proportion of cases with SAH underwent endovascular coiling (7.0%) or neurosurgical clipping of an aneurysm (5.7%). Approximately half of ICH (52.6%) or SAH (50.5%) cases received blood pressure-lowering medication, which did not vary significantly by sex or area (**eFigure 1**). Overall, the use of lipid-lowering agents was 10.4% for ICH whereas approximately one-third of ICH (37.8%) and SAH (29.7%) cases received TCM while in hospital (**Table 3**).

For peer review only

Discussion

The present study, involving over 20,000 incident stroke cases, demonstrated that while there have been substantial improvements in the access to and use of brain imaging for diagnosis of stroke types in hospitalised cases, the use of many evidence-based medical treatments, including antiplatelet, blood pressure-lowering, and lipid-lowering agents, remains low in China, even in top-rank hospitals. In contrast, the use of TCM has remained consistently high, especially in higher ranking hospitals, highlighting the urgent need for better utilisation of evidence-based therapies, particularly in IS cases. Likewise, further trials are required to examine the efficacy and safety of such treatments in ICH cases. Importantly, such assessments of the use of TCM in the management of both IS and ICH are also indicated.[19]

Despite marked improvements in the hospital management of stroke in recent decades in China,[15] the use of evidence-based medications remains lower than in Western countries. Thrombolytic therapy (e.g. r-tPA), which was approved by the State Food and Drug Administration of China in 2001, was administered to <1% of potentially eligible cases in the present study, compared with 3.0-8.5% reported in the USA [20] and 1.6% reported by the Chinese National Stroke Registry (CNSR) in 2011.[21]

A recent American Heart Association registry study conducted during the same calendar period reported a higher use of antiplatelet agents in the USA vs China (83.9% vs 64% reported in the CKB).[22] Since data on common adverse events including bleeding were not recorded in the present study, the possibility that the lower use of antiplatelet therapy

For peer review only - http://bmjopen.bmj.com/site/about/guidelines.xhtml

was clinically indicated in this population cannot be entirely excluded. Likewise, the use of blood pressure-lowering and lipid-lowering medications was also higher in the USA compared with China (76.7% vs 41.7%, and 77.6% vs 50.3%, respectively).[22] Moreover, the use of drug treatment for secondary prevention of stroke in the CKB was significantly lower than that reported in the CNSR.[15] Reduction of blood pressure is the most important intervention for improving outcomes of cases with SAH and ICH.[23,24] However, in the present study, only about half of all ICH and SAH cases (and only 42% of IS cases) received blood pressure-lowering treatment. One explanation for the discrepancy between treatment rates among stroke cases in the CKB and the CNSR may be that the former study was conducted in 10 diverse regions (5 rural regions and 5 urban) with 21% of hospitals involving lower-grade (Tier 1) hospitals, whereas in the latter study cases were restricted to urban regions and included only higher-tier hospitals. Additionally, medication data in the present study were obtained from retrieved hospital admission records rather than at later periods of follow-up. Although in-hospital treatment data are reliable, intended medication use following hospital discharge may be underestimated.

While there is substantial underuse of evidence-based medical and surgical treatments for hospitalised stroke cases, TCM are widely used for all stroke types in China, particularly in Tier 3 hospitals, despite absent randomised evidence to support their efficacy and safety.[19,25] The overall rates of use of evidence-based medications were also lower among younger stroke cases consistent with previous reports.[26] The economic and social effects of stroke and its long-term sequelae in younger cases are considerable and these inequalities should be a priority for improvement. The suboptimal use of medical

For peer review only - http://bmjopen.bmj.com/site/about/guidelines.xhtml

treatments may partly explain the high recurrence rates of IS recently reported in this study population.[29]

Rates of medical therapy did not vary appreciably among ischemic stroke types (non-LACI, LACI, and SCI). However, relevant clinical guidelines address only the management of symptomatic stroke cases,[27,28] since there have been no randomised controlled trials including participants with SCI. The high prevalence of SCI – and its strong association with subsequent risks of symptomatic stroke, [29-31] cognitive decline and dementia, [32-34] psychiatric disorders,[35] and increased mortality,[36] highlight the need for new trials to assess the efficacy and safety of medical treatments in such cases.

The use of drug treatments has increased considerably in recent years, particularly after introduction of universal health coverage in 2009, reflecting the effects of health policy. A recent report of the temporal trends in hospital admissions for stroke in China demonstrated that admissions for first stroke increased by 3-6% per year after 2009 following adjustment for age, other risk factors and incidence of other diseases.[37] These stroke admissions did not, however, vary appreciably between rural and urban areas or between hospital tiers, suggesting that low socio-economic status, which is more common in rural areas, is unlikely to explain the suboptimal use of evidence-based treatments for stroke in China.

Further studies on the adverse effects of medications (e.g., bleeding risks with antiplatelet agents or rhabdomyolysis in high-dose statin users) are also needed to improve our understanding of the reduced rates of use of evidence-based therapies for IS in Chinese

For peer review only - http://bmjopen.bmj.com/site/about/guidelines.xhtml

populations. The consequences of their suboptimal use – and the relatively prevalent use of TCM – on short-term mortality and stroke recurrence following stroke also warrant further study.

The present study had several strengths, including the use of bespoke web-based adjudication systems to determine the diagnosis and clinical characteristics of over 20,000 reported stroke cases of all pathological types including SCI. Moreover, it included data from Tier I hospitals in rural areas that have not been included in previous studies. However, there were several limitations. Firstly, the CKB study was not a nationally representative study and, hence, the results cannot be representative of all areas in China. Secondly, it is possible that some selected clinical information relating to the acute phase of stroke (for example, administration of thrombolysis or aspirin treatment within 48 hours of hospital admission) was recorded in the emergency room and omitted in the retrieved medical records. Not all hospitals recorded medications for intended treatment after discharge in the medical records, which may have led to underestimates of their actual use. Lastly, the present study did not collect records of side effects of treatments (e.g., bleeding associated with the use of antiplatelet agents). Consequently, the relatively reduced observed rates of medical treatment may reflect clinical discretion and caution. However, the possibility of missing clinical data was minimised by the photographing of all available relevant medical records and enlistment of specialist neurologists and stroke physicians from Tier III hospitals to adjudicate the diagnoses of stroke types.

Overall, this contemporary study of 20,000 hospitalised stroke cases demonstrated that use of all evidence-based medical treatments increased substantially after the introduction

For peer review only - http://bmjopen.bmj.com/site/about/guidelines.xhtml

of the nationwide universal healthcare system in China. Despite recent improvements in stroke management, there is still considerable underuse of many evidence-based therapies and widespread overuse of TCM for stroke in the absence of available data on its efficacy and safety. Future studies should investigate the causes and consequences of these variations in clinical practice for stroke care in China.[38] Moreover, further large randomised trials are necessary to evaluate the efficacy and safety of established evidence-based stroke management for SCI.

For peer review only

For peer review only - http://bmjopen.bmj.com/site/about/guidelines.xhtml

Disclosures: None of the authors have any disclosures that were relevant to this report.

Contributorship

HQ, IT, YC and RC designed the study. NW concluded the statistical analyses. HQ wrote the first draft and IT, YC and RC revised the report, tables and figures. L Liu and YW provided critical advice on the analyses and interpretation. YG, LL and ZC supervised the overall conduct of the CKB study. All authors provided critical comments on the final report. RC and YC took responsibility for submission of the final version for publication.

Competing interests

None of the authors had any conflicts of interest in relation to this report.

Funding

UK Wellcome Trust	(212946/Z/18/Z, 202922/Z/16/Z, 104085/Z/14/Z, 088158/Z/09/Z)
National Key Research Development Project	(2016YFC0900500, 2016YFC0900501, 2016YFC0900504, 2016YFC1303904)
Chinese National Natural Science Foundation	(91843302)
UK Medical Research Council	(MC_UU_00017/1,MC_UU_12026/2 MC_U137686851)
British Heart Foundation	(CH/1996001/9454),
Cancer Research UK	(C16077/A29186; C500/A16896)

For peer review only

For peer review only - http://bmjopen.bmj.com/site/about/guidelines.xhtml

Ethical approval

Oxford	Kadoorie Study of Chronic disease in China (KSCDC) - Baseline, Oxford Tropical Research Ethics Committee (OxTREC) (2005) OxTREC Ref: 025-04
	China Kadoorie Biobank (A prospective study of environmental and genetic causes of premature death in 500,000 Chinese adults) – Second resurvey (2013) OXTREC Reference: 1024-13
China	Kadoorie Study of Chronic disease in China (KSCDC) – Baseline, Chinese Centre for Disease Control and Prevention. Ethical Review Committee (2004) Approval Notice 005/2004
	Kadoorie Study of Chronic disease in China (KSCDC) – Second Phase, Chinese Academy of Medical sciences/Peking Union Medical College, Ethical Committee (2010) 24th December 2010
	Kadoorie Study of Chronic disease in China (KSCDC) – Second Resurvey, Chinese Academy of Medical Sciences/Peking Union Medical College, Ethical Committee (2013) Ref: X1303262001

For peer review only

Figure Legends

Figure 1: Proportions of medical treatments in hospital by IS subtype

Figure 2: Antiplatelet, antihypertensive, lipid−lowering and TCM treatments for ischemic stroke by hospital tiers and calendar periods from 2005 to 2017

For peer review only

For peer review only - http://bmjopen.bmj.com/site/about/guidelines.xhtml

References

1. Feigin VL, Roth GA, Naghavi M, et al. Global burden of stroke and risk factors in 188 countries, during 1990-2013: a systematic analysis for the Global Burden of Disease Study 2013. Lancet Neurol 2016; **15**(9): 913-24.
2. Feigin VL, Krishnamurthi RV, Parmar P, et al. Update on the Global Burden of Ischemic and Hemorrhagic Stroke in 1990-2013: The GBD 2013 Study. Neuroepidemiology 2015; **45**(3): 161-76.
3. Chen WW, Gao RL, Liu LS, et al Summary of China Cardiovascular Diseases Report. *Chinese Circulation Journal;* **32** : 521-530
4. Institute for Health Metrics and Evaluation (IHME). GBD Compare. Seattle, WA: IHME,University of Washington, 2015. Available from http://vizhub.healthdata.org/gbd-compare. (accessed February 18, 2020).
5. China National Health Commission. China Health and Family Planning Statistical Yearbook 2019. *Peking Union Medical College Press* 2019.
6. Wu S, Wu B, Liu M, et al. Stroke in China: advances and challenges in epidemiology, prevention, and management. Lancet Neurol 2019; **18**(4): 394-405.
7. Bangalore S, Schwamm L, Smith EE, et al. Secondary Prevention after Ischemic Stroke or Transient Ischemic Attack. The American journal of medicine 2014.
8. Fonarow GC, Reeves MJ, Smith EE, et al. Characteristics, performance measures, and in-hospital outcomes of the first one million stroke and transient ischemic attack admissions in get with the guidelines-stroke. Circ Cardiovasc Qual Outcomes 2010; **3**(3): 291-302.
9. Feigin VL, Lawes CM, Bennett DA, Anderson CS. Stroke epidemiology: a review of population-based studies of incidence, prevalence, and case-fatality in the late 20th century. Lancet neurology 2003; **2**(1): 43-53.
10. Wang Y, Li Z, Zhao X, et al. Effect of a Multifaceted Quality Improvement Intervention on Hospital Personnel Adherence to Performance Measures in Patients With Acute Ischemic Stroke in China: A Randomized Clinical Trial. JAMA 2018; **320**(3): 245-54.
11. Heeley E, Anderson CS, Huang Y, et al. Role of health insurance in averting economic hardship in families after acute stroke in China. Stroke; a journal of cerebral circulation 2009; **40**(6): 2149-56.
12. Ding D, Lu CZ, Fu JH, Hong Z. Predictors of vascular events after ischemic stroke: the China ischemic stroke registry study. Neuroepidemiology 2010; **34**(2): 110-6.
13. Liu X, Xu G, Wu W, Zhang R, Yin Q, Zhu W. Subtypes and one-year survival of first-ever stroke in Chinese patients: The Nanjing Stroke Registry. Cerebrovasc Dis 2006; **22**(2-3): 130-6.
14. He Q, Wu C, Luo H, et al. Trends in In-Hospital Mortality among Patients with Stroke in China. PloS one 2014; **9**(3): e92763.
15. Li Z, Wang C, Zhao X, et al. Substantial Progress Yet Significant Opportunity for Improvement in Stroke Care in China. Stroke 2016; **47**(11): 2843-9.
16. Chen Z, Lee L, Chen J, et al. Cohort profile: the Kadoorie Study of Chronic Disease in China (KSCDC). Int J Epidemiol 2005; **34**(6): 1243-9.

For peer review only - http://bmjopen.bmj.com/site/about/guidelines.xhtml

17. Chen Z, Chen J, Collins R, et al. China Kadoorie Biobank of 0.5 million people: survey methods, baseline characteristics and long-term follow-up. Int J Epidemiol 2011; **40**(6): 1652-66.
18. Asdaghi N, Jeerakathil T, Hameed B, et al. Oxfordshire community stroke project classification poorly differentiates small cortical and subcortical infarcts. Stroke 2011; **42**(8): 2143-8.
19. Chen Z, Sandercock P, Xie JX, Peto R, Collins R, Liu LS. Hospital management of acute ischemic stroke in China. Journal of stroke and cerebrovascular diseases : the official journal of National Stroke Association 1997; **6**(5): 361-7.
20. Reeves MJ, Arora S, Broderick JP, et al. Acute stroke care in the US: results from 4 pilot prototypes of the Paul Coverdell National Acute Stroke Registry. Stroke; a journal of cerebral circulation 2005; **36**(6): 1232-40.
21. Wang Y, Liao X, Zhao X, et al. Using recombinant tissue plasminogen activator to treat acute ischemic stroke in China: analysis of the results from the Chinese National Stroke Registry (CNSR). Stroke 2011; **42**(6): 1658-64.
22. Bushnell CD, Olson DM, Zhao X, et al. Secondary preventive medication persistence and adherence 1 year after stroke. Neurology 2011; **77**(12): 1182-90.
23. Connolly ES, Jr., Rabinstein AA, Carhuapoma JR, et al. Guidelines for the management of aneurysmal subarachnoid hemorrhage: a guideline for healthcare professionals from the American Heart Association/american Stroke Association. Stroke 2012; **43**(6): 1711-37.
24. Hemphill JC, 3rd, Greenberg SM, Anderson CS, et al. Guidelines for the Management of Spontaneous Intracerebral Hemorrhage: A Guideline for Healthcare Professionals From the American Heart Association/American Stroke Association. Stroke 2015; **46**(7): 2032-60.
25. Wei JW, Huang Y, Wang JG, et al. Current management of intracerebral hemorrhage in China: a national, multi-centre, hospital register study. BMC Neurol 2011; **11**: 16.
26. Sappok T, Faulstich A, Stuckert E, Kruck H, Marx P, Koennecke HC. Compliance with secondary prevention of ischemic stroke: a prospective evaluation. Stroke 2001; **32**(8): 1884-9.
27. Kernan WN, Ovbiagele B, Black HR, et al. Guidelines for the prevention of stroke in patients with stroke and transient ischemic attack: a guideline for healthcare professionals from the American Heart Association/American Stroke Association. Stroke 2014; **45**(7): 2160-236.
28. Smith EE, Saposnik G, Biessels GJ, et al. Prevention of Stroke in Patients With Silent Cerebrovascular Disease: A Scientific Statement for Healthcare Professionals From the American Heart Association/American Stroke Association. Stroke 2017; **48**(2): e44-e71.
29. Fanning JP, Wong AA, Fraser JF. The epidemiology of silent brain infarction: a systematic review of population-based cohorts. BMC Med. 2014;12:119.
30. Gupta A, Giambrone AE, Gialdini G, Finn C, Delgado D, Gutierrez J, et al. Silent Brain Infarction and Risk of Future Stroke: A Systematic Review and Meta-Analysis. Stroke. 2016;47(3):719-25.
31. Vermeer SE, Hollander M, van Dijk EJ, Hofman A, Koudstaal PJ, Breteler MM. Silent brain infarcts and white matter lesions increase stroke risk in the general population: the Rotterdam Scan Study. Stroke. 2003;34(5):1126-9.
32. Vermeer SE, Prins ND, den Heijer T, Hofman A, Koudstaal PJ, Breteler MM. Silent brain infarcts and the risk of dementia and cognitive decline. N Engl J Med. 2003;348(13):1215-22.

For peer review only - http://bmjopen.bmj.com/site/about/guidelines.xhtml

33. Bos D, Wolters FJ, Darweesh SKL, Vernooij MW, de Wolf F, Ikram MA, et al. Cerebral small vessel disease and the risk of dementia: A systematic review and meta-analysis of population-based evidence. Alzheimers Dement. 2018;14(11):1482-92.
34. Smith EE, O'Donnell M, Dagenais G, Lear SA, Wielgosz A, Sharma M, et al. Early cerebral small vessel disease and brain volume, cognition, and gait. Ann Neurol. 2015;77(2):251-61.
35. van Agtmaal MJM, Houben A, Pouwer F, Stehouwer CDA, Schram MT. Association of Microvascular Dysfunction With Late-Life Depression: A Systematic Review and Meta-analysis. JAMA Psychiatry. 2017;74(7):729-39.
36. Bokura H, Kobayashi S, Yamaguchi S, Iijima K, Nagai A, Toyoda G, et al. Silent brain infarction and subcortical white matter lesions increase the risk of stroke and mortality: a prospective cohort study. J Stroke Cerebrovasc Dis. 2006;15(2):57-63.
37.. Chen Y, Wright N, Guo Y, et al Mortality and recurrent vascular events after first incident stroke: a 9-year community-based study of 0·5 million Chinese adults. *Lancet Global Health* 2020, 8(4):e580-90
38. Levy M, Chen Y, Clarke R et al Socioeconomic differences in health-care use and outcomes for stroke and ischemic heart disease in China during 2009–16: a prospective cohort study of 0·5 million adults*; Lancet Global Health* 2020; 8(4): e591-e602

For peer review only - http://bmjopen.bmj.com/site/about/guidelines.xhtml

Table 1: Selected baseline characteristics of participants by types and subtypes of first adjudicated stroke

	IS			ICH	SAH	Any stroke	All CKB
	Non-LACI	LACI	Silent				
	(n = 7,123)	(n = 6,690)	(n = 3,493)	(n = 2,623)	(n = 370)	(n = 20,299)	(n = 484,413)
**Age, %**							
30 – 39	2.0	1.8	2.0	4.4	6.3	2.4	16.0
40 – 49	13.1	14.3	13.4	16.9	21.4	14.5	31.0
50 – 59	32.5	34.3	33.0	34.6	36.6	33.8	30.8
60 – 69	35.5	34.4	35.1	30.0	24.8	33.9	16.6
70 – 79	16.9	15.2	16.4	14.2	11.0	15.5	5.7
Mean (SD)	60.1 (9.6)	59.6 (9.5)	60.0 (9.6)	58.3 (10.0)	56.5 (10.2)	59.5 (9.7)	51.5 (10.5)
**Sex, %**							
Male	50.0	46.5	39.0	53.1	36.9	47.4	40.8
Female	50.0	52.3	59.8	46.9	60.3	52.6	59.2
**Area, %**							
Rural	58.0	40.6	39.0	63.3	51.6	49.2	56.8
Urban	42.0	59.4	61.0	36.7	48.4	50.8	43.2
**Highest education, %**							
No formal / primary school	54.4	52.4	53.2	54.8	46.4	53.4	50.7
Middle / high school	40.1	42.4	38.8	40.3	40.1	42.3	43.6
College / University	4.0	4.1	4.8	4.1	5.6	4.3	5.7
**Annual household income (RMB), %**							
<9,999	30.1	27.3	29.6	30.6	29.6	29.2	28.3
10,000-19,999	29.4	33.5	30.1	30.6	24.0	30.7	28.8
20,000-34,999	22.9	21.0	21.4	24.3	23.5	23.6	24.7
≥35000	16.0	17.0	15.9	13.7	15.1	16.5	18.1
**Current smoker, %**							
Males	69.4	67.1	55.1	62.5	52.1	66.5	62.0
Female	3.5	3.6	1.8	2.2	4.3	3.0	2.3
**Current alcohol drinker, %**							

For peer review only - http://bmjopen.bmj.com/site/about/guidelines.xhtml

Males	35.2	38.6	33.9	38.8	24.8	36.5	33.9
Female	2.5	1.4	2.0	3.7	1.3	2.5	2.1
**Regular dietary intake †, %**							
Meat	79.2	82.0	79.1	81.7	72.3	82.3	82.9
Fish	44.8	45.4	42.4	42.8	41.6	45.6	46.8
Dairy	16.8	18.6	19.5	16.2	19.1	18.3	19.7
Fruit	55.1	54.8	54.9	54.8	58.6	56.3	59.5
**Prevalent disease ‡, %**							
Diabetes	6.8	5.9	4.1	3.2	2.0	5.3	2.7
Hypertension	21.8	19.3	19.3	23.5	14.3	20.8	9.7
**Clinical measures, mean (SD)**							
SBP (mmHg)	140.3 (23.9)	138.4 (23.9)	132.8 (22.6)	149.0 (24.5)	124.7 (22.6)	142.1 (24.2)	130.4 (20.9)
BMI (kg/m^2)	24.3 (3.6)	24.0 (3.4)	23.3 (3.4)	24.0 (3.6)	21.7 (3.5)	24.4 (3.5)	23.6 (3.4)
RPG (mmol/L)	6.8 (3.5)	6.4 (3.3)	6.1 (2.9)	6.2 (2.9)	5.7 (3.2)	6.5 (3.2)	6.0 (2.3)
**Hospital tier, %**							
Tier 1 (162 hospitals) *	2.7	4.5	2.6	2.6	3.4	3.2	
Tier 2 (53 hospitals)	4.2	6.2	3.7	4.7	3.1	4.7	
Tier 3 (46 hospitals)	91.6	88.1	90.6	91.9	85.7	92.0	

Means and percentages are directly standardised to age, sex and study area structure of the CKB study population after excluding 23,129 participants with prior CHD or stroke/TIA and 5,184 participants with a reported stroke prior to first adjudicated stroke, as appropriate.

† 1-3 days per week or more often

‡ Self-reported at baseline survey

* includes 126 community health centres

Abbreviations: LACI: lacunar infarction, SD: standard deviation, SBP: systolic blood pressure, BMI: body mass index, RPG: random plasma glucose

For peer review only

For peer review only - http://bmjopen.bmj.com/site/about/guidelines.xhtml

Table 2: Hospital managements for subtypes of IS

Treatment, n (%)	Non-LACI (n = 7,123)		LACI (n = 6,690)		Silent (n = 3,492)		All IS (n = 17,305)		P
**Any antiplatelet agent**	4,638	(65.1)	4,434	(66.3)	1,943	(55.6)	11,015	(63.7)	<0.001
Aspirin	3,829	(53.8)	3,722	(55.6)	1,761	(50.4)	9,312	(53.8)	<0.001
Clopidogrel	456	(6.4)	431	(6.4)	132	(3.8)	1,019	(5.9)	<0.001
Dual antiplatelet therapy	589	(8.3)	413	(6.2)	90	(2.6)	1,092	(6.3)	<0.001
**Anticoagulant agent**	689	(9.7)	569	(8.5)	146	(4.2)	1,404	(8.1)	<0.001
**Any antihypertensive agent**	2,976	(41.8)	2,820	(42.2)	1,426	(40.8)	7,222	(41.7)	0.439
1 Antihypertensive agent	2,217	(31.1)	2,137	(31.9)	1,046	(30.0)	5,400	(31.2)	0.119
2 Antihypertensive agent	746	(10.5)	668	(10.0)	354	(10.1)	1,768	(10.2)	0.629
≥3 Antihypertensive agent	170	(2.4)	156	(2.3)	110	(3.2)	436	(2.5)	0.028
**Any lipid-lowering agent**	3,670	(51.5)	3,522	(52.6)	1,508	(43.2)	8,700	(50.3)	<0.001
Statin	3,601	(50.6)	3,441	(51.4)	1,461	(41.8)	8,503	(49.1)	<0.001
Other lipid lowering agent	133	(1.9)	130	(1.9)	63	(1.8)	326	(1.9)	0.879
**Thrombolytic Therapy**									<0.001
r-tPA	25	(0.4)	5	(0.1)	0	(0.0)	30	(0.2)	<0.001
Urokinase	60	(0.8)	15	(0.2)	0	(0.0)	75	(0.4)	<0.001
**Defibrase**	192	(2.7)	179	(2.7)	43	(1.2)	414	(2.4)	<0.001
**Any TCM**	3,545	(49.8)	4,151	(62.0)	2,571	(73.6)	10,267	(59.3)	0.095
**Surgery**	18	(0.3)	9	(0.1)	3	(0.1)	30	(0.2)	0.350
Carotid endarterectomy	0	(0.0)	2	(0.0)	1	(0.0)	3	(0.0)	0.454
Stenting	9	(0.1)	5	(0.1)	2	(0.1)	16	(0.1)	0.008
**Atrial fibrillation**	24	(0.3)	8	(0.1)	4	(0.1)	36	(0.2)	<0.001

For peer review only - http://bmjopen.bmj.com/site/about/guidelines.xhtml

Table 3: Hospital management for ICH and SAH

Hospital management, n (%)	ICH (n = 2,623)		SAH (n = 370)	
**Brain Imaging (CT or MRI)**		(97.0)		(96.0)
**Any antihypertensive agent**	1,379	(52.6)	187	(50.5)
1 Antihypertensive agent	899	(34.3)	146	(39.5)
2 Antihypertensive agent	436	(16.6)	42	(11.4)
≥3 Antihypertensive agent	151	(5.8)	8	(2.2)
**Any lipid-lowering agent**	272	(10.4)	12	(3.2)
Statin	266	(10.1)	12	(3.2)
Other lipid lowering agent	8	(0.3)	0	(0.0)
**Any TCM**	992	(37.8)	110	(29.7)
**Surgery**	230	(8.8)	47	(12.7)
Clot evacuation	103	(3.9)	3	(0.8)
Coiling	0	(0.0)	26	(7.0)
Aneurysm clipping surgery	0	(0.0)	21	(5.7)

For peer review only

For peer review only - http://bmjopen.bmj.com/site/about/guidelines.xhtml

Figure 1: Proportions of medical treatments in hospital by IS subtype

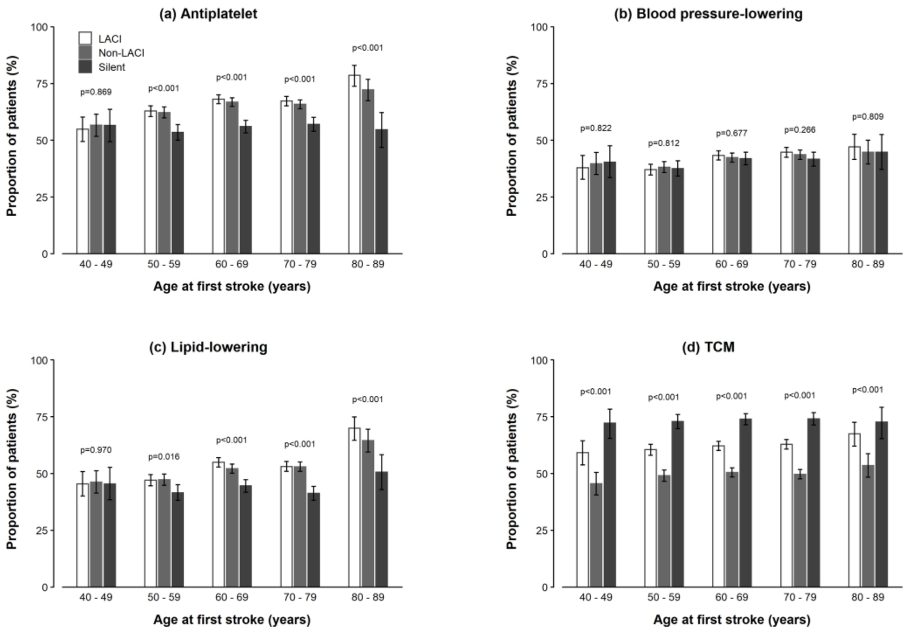

296x209mm (300 x 300 DPI)

Figure 2: Antiplatelet, antihypertensive, lipid-lowering and TCM treatments for ischaemic stroke by hospital tiers and calendar periods from 2005 to 2017

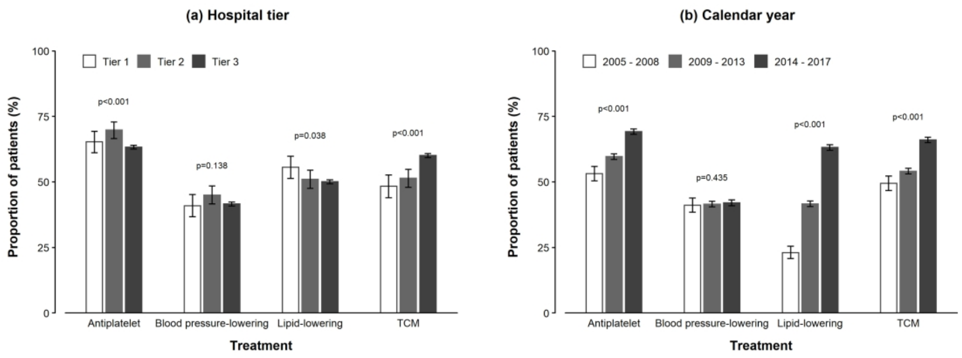

296x209mm (300 x 300 DPI)

Supplementary Material for "Hospital management of major stroke types in Chinese adults: a population-based study of 20,000 hospitalised stroke cases

eTable 1: Selected baseline characteristics of participants by adjusted, unadjusted stroke patients.

eFigure 1: Proportions of use of antiplatelet treatments in hospital by IS subtype, sex and area

eFigure 2: Proportions of use of blood pressure-lowering treatments in hospital by IS subtype, sex and area

eFigure 3: Proportions of use of lipid-lowering treatments in hospital by IS subtype, sex and area

eFigure 4: Proportions of us of TCM in hospital by IS subtype, sex and area

eFigure 5: Use of antiplatelet, antihypertensive, lipid-lowering and TCM treatments for ischaemic stroke by hospital tiers, sex and area

eFigure 6: Use of antiplatelet, antihypertensive, lipid-lowering and TCM treatments for ischaemic stroke by calendar periods from 2005 to 2017, sex and area

For peer review only

eTable 1: Selected baseline characteristics of participants by adjudicated, unadjusted stroke patients

	Adjudicated stroke (n = 20,299)	Unadjudicated stroke (n = 26,965)
**Age, %**		
30 – 39	2.4	3.3
40 – 49	14.5	15.1
50 – 59	33.8	32.4
60 – 69	33.9	33.2
70 – 79	15.5	15.9
Mean (SD)	59.5 (9.7)	59.2 (10.0)
**Sex, %**		
Male	47.4	43.7
Female	52.6	56.3
**Area, %**		
Rural	49.2	58.3
Urban	50.8	41.7
**Highest education, %**		
No formal / primary school	53.4	54.1
Middle / high school	42.3	41.3
College / University	4.3	4.6
**Annual household income (RMB), %**		
<9,999	29.2	29.9
10,000-19,999	30.7	30.1
20,000-34,999	23.6	23.6
≥35000	16.5	16.5
**Current smoker, %**		
Males	66.5	63.1
Female	3.0	2.8
**Current alcohol drinker, %**		
Males	36.5	35.0
Female	2.5	2.0
**Regular dietary intake †, %**		
Meat	82.3	82.7
Fish	45.6	45.7
Dairy	18.3	18.5
Fruit	56.3	57.9
**Prevalent disease ‡, %**		
Diabetes	5.3	4.9
Hypertension	20.8	18.1
**Clinical measures, mean (SD)**		
SBP (mmHg)	142.1 (24.2)	138.0 (24.9)
BMI (kg/m^2)	24.4 (3.5)	24.1 (3.5)
RPG (mmol/L)	6.5 (3.2)	6.4 (3.1)

Means and percentages are directly standardised to age, sex and study area structure of the CKB study population after excluding 23,129 participants with prior CHD or stroke/TIA and 5,184 participants with a reported stroke prior to first adjudicated stroke, as appropriate.
† 1-3 days per week or more often
‡ Self-reported at baseline survey
Abbreviations: LACI: lacunar infarction, SD: standard deviation, SBP: systolic blood pressure, BMI: body mass index, RPG: random plasma glucose

For peer review only

eFigure 1: Proportions of use of antiplatelet treatments in hospital by IS subtype, sex and area

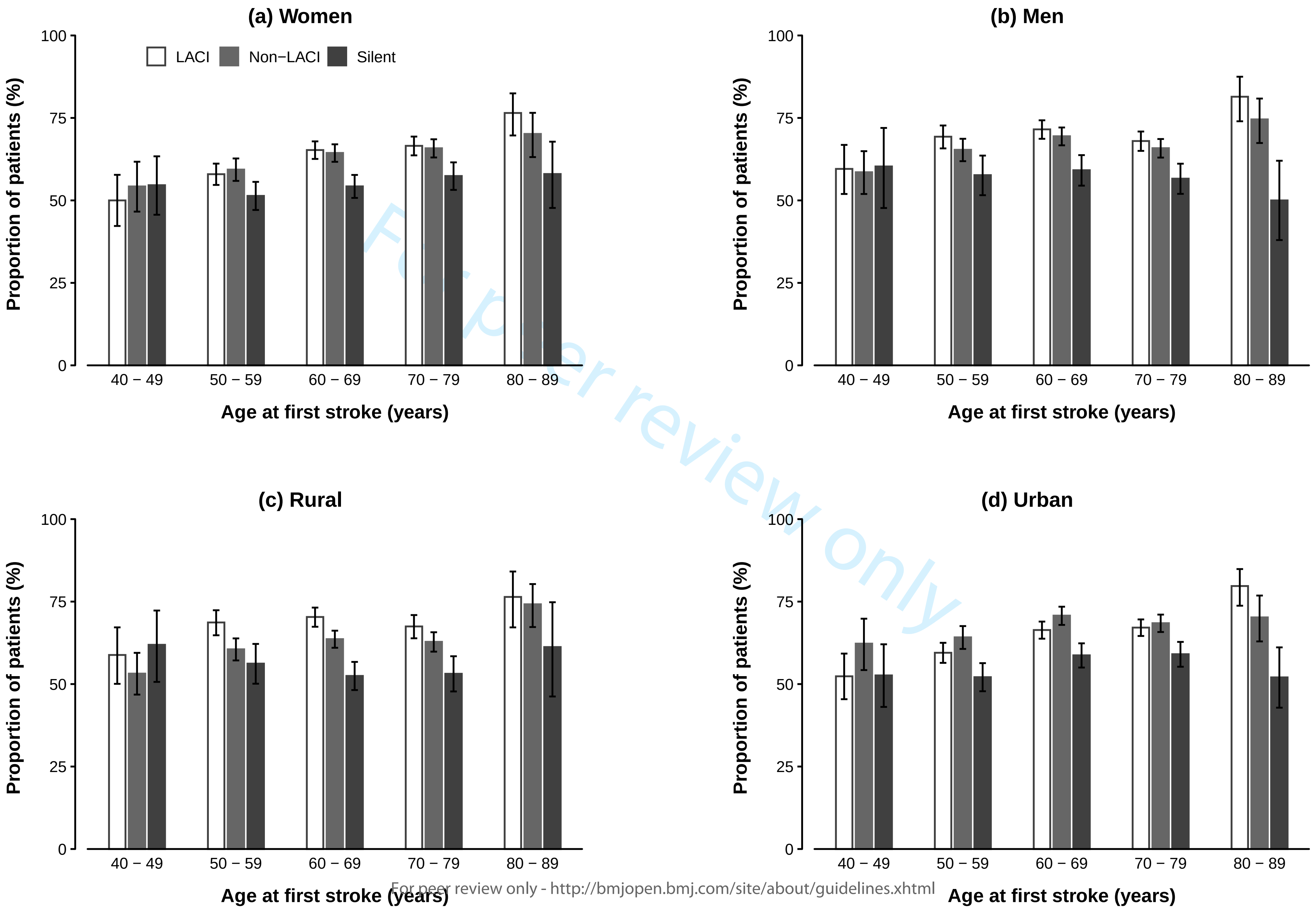

For peer review only

eFigure 2: Proportions of use of blood pressure−lowering treatments in hospital by IS subtype, sex and area

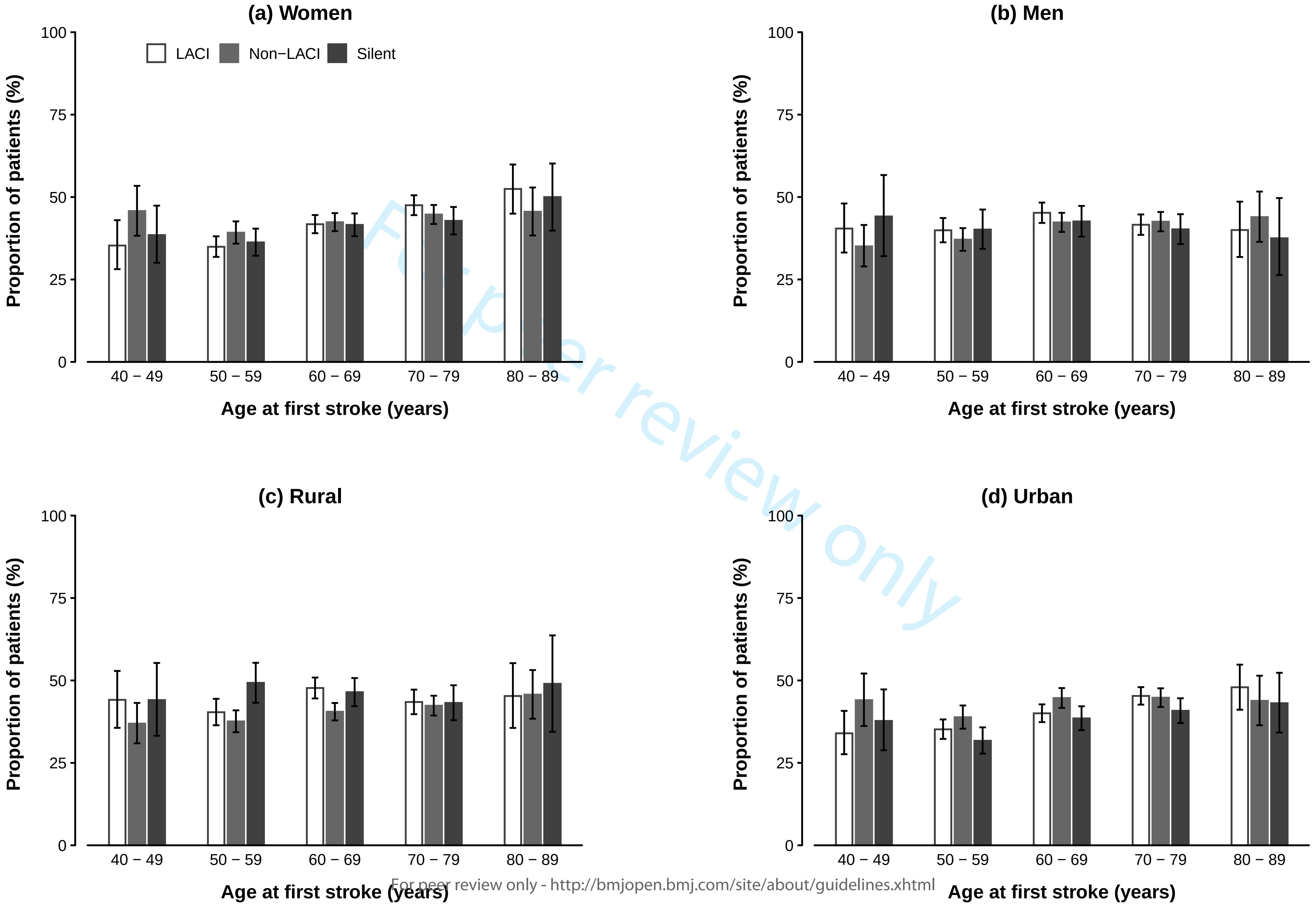

For peer review only

eFigure 3: Proportions of use of lipid−lowering treatments in hospital by IS subtype, sex and area

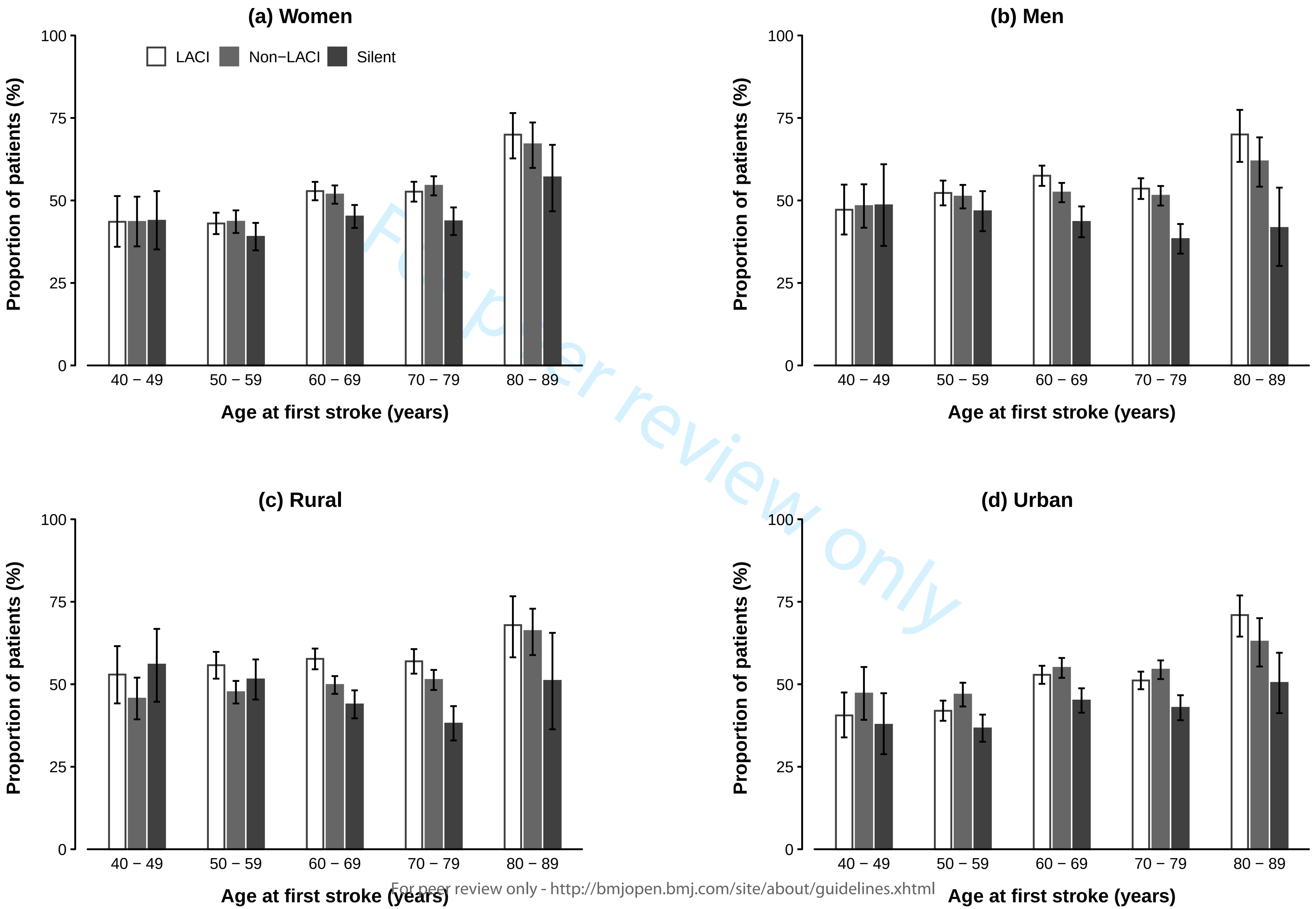

For peer review only

eFigure 4: Proportions of use of TCM in hospital by IS subtype, sex and area

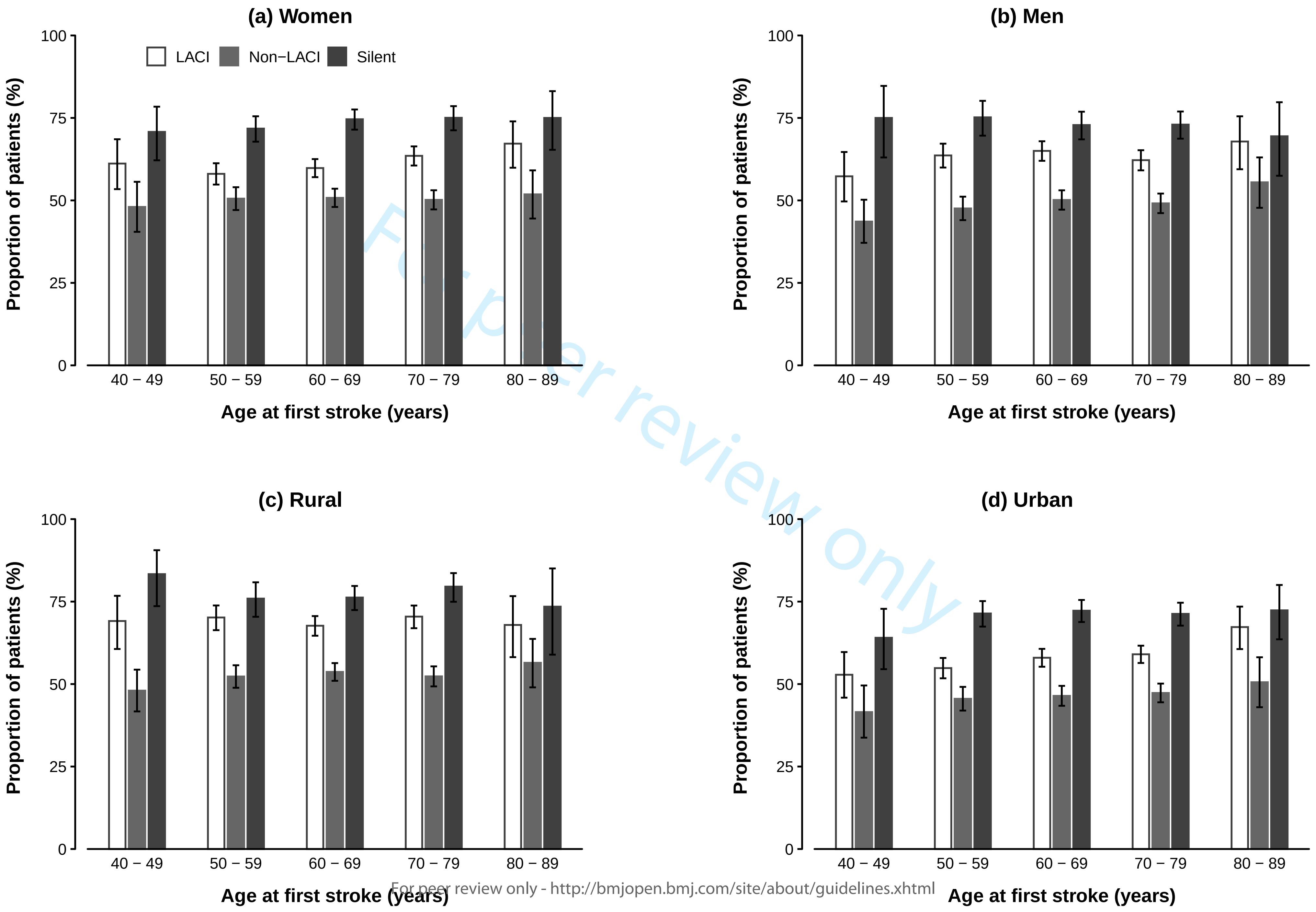

For peer review only

eFigure 5: Use of antiplatelet, antihypertensive, lipid−lowering and TCM treatments for ischaemic stroke by hospital tiers, sex and area

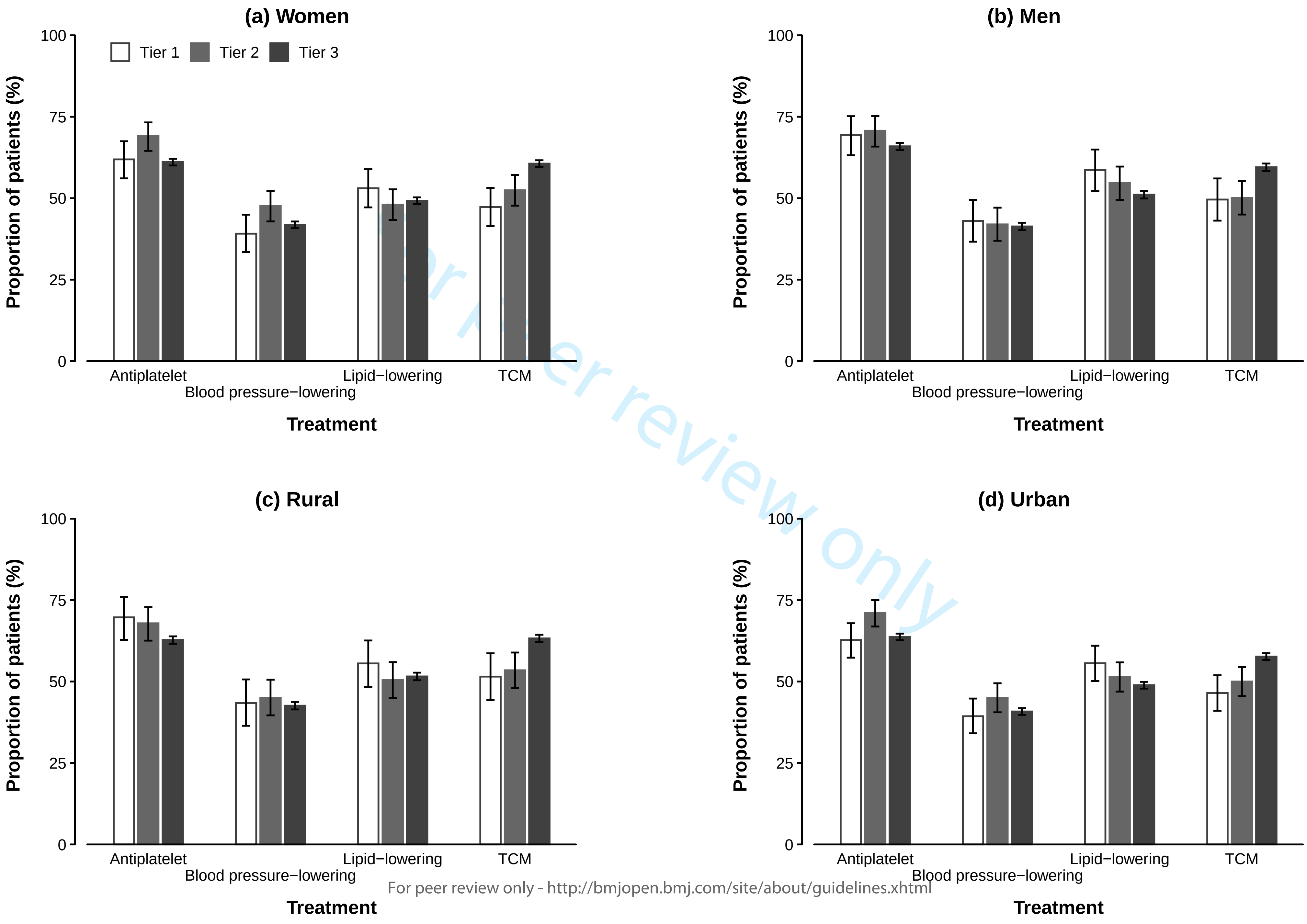

eFigure 6: Use of antiplatelet, antihypertensive, lipid−lowering and TCM treatments for ischaemic stroke by calendar periods from 2005 to 2017, sex and area

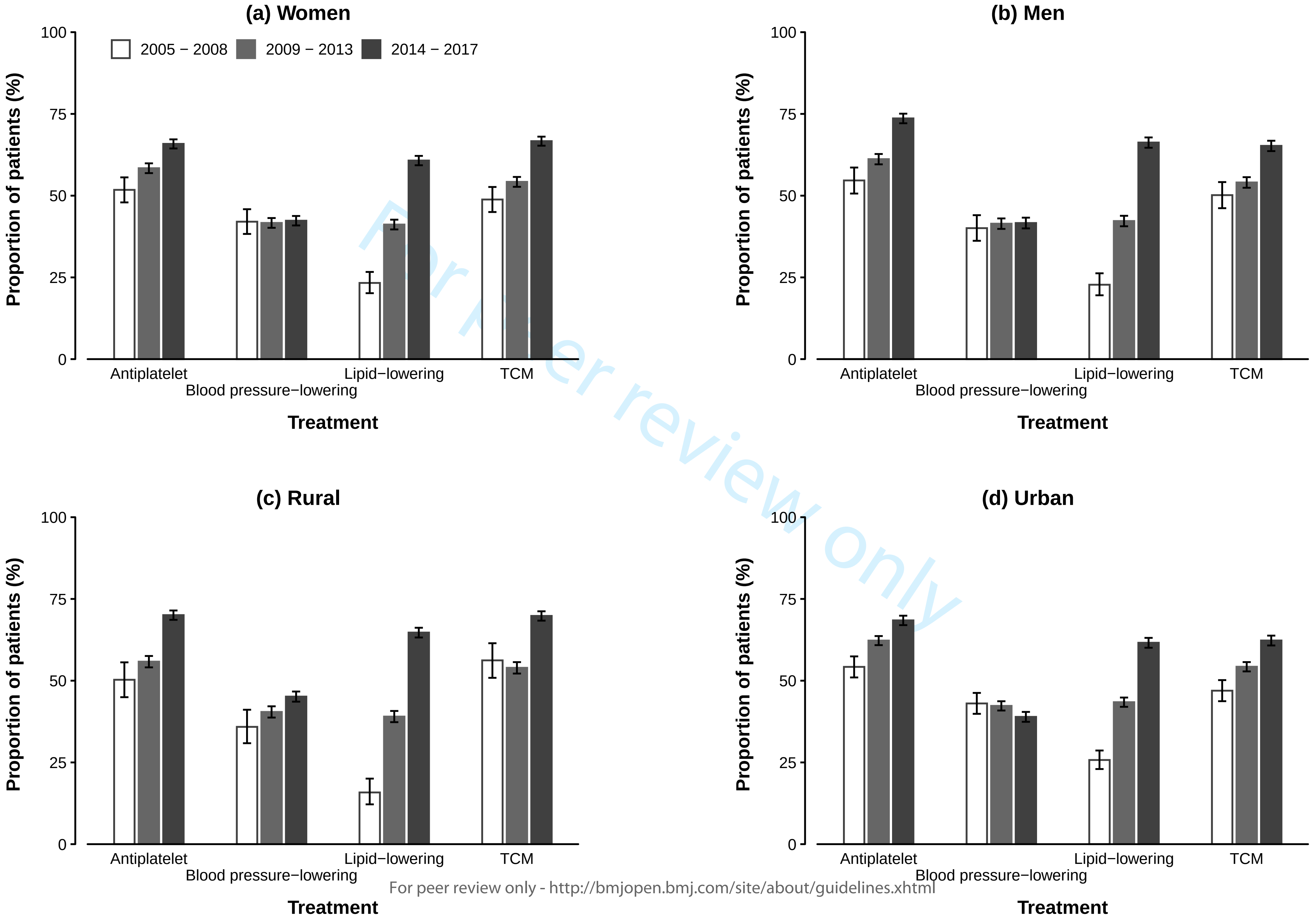

For peer review only

STROBE Statement—checklist of items that should be included in reports of observational studies

	Item No	Recommendation	Page No
**Title and abstract**	1	(*a*) Indicate the study's design with a commonly used term in the title or the abstract	1
		(*b*) Provide in the abstract an informative and balanced summary of what was done and what was found	2
**Introduction**			
Background/rationale	2	Explain the scientific background and rationale for the investigation being reported	3
Objectives	3	State specific objectives, including any prespecified hypotheses	3
**Methods**			
Study design	4	Present key elements of study design early in the paper	4
Setting	5	Describe the setting, locations, and relevant dates, including periods of recruitment, exposure, follow-up, and data collection	4
Participants	6	*(a) Cohort study*—Give the eligibility criteria, and the sources and methods of selection of participants. Describe methods of follow-up<br>*Case-control study*—Give the eligibility criteria, and the sources and methods of case ascertainment and control selection. Give the rationale for the choice of cases and controls<br>*Cross-sectional study*—Give the eligibility criteria, and the sources and methods of selection of participants	4-5
		(b) Cohort study—For matched studies, give matching criteria and number of exposed and unexposed<br>*Case-control study*—For matched studies, give matching criteria and the number of controls per case	
Variables	7	Clearly define all outcomes, exposures, predictors, potential confounders, and effect modifiers. Give diagnostic criteria, if applicable	5-6
Data sources/ measurement	8*	For each variable of interest, give sources of data and details of methods of assessment (measurement). Describe comparability of assessment methods if there is more than one group	*5-6*
Bias	9	Describe any efforts to address potential sources of bias	5
Study size	10	Explain how the study size was arrived at	4
Quantitative variables	11	Explain how quantitative variables were handled in the analyses. If applicable, describe which groupings were chosen and why	
Statistical methods	12	(a) Describe all statistical methods, including those used to control for confounding	7
		() Describe any methods used to examine subgroups and interactions	7
		(a) Explain how missing data were addressed	7
		(b) Cohort study—If applicable, explain how loss to follow-up was addressed<br>*Case-control study*—If applicable, explain how matching of cases and controls was addressed<br>*Cross-sectional study*—If applicable, describe analytical methods taking account of sampling strategy	7
		(b) Describe any sensitivity analyses	

Continued on next page

For peer review only - http://bmjopen.bmj.com/site/about/guidelines.xhtml

**Results**			
Participants	13*	(a) Report numbers of individuals at each stage of study—eg numbers potentially eligible, examined for eligibility, confirmed eligible, included in the study, completing follow-up, and analysed	7
		(b) Give reasons for non-participation at each stage	
		(c) Consider use of a flow diagram	
Descriptive data	14*	(a) Give characteristics of study participants (eg demographic, clinical, social) and information on exposures and potential confounders	7-8
		(b) Indicate number of participants with missing data for each variable of interest	
		(c) Cohort study—Summarise follow-up time (eg, average and total amount)	
Outcome data	15*	*Cohort study*—Report numbers of outcome events or summary measures over time	*8-10*
		Case-control study—Report numbers in each exposure category, or summary measures of exposure	
		Cross-sectional study—Report numbers of outcome events or summary measures	
Main results	16	(a) Give unadjusted estimates and, if applicable, confounder-adjusted estimates and their precision (eg, 95% confidence interval). Make clear which confounders were adjusted for and why they were included	8-10
		(b) Report category boundaries when continuous variables were categorized	
		(c) If relevant, consider translating estimates of relative risk into absolute risk for a meaningful time period	
Other analyses	17	Report other analyses done—eg analyses of subgroups and interactions, and sensitivity analyses	
**Discussion**			
Key results	18	Summarise key results with reference to study objectives	10-14
Limitations	19	Discuss limitations of the study, taking into account sources of potential bias or imprecision. Discuss both direction and magnitude of any potential bias	13-14
Interpretation	20	Give a cautious overall interpretation of results considering objectives, limitations, multiplicity of analyses, results from similar studies, and other relevant evidence	14
Generalisability	21	Discuss the generalisability (external validity) of the study results	14
**Other information**			
Funding	22	Give the source of funding and the role of the funders for the present study and, if applicable, for the original study on which the present article is based	Author statement

*Give information separately for cases and controls in case-control studies and, if applicable, for exposed and unexposed groups in cohort and cross-sectional studies.

Note: An Explanation and Elaboration article discusses each checklist item and gives methodological background and published examples of transparent reporting. The STROBE checklist is best used in conjunction with this article (freely available on the Web sites of PLoS Medicine at http://www.plosmedicine.org/, Annals of Internal Medicine at http://www.annals.org/, and Epidemiology at http://www.epidem.com/). Information on the STROBE Initiative is available at www.strobe-statement.org.

For peer review only - http://bmjopen.bmj.com/site/about/guidelines.xhtml

BMJ Open

Hospital management of major stroke types in Chinese adults: a population-based study of 20,000 hospitalised stroke cases

Journal:	*BMJ Open*
Manuscript ID	bmjopen-2021-054265.R1
Article Type:	Original research
Date Submitted by the Author:	11-Oct-2021
Complete List of Authors:	Qin, Haiqiang; China National Clinical Research Centre for Neurological Diseases, Tiantian Hospital Beijing<br>Turnbull, Iain; University of Oxford, Clinical Trial Service Unit and Epidemiological Studies, NDPH<br>Chen, Yiping; University of Oxford, Clinical Trial Service Unit and Epidemiological Studies, NDPH<br>Wright, Neil ; University of Oxford, Clinical Trial Service Unit and Epidemiological Studies, NDPH<br>Liu, Liping; China National Clinical Research Centre for Neurological Diseases, Tiantian Hospital Beijing, Department of Neurology<br>Pei, Pei; Chinese Academy of Medical Sciences and Peking Union Medical College<br>Tang, Wei; Pengzhou Traditional Chinese Medical Hospital<br>Xiang, Shengping; Pengzhou Traditional Chinese Medical Hospital<br>Guo, Yu; Chinese Academy of Medical Sciences and Peking Union Medical College<br>Zhao, Xingquan; China National Clinical Research Centre for Neurological Diseases, Tiantian Hospital Beijing<br>Clarke, Robert; University of Oxford,<br>Li, Liming; Chinese Academy of Medical Sciences and Peking Union Medical College<br>Wang, Yongjun; China National Clinical Research Centre for Neurological Diseases, Tiantian Hospital Beijing<br>Chen, Zhengming; University of Oxford, Clinical Trial Service Unit and Epidemiological Studies, NDPH
**Primary Subject Heading**:	Epidemiology
Secondary Subject Heading:	Cardiovascular medicine
Keywords:	Neurology < INTERNAL MEDICINE, EPIDEMIOLOGY, Health policy < HEALTH SERVICES ADMINISTRATION & MANAGEMENT, STROKE MEDICINE, Herbal medicine < THERAPEUTICS

I, the Submitting Author has the right to grant and does grant on behalf of all authors of the Work (as defined in the below author licence), an exclusive licence and/or a non-exclusive licence for contributions from authors who are: i) UK Crown employees; ii) where BMJ has agreed a CC-BY licence shall apply, and/or iii) in accordance with the terms applicable for US Federal Government officers or employees acting as part of their official duties; on a worldwide, perpetual, irrevocable, royalty-free basis to BMJ Publishing Group Ltd ("BMJ") its licensees and where the relevant Journal is co-owned by BMJ to the co-owners of the Journal, to publish the Work in this journal and any other BMJ products and to exploit all rights, as set out in our licence.

The Submitting Author accepts and understands that any supply made under these terms is made by BMJ to the Submitting Author unless you are acting as an employee on behalf of your employer or a postgraduate student of an affiliated institution which is paying any applicable article publishing charge ("APC") for Open Access articles. Where the Submitting Author wishes to make the Work available on an Open Access basis (and intends to pay the relevant APC), the terms of reuse of such Open Access shall be governed by a Creative Commons licence – details of these licences and which Creative Commons licence will apply to this Work are set out in our licence referred to above.

Other than as permitted in any relevant BMJ Author's Self Archiving Policies, I confirm this Work has not been accepted for publication elsewhere, is not being considered for publication elsewhere and does not duplicate material already published. I confirm all authors consent to publication of this Work and authorise the granting of this licence.

For peer review only

Hospital management of major stroke types in Chinese adults: a population-based study of 20,000 hospitalised stroke cases

Haiqiang Qin MD*[1], Iain Turnbull MRCP* [2], Yiping Chen DPhil* [2,3], Neil Wright PhD[2], Liping Liu MD[1], Pei Pei BSc[4], Wei Tang BSc[6], Shengping Xiang BSc[7], Yu Guo MSc[4] , Xingquan Zhao MD[1], Robert Clarke FRCP[2], Liming Li MPH[4,5], Yongjun Wang MD[1], Zhengming Chen DPhil[2] on behalf of the China Kadoorie Biobank Collaborative Group

Equal contribution

1. China National Clinical Research Centre for Neurological Diseases; Department of Neurology, Beijing Tiantan Hospital, Capital Medical University, Beijing, China
2. Clinical Trial Service Unit and Epidemiological Studies Unit (CTSU), Nuffield Department of Population Health, University of Oxford, Oxford, UK
3. Medical Research Council Population Health Research Unit (PHRU), Nuffield Department of Population Health, University of Oxford, Oxford, UK
4. Chinese Academy of Medical Sciences, Dong Cheng District, Beijing, China
5. Department of Epidemiology and Biostatistics, School of Public Health, Peking University Health Science Centre, Beijing, China
6. Emergency Department, Pengzhou Traditional Chinese Medical Hospital, Pengzhou, Sichuan, China
7. Pengzhou Tongyi Hospital, Pengzhou, Sichuan, China

Address for correspondence

Prof Robert Clarke
Clinical Trial Service Unit
NDPH
Big Data Institute Building
Old Road Campus
University of Oxford
E-mail: robert.clarke@ndph.ox.ac.uk

Dr. Yiping Chen
Clinical Trial Service Unit
NDPH
Big Data Institute Building
Old Road Campus
University of Oxford
E-mail: yiping.chen@ndph.ox.ac.uk

Word count: Abstract n=258; text n=3,133
3 Tables, 2 Figure 1 eTable and 4 eFigures

CKB Research Tracker no. 2018-0071

Key words: Ischaemic Stroke, hospital treatment, antiplatelet medication, blood pressure-lowering medication, lipid-lowering medication, traditional Chinese medicines

7 October 2021

For peer review only - http://bmjopen.bmj.com/site/about/guidelines.xhtml

Abstract

Background: Little is known about the use of hospital treatments for stroke types in Chinese adults. We assessed the hospital management of stroke by pathological type, sex, age, calendar year, hospital tier, region and other factors.

Methods: The China Kadoorie Biobank recruited >0.5 million adults during 2004-08 from ten diverse areas and medical records were retrieved from 20,229 participants (n=261 hospitals) hospitalised with a first-ever incident stroke during a median follow-up of 11 years.

Results: Among 20,299 first-ever stroke cases, 17,306 (85%) had ischaemic stroke (IS; 7,123 non-lacunar [non-LACI], 6,690 lacunar [LACI], 3,493 had silent lacunar infarction [silent LACI]), 2,623 (13%) had intracerebral haemorrhage (ICH), and 370 (2%) had subarachnoid haemorrhage (SAH). Among IS cases, antiplatelet treatment was used by 64% (65% non-LACI, 66% LACI, 56% silent LACI), lipid-lowering by 50% (52% non-LACI, 53% LACI, 43% silent LACI), and blood pressure-lowering by ~42% of all IS types, with positive trends in the use of these treatments by calendar year and hospital tier. Among ICH cases, 53% used blood pressure-lowering and 10% used lipid-lowering treatments, respectively. In contrast, traditional Chinese medicines (TCM) were used by 59% of IS (50% non-LACI, 62% LACI, 74% silent LACI), 38% of ICH, and 30% of SAH cases, with positive trends by calendar year and by hospital tier.

Conclusions: Among IS cases, use of antiplatelet and lipid-lowering medications increased in recent years, but use of TCM still exceeded use of blood pressure-lowering treatment. In contrast, blood pressure-lowering treatment was widely used for ICH, but only half of all ICH cases used blood pressure-lowering treatment.

For peer review only

For peer review only - http://bmjopen.bmj.com/site/about/guidelines.xhtml

Strengths and limitations of this study

- A nationwide community-based prospective cohort study involving over 0.5 million of men and women from 5 rural and 5 urban areas in China.
- During a median follow-up of 11 years, data were collected on 20,000 well-characterised stroke cases, which were classified by pathological types.
- The use of evidence-based treatments for stroke prevention has improved in recent years, but a high proportion of stroke cases are still treated with traditional Chinese medicines without evidence from large randomised clinical trials.
- The study was not representative of the Chinese population and data collection was limited to first stroke events.

For peer review only

Introduction

Stroke is a major cause of death and disability worldwide, with three-quarters (~6.3 million) of deaths each year occurring in developing countries including China.[1-3] In China, stroke is now the leading cause of death and disability, accounting for almost 2 million deaths in 2017.[3-5] While mortality rates from stroke have declined substantially over the last few decades in high-income countries, the annual costs for hospital treatment and rehabilitation of stroke were 40 billion Chinese Yuan (CNY) and approximately 6 billion US dollars (USD) in 2016, which were 10-fold higher than those for ischaemic heart disease (IHD).[3] The average per-patient cost of hospitalisation for stroke cases in China in 2018 was 14,291 Chinese Yuan (CNY) (9,409 CNY [1,309 USD] for ischaemic stroke and 19,149 CNY [2,772 USD] for haemorrhagic stroke).

In recent decades, evidence-based guidelines for treatment of different stroke pathological types have been developed, regularly updated, and widely disseminated in China. In contrast, whereas Traditional Chinese Medicines (TCM), for which no randomised evidence exists, continue to be extensively used for the treatment of hospitalised stroke cases.[6] Nevertheless, there is still substantial variation in access to and use of evidence-based treatments for stroke in different regions of China.[6] While such treatments have been extensively studied in Western countries,[7-9] little is known about the contemporary use of evidence-based medical and surgical treatments for hospitalised cases of stroke in China. Previous studies of hospital management of stroke in China have been constrained

For peer review only

For peer review only - http://bmjopen.bmj.com/site/about/guidelines.xhtml

by small sample sizes and involvement of only large teaching or tertiary hospitals, or restricted to studies in large urban areas only.[10-15]

We examined the use of medical and surgical treatments for first-ever incident stroke cases by pathological type, overall and by sex, age of onset, calendar year, hospital tier, region and other risk factors during an 11-year follow-up of 0.5 million adults in the China Kadoorie Biobank (CKB). [16,17]

Methods

Study population

Details of the study design and methods for recruitment and follow-up used in the CKB have been previously reported.[16,17] In brief, the 2004-8 baseline survey was conducted in 5 urban cities (Qingdao, Harbin, Liuzhou, Haikou, and Suzhou) and 5 rural counties (Henan, Hunan, Sichuan, Gansu, and Zhejiang) in China, selected to maximise regional differences in risk exposures and prevalence of major diseases. In each area, all permanent residents aged 35-74 years who were without disability were invited to participate. A total of 512,726 individuals (including some outside the target age range) agreed to participate in the study. The main analyses of the present report were restricted to adjudicated first-ever incident cases of stroke occurring prior to 31 December 2017 among 484,413 participants with no self-reported heart disease or stroke/transient ischaemic attack (TIA) at baseline (n=23,129) or stroke prior to their first adjudicated stroke (n=5,184).

For peer review only - http://bmjopen.bmj.com/site/about/guidelines.xhtml

Data on socio-demographic status, smoking and alcohol consumption, diet, physical activity and medical history were collected by trained health workers using laptop-based questionnaires at local study clinics. All participants also had clinical measurements recorded, including height, weight, waist and hip circumference, lung function, blood pressure and heart rate and a blood sample collected. Ethical approval for the China Kadoorie Biobank was obtained from the Oxford University Tropical Research Ethics Committee (OxTREC; [2005]; OxTREC Reference 025-04), and the Chinese Center for Disease Control and Prevention Ethical Review Committee (2004; Reference 005/2004). All participants provided written informed consent for participating and for accessing their medical records during follow-up by study investigators.

After the baseline survey, the vital status of participants was monitored regularly through Disease Surveillance Points (DSP) death registries, supplemented by annual checks with local residential records and active confirmation through local street committees or village administrators.[17] The occurrence of stroke among participants was identified by electronic linkage, using a unique personal identification number, to the national health insurance (HI) system that covered >97% of CKB participants, and to death and disease-specific (stroke, IHD, cancer and diabetes) registries accessible in the local study areas.

Selected medical records of hospitalised cases of first-ever reported stroke cases were retrieved and the diagnosis was verified by trained local CKB staff at each of the 261 different hospitals. The retrieved documents (e.g. initial covering page and discharge summary of the first admission record, with reports of brain imaging and use of pre-

For peer review only - http://bmjopen.bmj.com/site/about/guidelines.xhtml

specified medical treatments for stroke) were reviewed electronically by 26 accredited Chinese neurologists and stroke physicians from Tier III (top ranking) hospitals in China using bespoke IT systems. While indicators of medical treatments were pre-specified at three time periods (first 24 hours after admission, during hospital stay, and at discharge) in the medical notes, information on timing of such treatments was incomplete and consequently it was not possible to conduct separate analysis of use of drug treatment during each time period. Hence, recorded hospital treatment in any of the three periods was used in the present analysis.

Classification of stroke pathological types

All reported and adjudicated diagnoses of stroke pathological types were checked centrally by study staff and standardised by 10th Revision of the International Statistical Classification of Diseases and Related Health Problems (ICD-10) codes using bespoke software. The major stroke pathological types examined were ischaemic stroke (IS; ICD-10 I63, including lacunar infarction [LACI] and non-lacunar infarction [non-LACI]), intracerebral haemorrhage (ICH; I61), subarachnoid haemorrhage (SAH; I60), and unspecified stroke (I64). IS was defined as focal neurological dysfunction lasting for more than 24 hours with or without neuroimaging evidence of a cerebral infarct. LACI was classified by the evidence of an infarct <1.5 cm in diameter on CT (computed tomography) or MR (magnetic resonance) brain imaging with focal neurological deficits, including lacunar syndrome.[18] Silent lacunar infarct silent LACI) was characterised by imaging

For peer review only - http://bmjopen.bmj.com/site/about/guidelines.xhtml

evidence of a cerebral infarct ,1.5 cm in diameter in the absence of any focal neurological deficit. Non-LACI was defined as any IS type excluding LACI.

ICH was defined as a neurological dysfunction caused by haemorrhage into the brain parenchyma or the ventricular system, excluding those induced by injury, with or without neuroimaging evidence of haemorrhage. SAH was defined as a neurological dysfunction caused by a haemorrhage into the subarachnoid space, excluding those induced by injury, with or without relevant neuroimaging evidence. In addition, all silent LACI cases were reviewed centrally by two research clinicians with relevant training in neurology and included with other stroke cases in an "any stroke" endpoint.

Patient and public involvement

Prior to recruitment in the CKB, local community leaders in China were consulted. The CKB study findings[19] are routinely reported in peer-review publications and any relevant public health messages disseminated[20] using local press, television and internet.

Statistical analysis

The present study included a total of 20,299 first ever incident stroke cases with no prior history of a CVD event at baseline questionnaire or stroke event before the first adjudicated event. All cases were adjudicated online by senior neurologists in China and reviewed centrally by study clinicians in Oxford. The rates of incident stroke cases were standardised to the overall CKB population by sex, area, and age at diagnosis. Rates of

For peer review only

For peer review only - http://bmjopen.bmj.com/site/about/guidelines.xhtml

use of medical and surgical treatments were estimated separately for any stroke and for stroke pathological types, by sex, and area (rural vs urban), separately. All analyses were conducted using R 3.5.0.

Patient and public involvement

Patients and the public were not involved in the design of the study, or in the interpretation or writing up of the manuscript.

Results

Characteristics of cases with different stroke types

Among 484,413 participants included in the present analysis, the mean age (SD) at baseline was 51.5 (10.5) years, 59% were women, and 43% lived in urban areas (**Table 1**). During a median follow-up of 11 years (4.72 million person-years), a total of 61,902 incident stroke cases were reported, including 57,190 (92%) hospitalised cases. Overall, there were 20,299 first-ever hospitalised cases of primary stroke for which each diagnosis was confirmed by clinical adjudication, including 17,306 (85%) with IS (7,123 non-LACI, 6,690 LACI, and 3,493 silent LACI), 2,623 with ICH, and 370 with SAH, respectively. Overall, 93% of the reported stroke cases (92% for IS, 97% for ICH and 96% for SAH) had brain imaging.

Compared with those without stroke, individuals who experienced first-ever stroke during follow-up were older and more likely to be male, to reside in urban areas, and to have lower levels of education and household income at baseline (**Table 1**). Individuals with first-ever ICH were more likely than those with other stroke types to be men, to live in rural

For peer review only - http://bmjopen.bmj.com/site/about/guidelines.xhtml

areas, and to have lower levels of full-time education (54.8% had no formal/primary schooling: **Table 1**) and have low annual household income (30.6% had income less than 10,000 CNY: **Table 1**). Likewise, individuals with ICH had the highest proportion with self-reported hypertension (23.5%: **Table1**), and the highest mean levels of systolic blood pressure (SBP) (149 mmHg: **Table 1**). With the exception of LACI cases (who were more likely to be female and live in urban areas), individuals with non-LACI and LACI had broadly similar baseline characteristics (**Table 1**). In addition, the characteristics of individuals with LACI and silent LACI cases were comparable. However, compared with LACI, silent LACI cases had lower mean levels of SBP (133 mmHg vs 138 mmHg). The baseline characteristics of individuals with SAH were generally similar to those with ICH (**Table 1**). Interestingly, the overall prevalence of atrial fibrillation was very low, albeit higher in non-LACI than in LACI cases (0.3% vs 0.1%: **Table 2**).

Hospital treatment in IS cases

Overall, antiplatelet agents (aspirin or clopidogrel) were administered to 63.7% of all hospitalised IS cases, but use of antiplatelet agents was higher in cases with LACI (66.3%) and non-LACI (65.1%) than silent LACI cases (55.6%) (**Table 2**). Dual antiplatelet therapy was much less commonly used (non-LACI 8.3%, LACI 6.2%, and silent LACI 2.6%, respectively). Intravenous thrombolytic treatments (rtPA and urokinase) were used in 1.2% of non-LACI and 0.3% of LACI cases. Overall, 42% of all IS cases received blood pressure-lowering treatments, with similar proportions for different IS types (42.2%, 41.8%, and 40.8% for LACI, non-LACI, and silent LACI, respectively). Likewise, lipid-lowering

For peer review only - http://bmjopen.bmj.com/site/about/guidelines.xhtml

treatments were used by only about half of all hospitalised cases of IS (52.6%, 51.5%, and 43.2% for LACI, non-LACI, and silent LACI, respectively). In contrast, 56% of IS cases (62.0%, 49.9%, and 73.6%, for LACI, non-LACI, and silent LACI, respectively) received TCM (**Table 2**).

For antiplatelet and lipid-lowering medications in addition to TCM, the rates of use for IS increased with age, while no such increasing trends with age were observed for use of blood-pressure lowering treatments **(Figure 1)**. All medical treatments for IS, with the exception of blood pressure-lowering therapy, increased by calendar year of follow-up between 2005 and 2017 **(Figure 2)**. While the use of blood pressure-lowering treatments did not differ significantly by hospital tiers, the use of TCM was higher in Tier III hospitals and increased by calendar year between 2005 to 2017 (**Figure 2**). Similarly, age-specific use of antiplatelet, blood pressure-lowering, and lipid-lowering agents and TCM among IS cases did not vary appreciably by sex or area of residence (**eFigures 1-6**), but use of TCM increased by hospital tier in both men and women and in rural and urban areas. Carotid endarterectomy and stenting were very rarely undertaken (**Table 2**).

Hospital management of ICH and SAH

Among participants hospitalised with ICH, 3.9% had surgical clot evacuation. A small proportion of cases with SAH underwent endovascular coiling (7.0%) or neurosurgical clipping of an aneurysm (5.7%). Approximately half of ICH (52.6%) or SAH (50.5%) cases received blood pressure-lowering medication, which did not vary significantly by sex or

For peer review only - http://bmjopen.bmj.com/site/about/guidelines.xhtml

area (**eFigure 1**). Overall, the use of lipid-lowering agents was 10.4% for ICH whereas approximately one-third of ICH (37.8%) and SAH (29.7%) cases received TCM while in hospital (**Table 3**).

Discussion

The present study, involving over 20,000 incident stroke cases, demonstrated that while there have been substantial improvements in access to and use of brain imaging for diagnosis of stroke types in hospitalised cases, the use of many evidence-based medical treatments, including antiplatelet, blood pressure-lowering, and lipid-lowering agents, remains low in China, even in top-rank hospitals. In contrast, the use of TCM has remained consistently high, especially in higher-ranking hospitals, highlighting the urgent need for better utilisation of evidence-based treatments, particularly in IS cases. Likewise, further trials are required to examine the efficacy and safety of such treatments in ICH cases. Importantly, further assessments of the use of TCM in the management of both IS and ICH are also indicated.[19]

Despite marked improvements in the hospital management of stroke in recent decades in China,[15] the use of evidence-based medications remains lower than in Western countries. Thrombolytic therapy (e.g. r-tPA), which was approved by the State Food and Drug Administration of China in 2001, was administered to <1% of potentially eligible cases in the present study, compared with 3.0-8.5% reported in the USA [20] and 1.6% reported by the Chinese National Stroke Registry (CNSR) in 2011.[21]

For peer review only - http://bmjopen.bmj.com/site/about/guidelines.xhtml

A recent American Heart Association registry study conducted during the same calendar period reported a higher use of antiplatelet agents in the USA vs China (83.9% vs 64%, respectively).[22] Since data on common adverse events including bleeding were not recorded in the present study, the possibility that the lower use of antiplatelet therapy was clinically indicated in this population cannot be entirely excluded. Likewise, the use of blood pressure-lowering and lipid-lowering medications was also higher in the USA compared with China (76.7% vs 41.7%, and 77.6% vs 50.3%, respectively).[22] Moreover, the use of drug treatments for secondary prevention of stroke in the CKB was significantly lower than those reported in the CNSR.[15] Lowering blood pressure is the most important treatment to improve outcomes of cases with SAH and ICH.[23,24] However, in the present study, only about half of all ICH and SAH cases (and only 42% of IS cases) received blood pressure-lowering treatment. One explanation for the discrepant findings on rates of use of treatment in stroke cases in the CKB and the CNSR may be that the former study was conducted in 10 diverse regions (5 rural regions and 5 urban) with 21% of hospitals involving lower-grade (Tier 1) hospitals, whereas recruitment of stroke cases in the latter study was restricted to those living in urban regions and those in higher-tier hospitals. For the same reason, the use of intravenous thrombolytic therapy was <1% in the CKB vs 1.5% in CNSR. Likewise, the prevalence of reported atrial fibrillation was lower than previously reported estimates from population studies in China, and only 7% of stroke cases had Holter monitoring investigations. In addition, medication data in the present study were obtained from retrieved hospital admission records rather than at later periods

For peer review only

of follow-up. Although in-hospital treatment data are reliable, intended use of medication following discharge from hospital may be underestimated.

While there is substantial underuse of evidence-based medical and surgical treatments in hospitalised stroke cases, TCM are widely used for all stroke types in China, particularly in Tier 3 hospitals, despite absent randomised evidence to support their efficacy and safety.[19,25] The overall rates of use of evidence-based medications were also lower among younger stroke cases consistent with previous reports.[26] The economic and social effects of stroke and its long-term sequelae in younger cases are considerable and these inequalities should be a priority for improvement. The suboptimal use of medical treatments may partly explain the high recurrence rates of IS recently reported in this study population.[27]

Rates of medical therapy did not vary appreciably among ischaemic stroke types (non-LACI, LACI, and silent LACI). However, relevant clinical guidelines address only the management of symptomatic stroke cases,[27,28] since there have been no randomised controlled trials including participants with silent LACI. The high prevalence of silent LACI – and its strong association with subsequent risks of symptomatic stroke, [29-31] cognitive decline and dementia, [32-34] psychiatric disorders,[35] and increased mortality,[36] highlight the need for new trials to assess the efficacy and safety of medical treatments in such cases.

The use of drug treatments has increased considerably in recent years, particularly after introduction of universal health coverage in 2009, reflecting the effects of health policy. A

For peer review only - http://bmjopen.bmj.com/site/about/guidelines.xhtml

recent report of the temporal trends in hospital admissions for stroke in China demonstrated that admissions for first stroke increased by 3-6% per year after 2009 following adjustment for age, other risk factors and incidence of other diseases.[37] These stroke admissions did not, however, vary appreciably between rural and urban areas or between hospital tiers, suggesting that low socio-economic status, which is more common in rural areas, is unlikely to explain the suboptimal use of evidence-based treatments for stroke in China.

Further studies on the adverse effects of medications (e.g., bleeding risks with antiplatelet agents or rhabdomyolysis in high-dose statin users) are also needed to improve our understanding of the reduced rates of use of evidence-based therapies for IS in Chinese populations. The consequences of their suboptimal use – and the relatively prevalent use of TCM – on short-term mortality and stroke recurrence following stroke also warrant further study.

The present study had several strengths, including the use of bespoke web-based adjudication systems to determine the diagnosis and clinical characteristics of over 20,000 reported stroke cases of all pathological types including silent LACI. Moreover, it included data from Tier I hospitals in rural areas that have not been included in previous studies. However, there were several limitations. Firstly, the CKB study was not a nationally representative study and, hence, the results cannot be representative of all areas in China. Secondly, it is possible that some selected clinical information relating to the acute phase of stroke (for example, administration of thrombolysis or aspirin treatment within 48 hours

For peer review only

of hospital admission) was recorded in the emergency room and omitted in the retrieved medical records. Not all hospitals recorded medications for intended treatment after discharge in the medical records, which may have led to underestimates of their actual use. Lastly, the present study did not collect records of side effects of treatments (e.g., bleeding associated with the use of antiplatelet agents). Consequently, the relatively low observed rates of use of medical treatment may reflect clinical discretion or caution. However, the possibility of missing clinical data was minimised by photographing all available relevant medical records and enlistment of specialist neurologists and stroke physicians from Tier III hospitals to adjudicate the diagnoses of stroke types.

Overall, this contemporary study of 20,000 hospitalised stroke cases demonstrated that use of all evidence-based medical treatments has increased substantially after the introduction of the nationwide universal healthcare system in China. Despite recent improvements in stroke management, there is still considerable underuse of many evidence-based treatments and widespread overuse of TCM for stroke in the absence of evidence on their efficacy and safety. Future studies should investigate the causes and consequences of variations in stroke care in clinical practice in China.[38] Moreover, further large randomised trials are needed to evaluate the efficacy and safety of established evidence-based treatments for silent LACI.

For peer review only - http://bmjopen.bmj.com/site/about/guidelines.xhtml

Disclosures: None of the authors have any disclosures that were relevant to this report.

Contributorship

HQ, IT, YC and RC designed the study. NW concluded the statistical analyses. HQ wrote the first draft and IT, YC and RC revised the report, tables and figures. PP, WT, SX, YG and XZ supervised the field work for data collection on drug treatment and ascertainment of outcomes. L Liu and YW provided critical advice on the analyses and interpretation and YG, LL and ZC supervised the overall conduct of the CKB study. All authors provided critical comments on the final report. RC and YC took responsibility for submission of the final version for publication.

Competing interests

None of the authors had any conflicts of interest in relation to this report.

Funding: The CKB baseline survey and the first re-survey were supported by the Kadoorie Charitable Foundation in Hong Kong. The long-term follow-up has been supported by Wellcome grants to Oxford University (212946/Z/18/Z, 202922/Z/16/Z, 104085/Z/14/Z, 088158/Z/09/Z) and grants from the National Key Research and Development Program of China (2016YFC0900500, 2016YFC0900501, 2016YFC0900504, 2016YFC1303904) and from the National Natural Silent LACIence Foundation of China (91843302). The UK Medical Research Council (MC_UU_00017/1,MC_UU_12026/2 MC_U137686851), Cancer Research UK (C16077/A29186; C500/A16896) and the British Heart Foundation (CH/1996001/9454), provide core funding to the Clinical Trial Service Unit and Epidemiological Studies Unit at Oxford University for the project.

For peer review only

For peer review only - http://bmjopen.bmj.com/site/about/guidelines.xhtml

Data availability statement

The China Kadoorie Biobank (CKB) is a global resource for the investigation of lifestyle, environmental, biochemical and genetic factors as determinants of common diseases. The CKB study group is committed to making the cohort data available to the scientific community in China, the UK and worldwide to advance knowledge about the causes, prevention and treatment of disease. Detailed information on data that are currently available for open access users and how to apply for these are provided on the study website (http://www.ckbiobank.org/site/Data+Access).

Researchers who are interested in obtaining the individual data from the China Kadoorie Biobank study that underlines this paper should contact ckbaccess@ndph.ox.ac.uk. A research proposal will be requested to ensure that analyses will be performed by bona fide researchers.

For peer review only

Figure Legends

Figure 1: Proportions of medical treatments in hospital by IS subtypes

Figure 2: Use of antiplatelet, antihypertensive, lipid−lowering and TCM treatments for ischaemic stroke by hospital tiers and calendar periods from 2005 to 2017

For peer review only

For peer review only - http://bmjopen.bmj.com/site/about/guidelines.xhtml

Table 1: Selected baseline characteristics of participants by types and subtypes of first adjudicated stroke

	IS			ICH	SAH	Any stroke	All CKB
	Non-LACI	LACI	Silent LACI				
	(n = 7,123)	(n = 6,690)	(n = 3,493)	(n = 2,623)	(n = 370)	(n = 20,299)	(n = 484,413)
**Age, %**							
30 – 39	2.0	1.8	2.0	4.4	6.3	2.4	16.0
40 – 49	13.1	14.3	13.4	16.9	21.4	14.5	31.0
50 – 59	32.5	34.3	33.0	34.6	36.6	33.8	30.8
60 – 69	35.5	34.4	35.1	30.0	24.8	33.9	16.6
70 – 79	16.9	15.2	16.4	14.2	11.0	15.5	5.7
Mean (SD)	60.1 (9.6)	59.6 (9.5)	60.0 (9.6)	58.3 (10.0)	56.5 (10.2)	59.5 (9.7)	51.5 (10.5)
**Sex, %**							
Male	50.0	46.5	39.0	53.1	36.9	47.4	40.8
Female	50.0	52.3	59.8	46.9	60.3	52.6	59.2
**Area, %**							
Rural	58.0	40.6	39.0	63.3	51.6	49.2	56.8
Urban	42.0	59.4	61.0	36.7	48.4	50.8	43.2
**Highest education, %**							
No formal / primary school	54.4	52.4	53.2	54.8	46.4	53.4	50.7
Middle / high school	40.1	42.4	38.8	40.3	40.1	42.3	43.6
College / University	4.0	4.1	4.8	4.1	5.6	4.3	5.7
**Annual household income (RMB), %**							
<9,999	30.1	27.3	29.6	30.6	29.6	29.2	28.3
10,000-19,999	29.4	33.5	30.1	30.6	24.0	30.7	28.8
20,000-34,999	22.9	21.0	21.4	24.3	23.5	23.6	24.7
≥35000	16.0	17.0	15.9	13.7	15.1	16.5	18.1
**Current smoker, %**							

For peer review only

For peer review only - http://bmjopen.bmj.com/site/about/guidelines.xhtml

Males	69.4	67.1	55.1	62.5	52.1	66.5	62.0
Female	3.5	3.6	1.8	2.2	4.3	3.0	2.3
**Current alcohol drinker, %**							
Males	35.2	38.6	33.9	38.8	24.8	36.5	33.9
Female	2.5	1.4	2.0	3.7	1.3	2.5	2.1
**Regular dietary intake †, %**							
Meat	79.2	82.0	79.1	81.7	72.3	82.3	82.9
Fish	44.8	45.4	42.4	42.8	41.6	45.6	46.8
Dairy	16.8	18.6	19.5	16.2	19.1	18.3	19.7
Fruit	55.1	54.8	54.9	54.8	58.6	56.3	59.5
**Prevalent disease ‡, %**							
Diabetes	6.8	5.9	4.1	3.2	2.0	5.3	2.7
Hypertension	21.8	19.3	19.3	23.5	14.3	20.8	9.7
**Clinical measures, mean (SD)**							
SBP (mmHg)	140.3 (23.9)	138.4 (23.9)	132.8 (22.6)	149.0 (24.5)	124.7 (22.6)	142.1 (24.2)	130.4 (20.9)
BMI (kg/m^2)	24.3 (3.6)	24.0 (3.4)	23.3 (3.4)	24.0 (3.6)	21.7 (3.5)	24.4 (3.5)	23.6 (3.4)
RPG (mmol/L)	6.8 (3.5)	6.4 (3.3)	6.1 (2.9)	6.2 (2.9)	5.7 (3.2)	6.5 (3.2)	6.0 (2.3)
**Hospital tier, %**							
Tier 1 (162 hospitals) *	2.7	4.5	2.6	2.6	3.4	3.2	
Tier 2 (53 hospitals)	4.2	6.2	3.7	4.7	3.1	4.7	
Tier 3 (46 hospitals)	91.6	88.1	90.6	91.9	85.7	92.0	

Means and percentages are directly standardised to age, sex and study area structure of the CKB study population after excluding 23,129 participants with prior CHD or stroke/TIA and 5,184 participants with a reported stroke prior to first adjudicated stroke, as appropriate.

† 1-3 days per week or more often

‡ Self-reported at baseline survey

* includes 126 community health centres

Abbreviations: LACI: lacunar infarction, SD: standard deviation, SBP: systolic blood pressure, BMI: body mass index, RPG: random plasma glucose

For peer review only

Table 2: Hospital managements for subtypes of IS

Treatment, n (%)	Non-LACI (n = 7,123)		LACI (n = 6,690)		Silent LACI (n = 3,492)		All IS (n = 17,305)		P
**Any antiplatelet agent**	4,638	(65.1)	4,434	(66.3)	1,943	(55.6)	11,015	(63.7)	<0.001
Aspirin	3,829	(53.8)	3,722	(55.6)	1,761	(50.4)	9,312	(53.8)	<0.001
Clopidogrel	456	(6.4)	431	(6.4)	132	(3.8)	1,019	(5.9)	<0.001
Dual antiplatelet therapy	589	(8.3)	413	(6.2)	90	(2.6)	1,092	(6.3)	<0.001
**Anticoagulant agent**	689	(9.7)	569	(8.5)	146	(4.2)	1,404	(8.1)	<0.001
**Any antihypertensive agent**	2,976	(41.8)	2,820	(42.2)	1,426	(40.8)	7,222	(41.7)	0.439
1 Antihypertensive agent	2,217	(31.1)	2,137	(31.9)	1,046	(30.0)	5,400	(31.2)	0.119
2 Antihypertensive agent	746	(10.5)	668	(10.0)	354	(10.1)	1,768	(10.2)	0.629
≥3 Antihypertensive agent	170	(2.4)	156	(2.3)	110	(3.2)	436	(2.5)	0.028
**Any lipid-lowering agent**	3,670	(51.5)	3,522	(52.6)	1,508	(43.2)	8,700	(50.3)	<0.001
Statin	3,601	(50.6)	3,441	(51.4)	1,461	(41.8)	8,503	(49.1)	<0.001
Other lipid lowering agent	133	(1.9)	130	(1.9)	63	(1.8)	326	(1.9)	0.879
**Thrombolytic Therapy**									<0.001
r-tPA	25	(0.4)	5	(0.1)	0	(0.0)	30	(0.2)	<0.001
Urokinase	60	(0.8)	15	(0.2)	0	(0.0)	75	(0.4)	<0.001
**Defibrase**	192	(2.7)	179	(2.7)	43	(1.2)	414	(2.4)	<0.001
**Any TCM**	3,545	(49.8)	4,151	(62.0)	2,571	(73.6)	10,267	(59.3)	0.095
**Atrial fibrillation**	24	(0.3)	8	(0.1)	4	(0.1)	36	(0.2)	<0.001

For peer review only

For peer review only - http://bmjopen.bmj.com/site/about/guidelines.xhtml

Table 3: Hospital management for ICH and SAH

Hospital management, n (%)	ICH (n = 2,623)		SAH (n = 370)	
**Brain Imaging (CT or MRI)**		(97.0)		(96.0)
**Any antihypertensive agent**	1,379	(52.6)	187	(50.5)
1 Antihypertensive agent	899	(34.3)	146	(39.5)
2 Antihypertensive agent	436	(16.6)	42	(11.4)
≥3 Antihypertensive agent	151	(5.8)	8	(2.2)
**Any lipid-lowering agent**	272	(10.4)	12	(3.2)
Statin	266	(10.1)	12	(3.2)
Other lipid lowering agent	8	(0.3)	0	(0.0)
**Any TCM**	992	(37.8)	110	(29.7)
**Surgery**	230	(8.8)	47	(12.7)
Clot evacuation	103	(3.9)	3	(0.8)
Coiling	0	(0.0)	26	(7.0)
Aneurysm clipping surgery	0	(0.0)	21	(5.7)

For peer review only

References

1. Feigin VL, Roth GA, Naghavi M, et al. Global burden of stroke and risk factors in 188 countries, during 1990-2013: a systematic analysis for the Global Burden of Disease Study 2013. Lancet Neurol 2016; **15**(9): 913-24.
2. Feigin VL, Krishnamurthi RV, Parmar P, et al. Update on the Global Burden of Ischemic and Hemorrhagic Stroke in 1990-2013: The GBD 2013 Study. Neuroepidemiology 2015; **45**(3): 161-76.
3. Chen WW, Gao RL, Liu LS, et al Summary of China Cardiovascular Diseases Report. *Chinese Circulation Journal;* **32** : 521-530
4. Institute for Health Metrics and Evaluation (IHME). GBD Compare. Seattle, WA: IHME,University of Washington, 2015. Available from http://vizhub.healthdata.org/gbd-compare. (accessed February 18, 2020).
5. China National Health Commission. China Health and Family Planning Statistical Yearbook 2019. *Peking Union Medical College Press* 2019.
6. Wu S, Wu B, Liu M, et al. Stroke in China: advances and challenges in epidemiology, prevention, and management. Lancet Neurol 2019; **18**(4): 394-405.
7. Bangalore S, Schwamm L, Smith EE, et al. Secondary Prevention after Ischemic Stroke or Transient Ischemic Attack. The American journal of medicine 2014.
8. Fonarow GC, Reeves MJ, Smith EE, et al. Characteristics, performance measures, and in-hospital outcomes of the first one million stroke and transient ischemic attack admissions in get with the guidelines-stroke. Circ Cardiovasc Qual Outcomes 2010; **3**(3): 291-302.
9. Feigin VL, Lawes CM, Bennett DA, Anderson CS. Stroke epidemiology: a review of population-based studies of incidence, prevalence, and case-fatality in the late 20th century. Lancet neurology 2003; **2**(1): 43-53.
10. Wang Y, Li Z, Zhao X, et al. Effect of a Multifaceted Quality Improvement Intervention on Hospital Personnel Adherence to Performance Measures in Patients With Acute Ischemic Stroke in China: A Randomized Clinical Trial. JAMA 2018; **320**(3): 245-54.
11. Heeley E, Anderson CS, Huang Y, et al. Role of health insurance in averting economic hardship in families after acute stroke in China. Stroke; a journal of cerebral circulation 2009; **40**(6): 2149-56.
12. Ding D, Lu CZ, Fu JH, Hong Z. Predictors of vascular events after ischemic stroke: the China ischemic stroke registry study. Neuroepidemiology 2010; **34**(2): 110-6.
13. Liu X, Xu G, Wu W, Zhang R, Yin Q, Zhu W. Subtypes and one-year survival of first-ever stroke in Chinese patients: The Nanjing Stroke Registry. Cerebrovasc Dis 2006; **22**(2-3): 130-6.
14. He Q, Wu C, Luo H, et al. Trends in In-Hospital Mortality among Patients with Stroke in China. PloS one 2014; **9**(3): e92763.
15. Li Z, Wang C, Zhao X, et al. Substantial Progress Yet Significant Opportunity for Improvement in Stroke Care in China. Stroke 2016; **47**(11): 2843-9.
16. Chen Z, Lee L, Chen J, et al. Cohort profile: the Kadoorie Study of Chronic Disease in China (KSCDC). Int J Epidemiol 2005; **34**(6): 1243-9.

For peer review only - http://bmjopen.bmj.com/site/about/guidelines.xhtml

17. Chen Z, Chen J, Collins R, et al. China Kadoorie Biobank of 0.5 million people: survey methods, baseline characteristics and long-term follow-up. Int J Epidemiol 2011; **40**(6): 1652-66.
18. Asdaghi N, Jeerakathil T, Hameed B, et al. Oxfordshire community stroke project classification poorly differentiates small cortical and subcortical infarcts. Stroke 2011; **42**(8): 2143-8.
19. Chen Z, Sandercock P, Xie JX, Peto R, Collins R, Liu LS. Hospital management of acute ischemic stroke in China. Journal of stroke and cerebrovascular diseases : the official journal of National Stroke Association 1997; **6**(5): 361-7.
20. Reeves MJ, Arora S, Broderick JP, et al. Acute stroke care in the US: results from 4 pilot prototypes of the Paul Coverdell National Acute Stroke Registry. Stroke; a journal of cerebral circulation 2005; **36**(6): 1232-40.
21. Wang Y, Liao X, Zhao X, et al. Using recombinant tissue plasminogen activator to treat acute ischemic stroke in China: analysis of the results from the Chinese National Stroke Registry (CNSR). Stroke 2011; **42**(6): 1658-64.
22. Bushnell CD, Olson DM, Zhao X, et al. Secondary preventive medication persistence and adherence 1 year after stroke. Neurology 2011; **77**(12): 1182-90.
23. Connolly ES, Jr., Rabinstein AA, Carhuapoma JR, et al. Guidelines for the management of aneurysmal subarachnoid hemorrhage: a guideline for healthcare professionals from the American Heart Association/american Stroke Association. Stroke 2012; **43**(6): 1711-37.
24. Hemphill JC, 3rd, Greenberg SM, Anderson CS, et al. Guidelines for the Management of Spontaneous Intracerebral Hemorrhage: A Guideline for Healthcare Professionals From the American Heart Association/American Stroke Association. Stroke 2015; **46**(7): 2032-60.
25. Wei JW, Huang Y, Wang JG, et al. Current management of intracerebral hemorrhage in China: a national, multi-centre, hospital register study. BMC Neurol 2011; **11**: 16.
26. Sappok T, Faulstich A, Stuckert E, Kruck H, Marx P, Koennecke HC. Compliance with secondary prevention of ischemic stroke: a prospective evaluation. Stroke 2001; **32**(8): 1884-9.
27. Kernan WN, Ovbiagele B, Black HR, et al. Guidelines for the prevention of stroke in patients with stroke and transient ischemic attack: a guideline for healthcare professionals from the American Heart Association/American Stroke Association. Stroke 2014; **45**(7): 2160-236.
28. Smith EE, Saposnik G, Biessels GJ, et al. Prevention of Stroke in Patients With Silent Cerebrovascular Disease: A Silent LACIentific Statement for Healthcare Professionals From the American Heart Association/American Stroke Association. Stroke 2017; **48**(2): e44-e71.
29. Fanning JP, Wong AA, Fraser JF. The epidemiology of silent brain infarction: a systematic review of population-based cohorts. BMC Med. 2014;12:119.
30. Gupta A, Giambrone AE, Gialdini G, Finn C, Delgado D, Gutierrez J, et al. Silent Brain Infarction and Risk of Future Stroke: A Systematic Review and Meta-Analysis. Stroke. 2016;47(3):719-25.
31. Vermeer SE, Hollander M, van Dijk EJ, Hofman A, Koudstaal PJ, Breteler MM. Silent brain infarcts and white matter lesions increase stroke risk in the general population: the Rotterdam Scan Study. Stroke. 2003;34(5):1126-9.
32. Vermeer SE, Prins ND, den Heijer T, Hofman A, Koudstaal PJ, Breteler MM. Silent brain infarcts and the risk of dementia and cognitive decline. N Engl J Med. 2003;348(13):1215-22.

For peer review only

33. Bos D, Wolters FJ, Darweesh SKL, Vernooij MW, de Wolf F, Ikram MA, et al. Cerebral small vessel disease and the risk of dementia: A systematic review and meta-analysis of population-based evidence. Alzheimers Dement. 2018;14(11):1482-92.
34. Smith EE, O'Donnell M, Dagenais G, Lear SA, Wielgosz A, Sharma M, et al. Early cerebral small vessel disease and brain volume, cognition, and gait. Ann Neurol. 2015;77(2):251-61.
35. van Agtmaal MJM, Houben A, Pouwer F, Stehouwer CDA, Schram MT. Association of Microvascular Dysfunction With Late-Life Depression: A Systematic Review and Meta-analysis. JAMA Psychiatry. 2017;74(7):729-39.
36. Bokura H, Kobayashi S, Yamaguchi S, Iijima K, Nagai A, Toyoda G, et al. Silent brain infarction and subcortical white matter lesions increase the risk of stroke and mortality: a prospective cohort study. J Stroke Cerebrovasc Dis. 2006;15(2):57-63.
37. Chen Y, Wright N, Guo Y, et al Mortality and recurrent vascular events after first incident stroke: a 9-year community-based study of 0·5 million Chinese adults. *Lancet Global Health* 2020, 8(4):e580-90
38. Levy M, Chen Y, Clarke R et al Socioeconomic differences in health-care use and outcomes for stroke and ischemic heart disease in China during 2009–16: a prospective cohort study of 0·5 million adults*; Lancet Global Health* 2020; 8(4): e591-e602

For peer review only

For peer review only - http://bmjopen.bmj.com/site/about/guidelines.xhtml

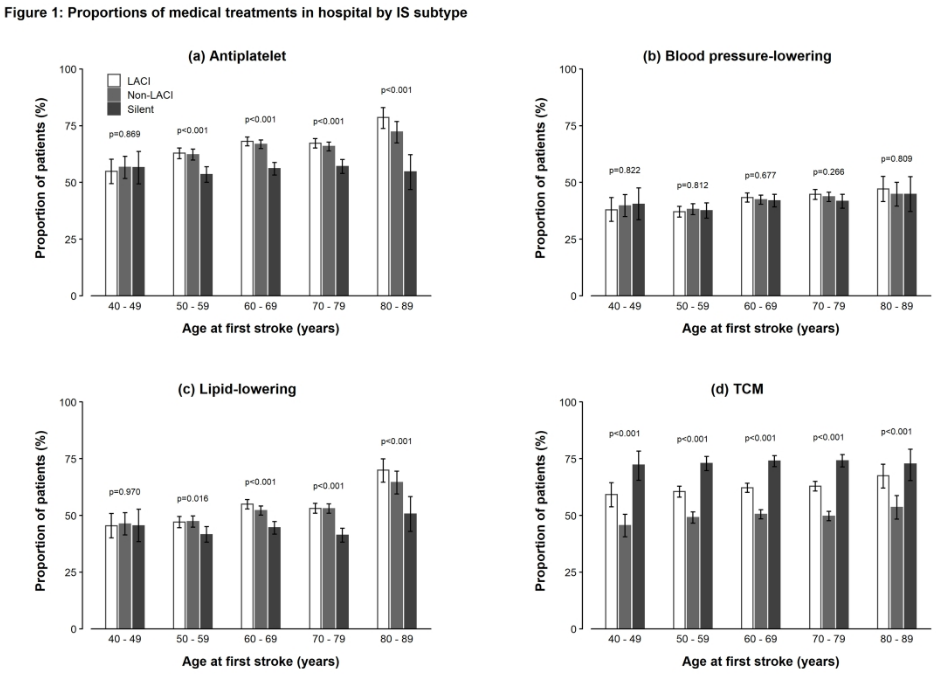

Figure 1: Proportions of medical treatments in hospital by IS type

296x209mm (300 x 300 DPI)

Figure 2: Antiplatelet, antihypertensive, lipid-lowering and TCM treatments for ischaemic stroke by hospital tiers and calendar periods from 2005 to 2017

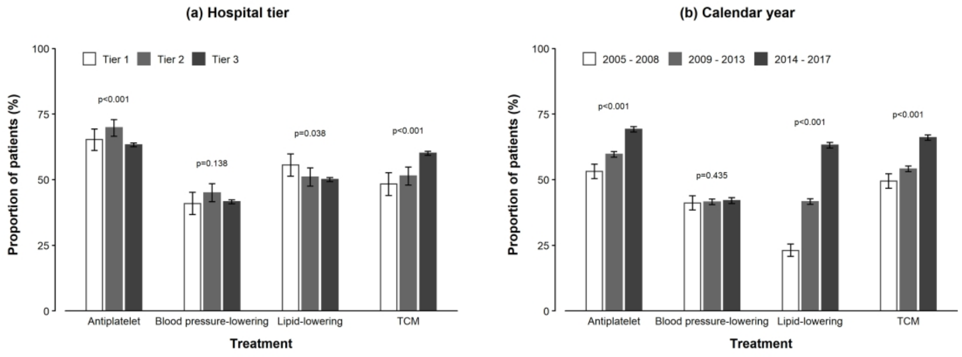

Figure 2: Antiplatelet, antihypertensive, lipid-lowering and TCM treatments for ischaemic stroke by hospital tiers and calendar periods from 2005 to 2017

296x209mm (300 x 300 DPI)

Supplementary Material for: "Hospital management of major stroke types in Chinese adults: a population-based study of 20,000 hospitalised stroke cases"

Members of the China Kadoorie Biobank collaborative group

eFigure 1: Proportions of use of antiplatelet treatments in hospital by IS subtype, sex and area

eFigure 2: Proportions of use of blood pressure-lowering treatments in hospital by IS subtype, sex and area

eFigure 3: Proportions of use of lipid-lowering treatments in hospital by IS subtype, sex and area

eFigure 4: Proportions of use of TCM in hospital by IS subtype, sex and area

eFigure 5: Use of antiplatelet, antihypertensive, lipid-lowering and TCM treatments for ischaemic stroke by hospital tiers, sex and area

eFigure 6: Use of antiplatelet, antihypertensive, lipid-lowering and TCM treatments for ischaemic stroke by calendar periods from 2005 to 2017

For peer review only

Members of the China Kadoorie Biobank collaborative group:

International Steering Committee: Junshi Chen, Zhengming Chen (PI), Robert Clarke, Rory Collins, Yu Guo, Liming Li (PI), Chen Wang, Jun Lv, Richard Peto, Robin Walters.

International Co-ordinating Centre, Oxford: Daniel Avery, Fiona Bragg, Derrick Bennett, Ruth Boxall, Ka Hung Chan, Yumei Chang, Yiping Chen, Zhengming Chen, Johnathan Clarke; Robert Clarke, Huaidong Du, Zammy Fairhurst-Hunter, Hannah Fry, Wei Gan, Simon Gilbert, Alex Hacker, Parisa Hariri, Mike Hill, Michael Holmes, Pek Kei Im, Andri Iona, Maria Kakkoura, Christiana Kartsonaki, Rene Kerosi, Kuang Lin, Mohsen Mazidi, Iona Millwood, Qunhua Nie, Alfred Pozarickij, Paul Ryder, Sam Sansome, Dan Schmidt, Paul Sherliker, Rajani Sohoni, Becky Stevens, Iain Turnbull, Robin Walters, Lin Wang, Neil Wright, Ling Yang, Xiaoming Yang, Pang Yao.

National Co-ordinating Centre, Beijing: Yu Guo, Xiao Han, Can Hou, Chun Li, Chao Liu, Jun Lv, Pei Pei, Canqing Yu.

10 Regional Co-ordinating Centres: Guangxi Provincial CDC: Naying Chen, Duo Liu, Zhenzhu Tang. Liuzhou CDC: Ningyu Chen, Qilian Jiang, Jian Lan, Mingqiang Li, Yun Liu, Fanwen Meng, Jinhuai Meng, Rong Pan, Yulu Qin, Ping Wang, Sisi Wang, Liuping Wei, Liyuan Zhou. **Gansu** Provincial CDC: Caixia Dong, Pengfei Ge, Xiaolan Ren. Maiji CDC: Zhongxiao Li, Enke Mao, Tao Wang, Hui Zhang, Xi Zhang. **Hainan** Provincial CDC: Jinyan Chen, Ximin Hu, Xiaohuan Wang. Meilan CDC: Zhendong Guo, Huimei Li, Yilei Li, Min Weng, Shukuan Wu. **Heilongjiang** Provincial CDC: Shichun Yan, Mingyuan Zou, Xue Zhou. Nangang CDC: Ziyan Guo, Quan Kang, Yanjie Li, Bo Yu, Qinai Xu. **Henan** Provincial CDC: Liang Chang, Lei Fan, Shixian Feng, Ding Zhang, Gang Zhou. Huixian CDC: Yulian Gao, Tianyou He, Pan He, Chen Hu, Huarong Sun, Xukui Zhang. **Hunan** Provincial CDC: Biyun Chen, Zhongxi Fu, Yuelong Huang, Huilin Liu, Qiaohua Xu, Li Yin. Liuyang CDC: Huajun Long, Xin Xu, Hao Zhang, Libo Zhang. **Jiangsu** Provincial CDC: Jian Su, Ran Tao, Ming Wu, Jie Yang, Jinyi Zhou, Yonglin Zhou. Suzhou CDC: Yihe Hu, Yujie Hua, Jianrong Jin Fang Liu, Jingchao Liu, Yan Lu, Liangcai Ma, Aiyu Tang, Jun Zhang. **Qingdao** Qingdao CDC: Liang Cheng, Ranran Du, Ruqin Gao, Feifei Li, Shanpeng Li, Yongmei Liu, Feng Ning, Zengchang Pang, Xiaohui Sun, Xiaocao Tian, Shaojie Wang, Yaoming Zhai, Hua Zhang, Licang CDC: Wei Hou, Silu Lv, Junzheng Wang. **Sichuan** Provincial CDC: Xiaofang Chen, Xianping Wu, Ningmei Zhang, Weiwei Zhou. Pengzhou CDC: Xiaofang Chen, Jianguo Li, Jiaqiu Liu, Guojin Luo, Qiang Sun, Xunfu Zhong. **Zhejiang** Provincial CDC: Weiwei Gong, Ruying Hu, Hao Wang,Meng Wan, Min Yu. Tongxiang CDC: Lingli Chen, Qijun Gu, Dongxia Pan, Chunmei Wang, Kaixu Xie, Xiaoyi Zhang.

Event Adjudication Clinicians: Beijing Tiantan Hospital,Capital Medical University Shuya Li, Haiqiang Qin, Yongjun Wang, **Peking University People's Hospital** Qiling Chen, Jihua Wang, **The 1st Affiliated Hospital of Harbin Medical University** Xiaojia Sun, Lei Wang, Xun Wang, Liming Zhang, Shanshan Zhou, **The 2nd Affiliated Hospital of Harbin Medical University** Hongyuan Chen, Li Chen, Haiyan Gou, Weizhi Wang, Yanmei Zhu, Yulan Zhu, **The 2nd Hospital of Hebei Medical University** Ning Zhang, **Huashan Hospital** Xin Cheng, Qiang Dong, Yi Dong, Kun Fang, Yiting Mao, **Jinling Hospital** Yu An, Peiling Chen, Yinghua Chen, Zhihong Liu, Lihua Zhang **The People's Hospital of Liaoning Province** Xiaohong Chen, Naixin Jv, Xiaojiu Li, Liyang Liu, Yun Lu, Xiaona Xing, **Qingdao Fuwai Cardiovascular Hospital** Shihao You, **Shengjing Hospital of China Medical University** Xiaoli Cheng, Chaojun Gua, Jinping Jiang, Jingyi Liu, Shumei Ma, **Shenyang Military General Hospital** Xuefeng Yang, **The First People's Hospital of Shenyang** Xiaomo Du, Jian Xu, Xuecheng Yang, Xiaodi Zhao, **West China Hospital, Sichuan University** Zilong Hao, Ming Liu, Deren Wang, **The Second Affiliated Hospital of Suzhou University** Xiaoting Li, **Suzhou Kowloon Hospital Shanghai Jiao Tong University School of Medicine** Lili Hui, Zhanling Liao, Feng Liu, **Qingdao Fuwai Cardiovascular Hospital** Chunning Feng, Dejiang Ji, Fengxia Qu, Wenwen Yuan, **The First Affiliated Hospital of Zhengzhou University** Xin Fu, **Zhongshan Hospital,** Jing Ding, Peng Du, Lirong Jin, Yueshi Mao, Xin Wang.

eFigure 1: Proportions of use of antiplatelet treatments in hospital by IS subtype, sex and area

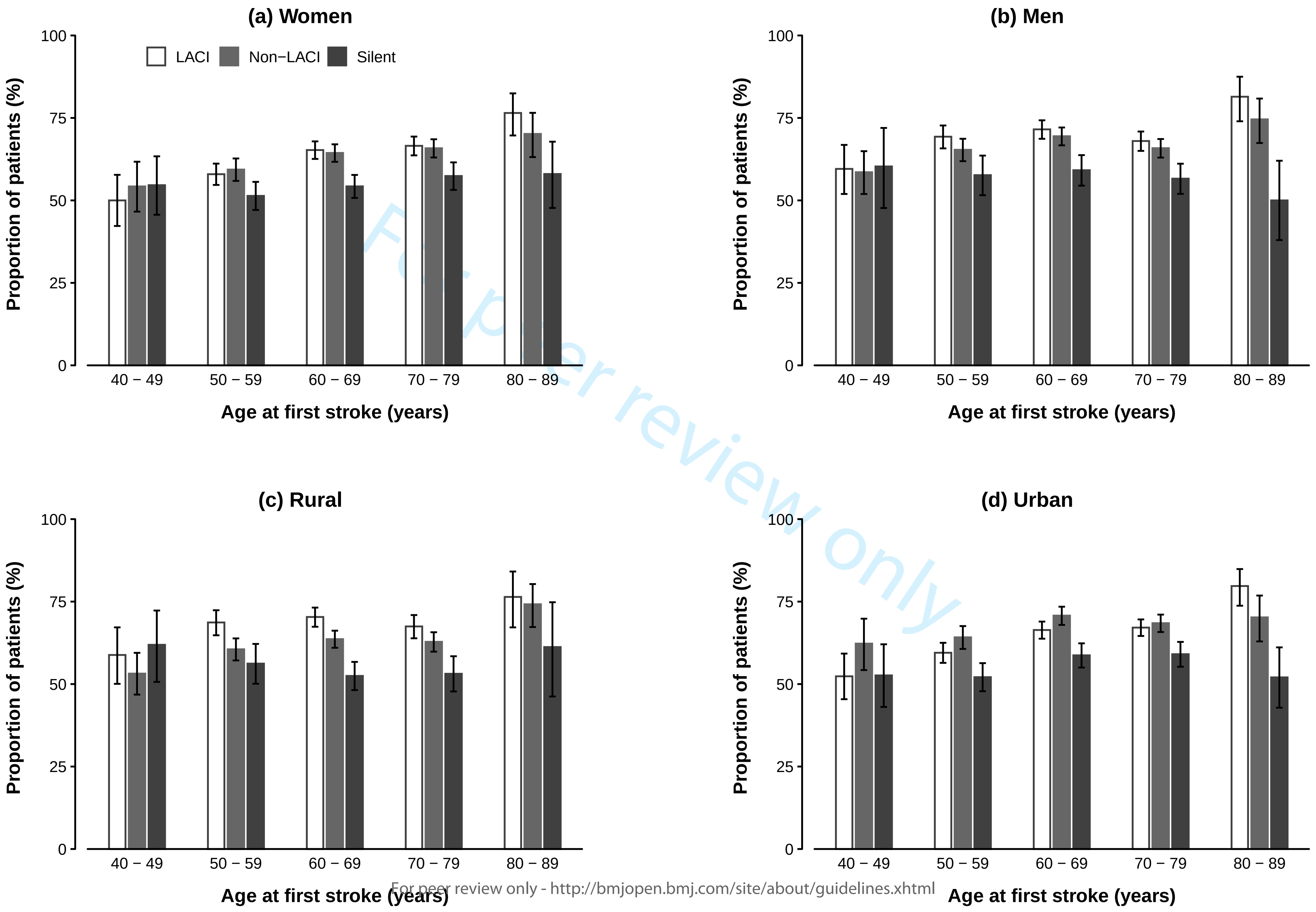

For peer review only

eFigure 2: Proportions of use of blood pressure−lowering treatments in hospital by IS subtype, sex and area

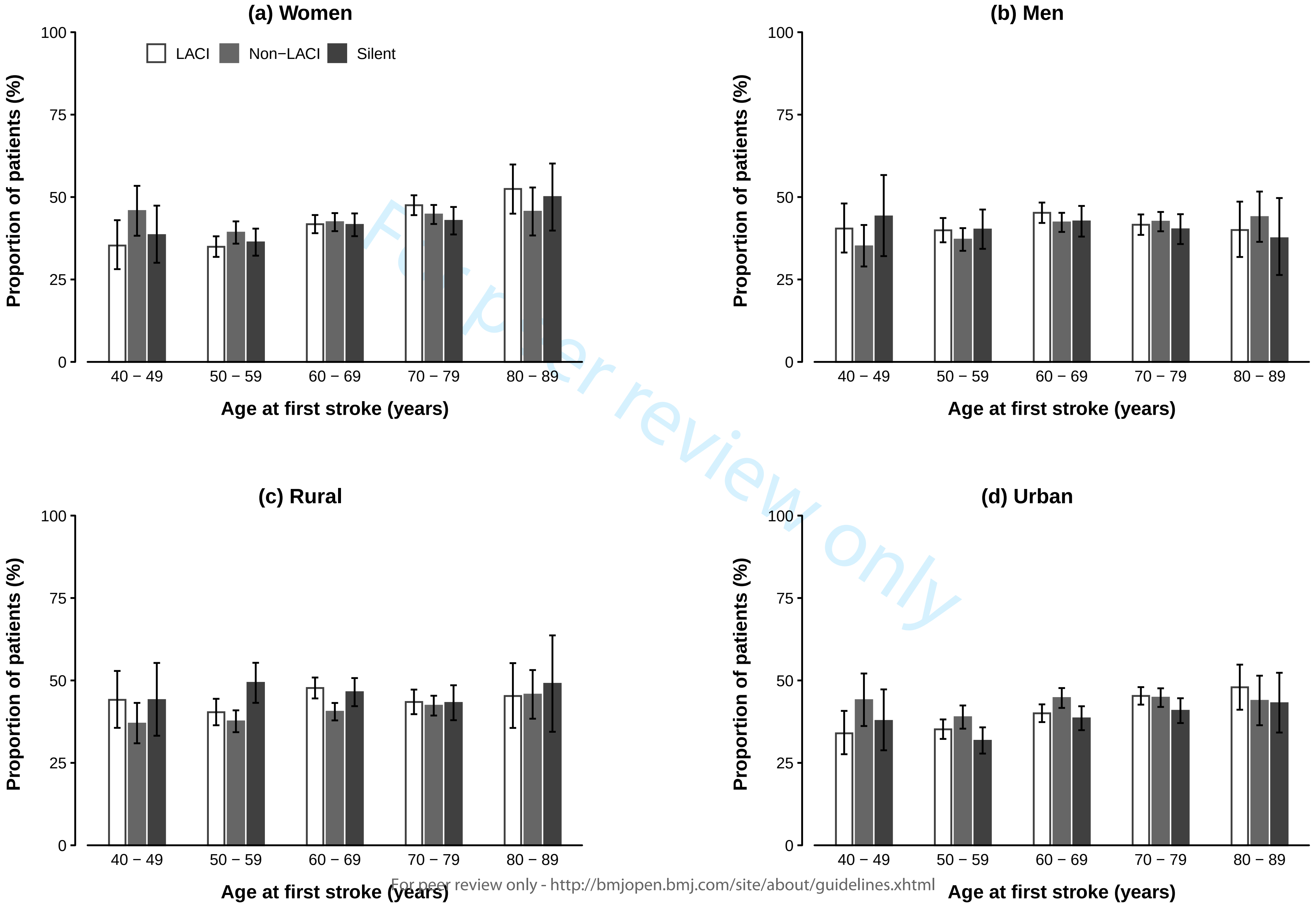

For peer review only

eFigure 3: Proportions of use of lipid−lowering treatments in hospital by IS subtype, sex and area

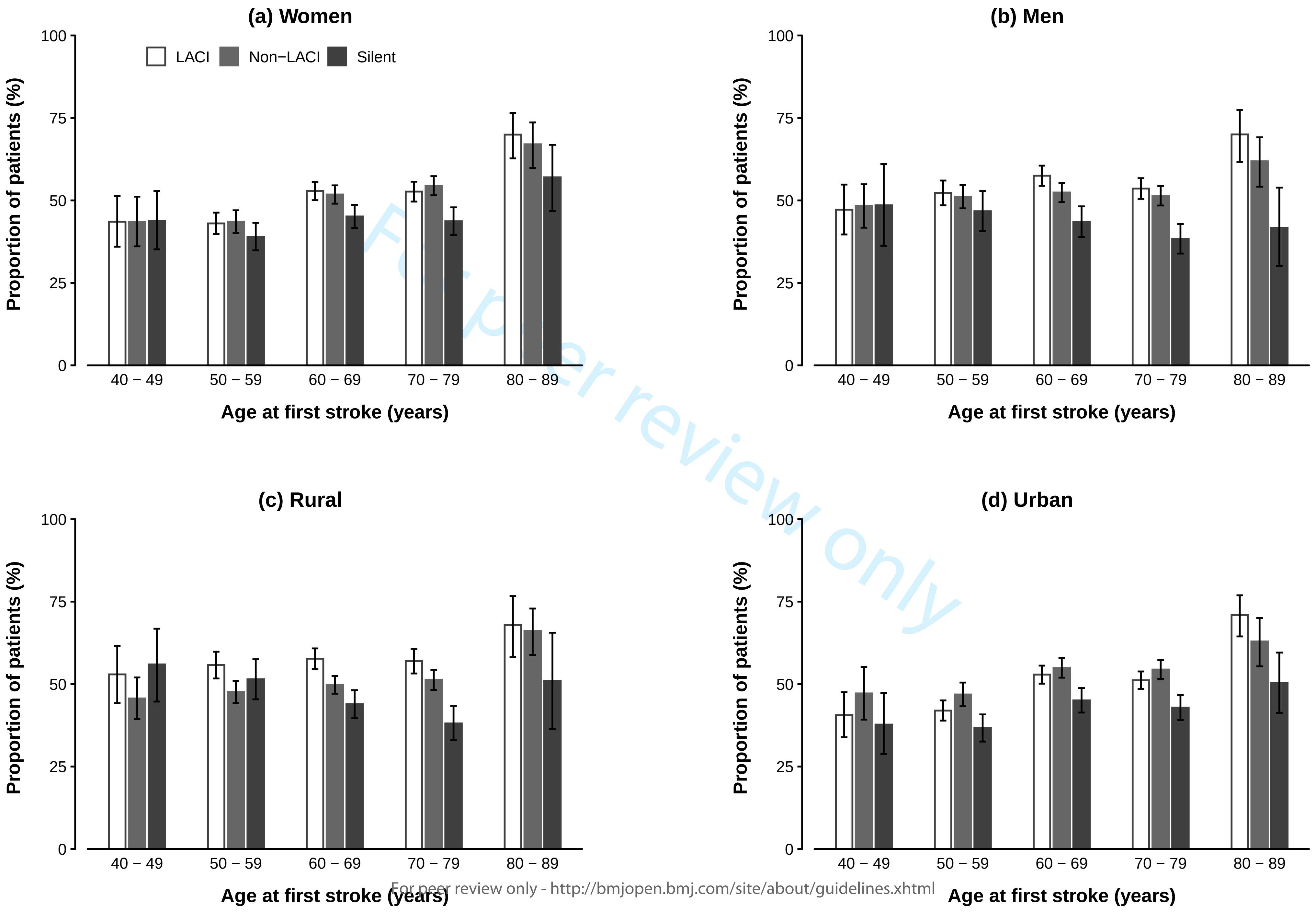

For peer review only

eFigure 4: Proportions of use of TCM in hospital by IS subtype, sex and area

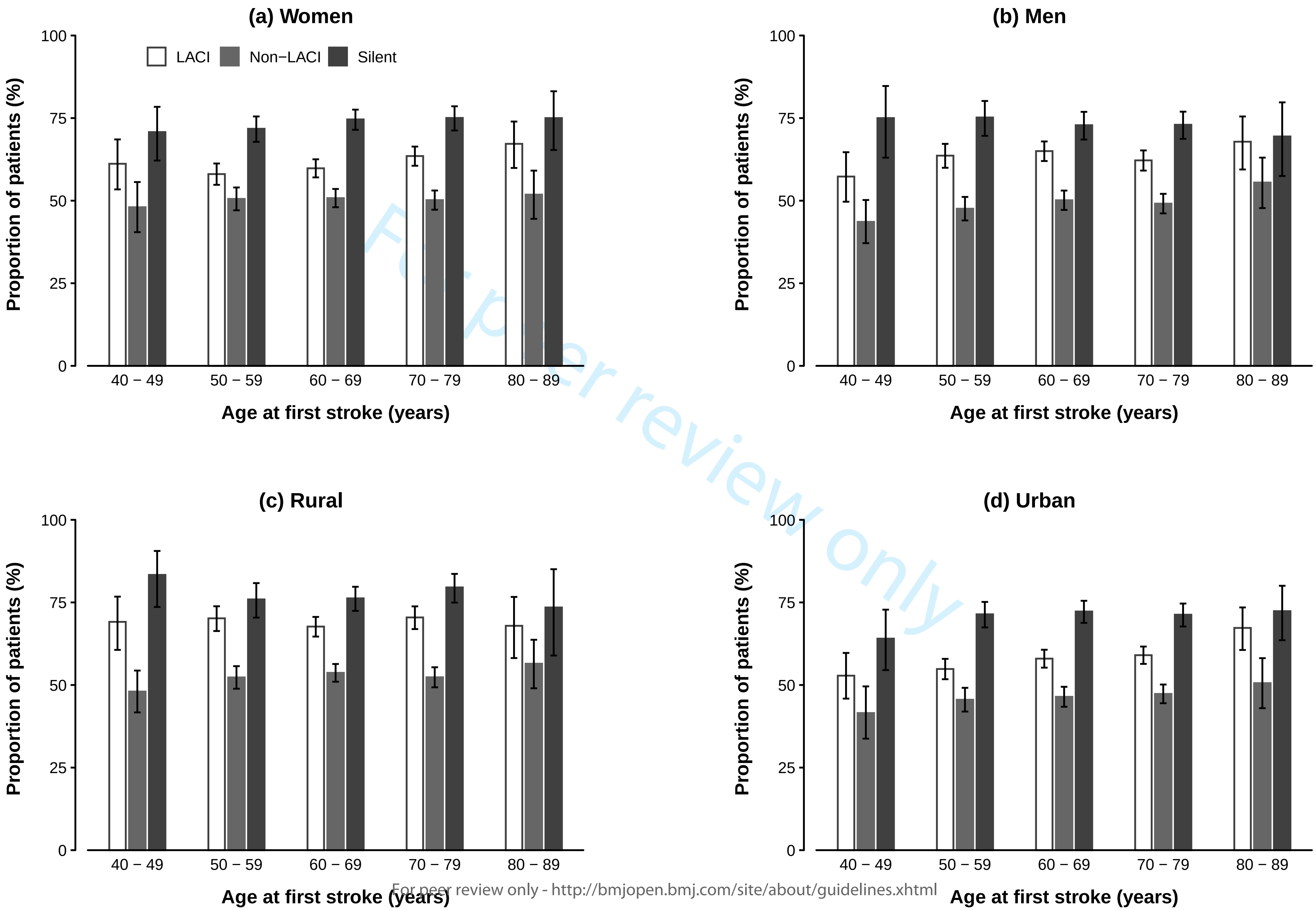

For peer review only

eFigure 5: Use of antiplatelet, antihypertensive, lipid−lowering and TCM treatments for ischaemic stroke by hospital tiers, sex and area

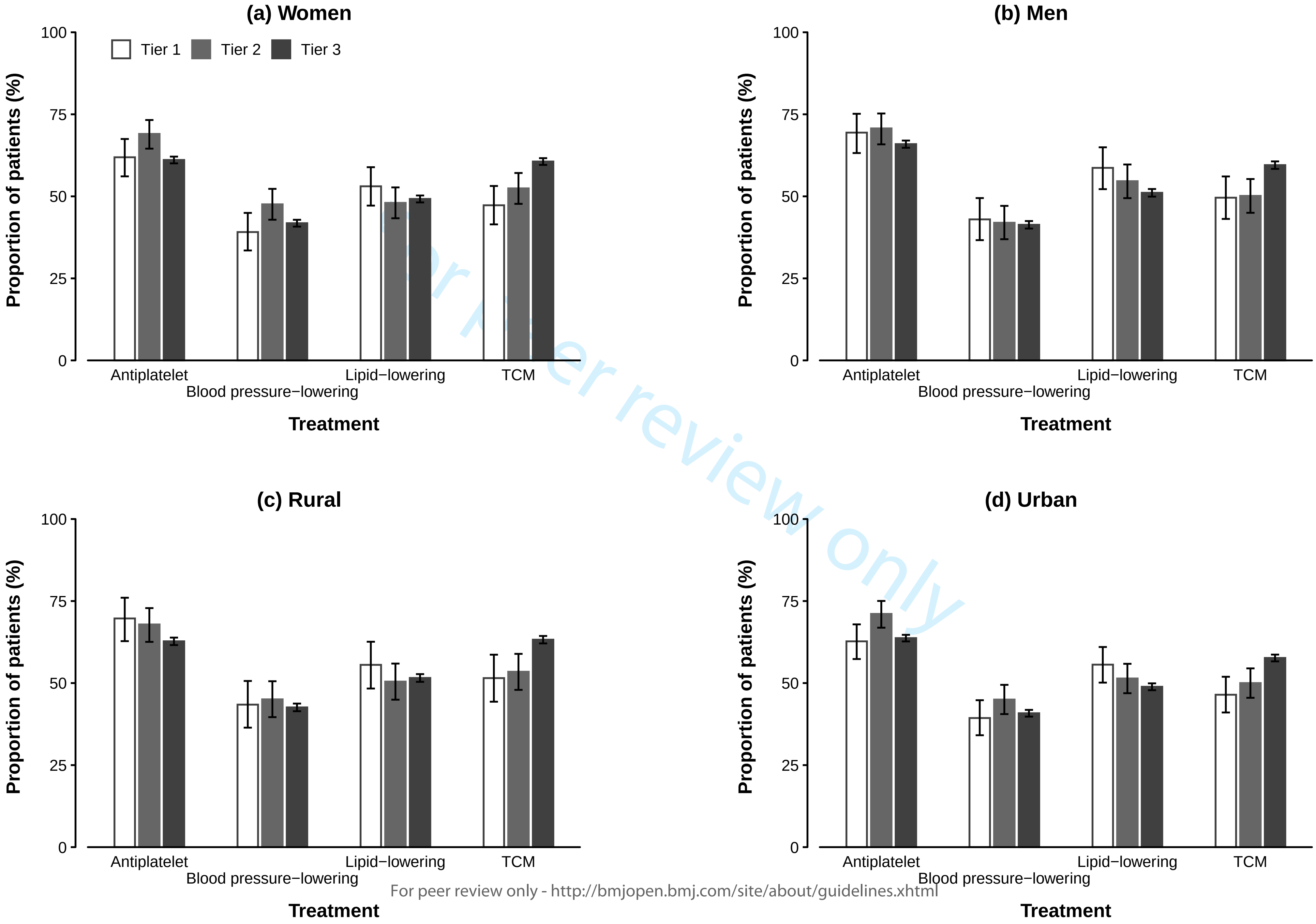

For peer review only

eFigure 6: Use of antiplatelet, antihypertensive, lipid−lowering and TCM treatments for ischaemic stroke by calendar periods from 2005 to 2017, sex and area

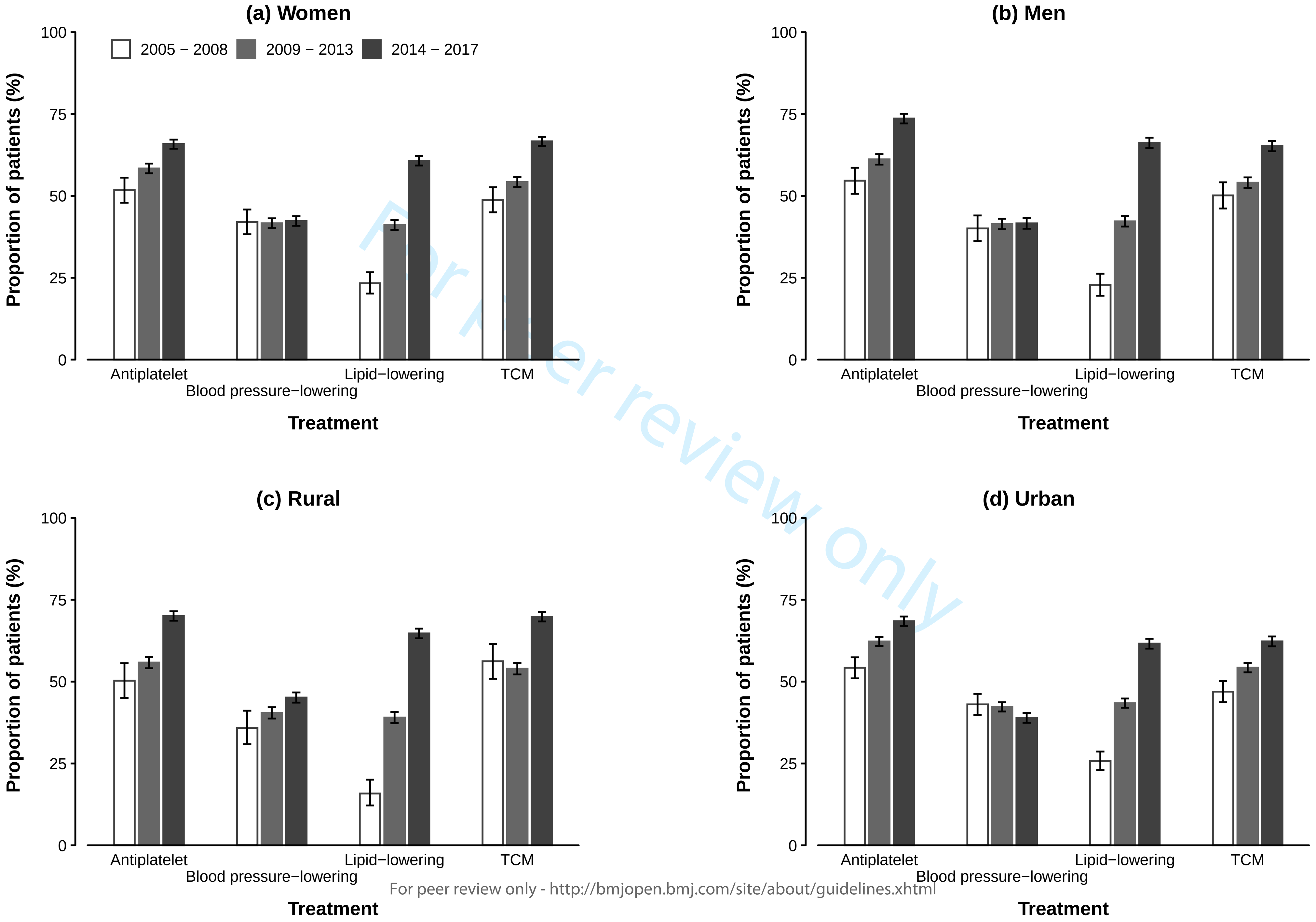

For peer review only

STROBE Statement—checklist of items that should be included in reports of observational studies

	Item No	Recommendation	Page No
**Title and abstract**	1	(*a*) Indicate the study's design with a commonly used term in the title or the abstract	1
		(*b*) Provide in the abstract an informative and balanced summary of what was done and what was found	2
**Introduction**			
Background/rationale	2	Explain the scientific background and rationale for the investigation being reported	3
Objectives	3	State specific objectives, including any prespecified hypotheses	3
**Methods**			
Study design	4	Present key elements of study design early in the paper	4
Setting	5	Describe the setting, locations, and relevant dates, including periods of recruitment, exposure, follow-up, and data collection	4
Participants	6	(*a*) *Cohort study*—Give the eligibility criteria, and the sources and methods of selection of participants. Describe methods of follow-up<br>*Case-control study*—Give the eligibility criteria, and the sources and methods of case ascertainment and control selection. Give the rationale for the choice of cases and controls<br>*Cross-sectional study*—Give the eligibility criteria, and the sources and methods of selection of participants	4-5
		(*b*) *Cohort study*—For matched studies, give matching criteria and number of exposed and unexposed<br>*Case-control study*—For matched studies, give matching criteria and the number of controls per case	
Variables	7	Clearly define all outcomes, exposures, predictors, potential confounders, and effect modifiers. Give diagnostic criteria, if applicable	5-6
Data sources/ measurement	8*	For each variable of interest, give sources of data and details of methods of assessment (measurement). Describe comparability of assessment methods if there is more than one group	*5-6*
Bias	9	Describe any efforts to address potential sources of bias	5
Study size	10	Explain how the study size was arrived at	4
Quantitative variables	11	Explain how quantitative variables were handled in the analyses. If applicable, describe which groupings were chosen and why	
Statistical methods	12	(*a*) Describe all statistical methods, including those used to control for confounding	7
		(*b*) Describe any methods used to examine subgroups and interactions	7
		(*c*) Explain how missing data were addressed	7
		(*d*) *Cohort study*—If applicable, explain how loss to follow-up was addressed<br>*Case-control study*—If applicable, explain how matching of cases and controls was addressed<br>*Cross-sectional study*—If applicable, describe analytical methods taking account of sampling strategy	7
		(*e*) Describe any sensitivity analyses	

Continued on next page

For peer review only - http://bmjopen.bmj.com/site/about/guidelines.xhtml

**Results**			
Participants	13*	(a) Report numbers of individuals at each stage of study—eg numbers potentially eligible, examined for eligibility, confirmed eligible, included in the study, completing follow-up, and analysed	7
		(b) Give reasons for non-participation at each stage	
		(c) Consider use of a flow diagram	
Descriptive data	14*	(a) Give characteristics of study participants (eg demographic, clinical, social) and information on exposures and potential confounders	7-8
		(b) Indicate number of participants with missing data for each variable of interest	
		(c) *Cohort study*—Summarise follow-up time (eg, average and total amount)	
Outcome data	15*	*Cohort study*—Report numbers of outcome events or summary measures over time	*8-10*
		Case-control study—Report numbers in each exposure category, or summary measures of exposure	
		Cross-sectional study—Report numbers of outcome events or summary measures	
Main results	16	(*a*) Give unadjusted estimates and, if applicable, confounder-adjusted estimates and their precision (eg, 95% confidence interval). Make clear which confounders were adjusted for and why they were included	8-10
		(*b*) Report category boundaries when continuous variables were categorized	
		(*c*) If relevant, consider translating estimates of relative risk into absolute risk for a meaningful time period	
Other analyses	17	Report other analyses done—eg analyses of subgroups and interactions, and sensitivity analyses	
**Discussion**			
Key results	18	Summarise key results with reference to study objectives	10-14
Limitations	19	Discuss limitations of the study, taking into account sources of potential bias or imprecision. Discuss both direction and magnitude of any potential bias	13-14
Interpretation	20	Give a cautious overall interpretation of results considering objectives, limitations, multiplicity of analyses, results from similar studies, and other relevant evidence	14
Generalisability	21	Discuss the generalisability (external validity) of the study results	14
**Other information**			
Funding	22	Give the source of funding and the role of the funders for the present study and, if applicable, for the original study on which the present article is based	Author statement

*Give information separately for cases and controls in case-control studies and, if applicable, for exposed and unexposed groups in cohort and cross-sectional studies.

Note: An Explanation and Elaboration article discusses each checklist item and gives methodological background and published examples of transparent reporting. The STROBE checklist is best used in conjunction with this article (freely available on the Web sites of PLoS Medicine at http://www.plosmedicine.org/, Annals of Internal Medicine at http://www.annals.org/, and Epidemiology at http://www.epidem.com/). Information on the STROBE Initiative is available at www.strobe-statement.org.

For peer review only

For peer review only - http://bmjopen.bmj.com/site/about/guidelines.xhtml

Hospital management of major stroke types in Chinese adults: a population-based study of 20,000 hospitalised stroke cases

Journal:	*BMJ Open*
Manuscript ID	bmjopen-2021-054265.R2
Article Type:	Original research
Date Submitted by the Author:	20-Oct-2021
Complete List of Authors:	Qin, Haiqiang; China National Clinical Research Centre for Neurological Diseases, Tiantian Hospital Beijing<br>Turnbull, Iain; University of Oxford, Clinical Trial Service Unit and Epidemiological Studies, NDPH<br>Chen, Yiping; University of Oxford, Clinical Trial Service Unit and Epidemiological Studies, NDPH<br>Wright, Neil ; University of Oxford, Clinical Trial Service Unit and Epidemiological Studies, NDPH<br>Liu, Liping; China National Clinical Research Centre for Neurological Diseases, Tiantian Hospital Beijing, Department of Neurology<br>Pei, Pei; Chinese Academy of Medical Sciences and Peking Union Medical College<br>Tang, Wei; Pengzhou Traditional Chinese Medical Hospital<br>Xiang, Shengping; Pengzhou Traditional Chinese Medical Hospital<br>Guo, Yu; Chinese Academy of Medical Sciences and Peking Union Medical College<br>Zhao, Xingquan; China National Clinical Research Centre for Neurological Diseases, Tiantian Hospital Beijing<br>Clarke, Robert; University of Oxford,<br>Li, Liming; Chinese Academy of Medical Sciences and Peking Union Medical College<br>Wang, Yongjun; China National Clinical Research Centre for Neurological Diseases, Tiantian Hospital Beijing<br>Chen, Zhengming; University of Oxford, Clinical Trial Service Unit and Epidemiological Studies, NDPH
**Primary Subject Heading**:	Epidemiology
Secondary Subject Heading:	Cardiovascular medicine
Keywords:	Neurology < INTERNAL MEDICINE, EPIDEMIOLOGY, Health policy < HEALTH SERVICES ADMINISTRATION & MANAGEMENT, STROKE MEDICINE, Herbal medicine < THERAPEUTICS

SCHOLARONE™
Manuscripts

I, the Submitting Author has the right to grant and does grant on behalf of all authors of the Work (as defined in the below author licence), an exclusive licence and/or a non-exclusive licence for contributions from authors who are: i) UK Crown employees; ii) where BMJ has agreed a CC-BY licence shall apply, and/or iii) in accordance with the terms applicable for US Federal Government officers or employees acting as part of their official duties; on a worldwide, perpetual, irrevocable, royalty-free basis to BMJ Publishing Group Ltd ("BMJ") its licensees and where the relevant Journal is co-owned by BMJ to the co-owners of the Journal, to publish the Work in this journal and any other BMJ products and to exploit all rights, as set out in our licence.

The Submitting Author accepts and understands that any supply made under these terms is made by BMJ to the Submitting Author unless you are acting as an employee on behalf of your employer or a postgraduate student of an affiliated institution which is paying any applicable article publishing charge ("APC") for Open Access articles. Where the Submitting Author wishes to make the Work available on an Open Access basis (and intends to pay the relevant APC), the terms of reuse of such Open Access shall be governed by a Creative Commons licence – details of these licences and which Creative Commons licence will apply to this Work are set out in our licence referred to above.

Other than as permitted in any relevant BMJ Author's Self Archiving Policies, I confirm this Work has not been accepted for publication elsewhere, is not being considered for publication elsewhere and does not duplicate material already published. I confirm all authors consent to publication of this Work and authorise the granting of this licence.

For peer review only

Hospital management of major stroke types in Chinese adults: a population-based study of 20,000 hospitalised stroke cases

Haiqiang Qin MD*[1], Iain Turnbull MRCP* [2], Yiping Chen DPhil* [2,3], Neil Wright PhD[2], Liping Liu MD[1], Pei Pei BSc[4], Wei Tang BSc[6], Shengping Xiang BSc[7], Yu Guo MSc[4] , Xingquan Zhao MD[1], Robert Clarke FRCP[2], Liming Li MPH[4,5], Yongjun Wang MD[1], Zhengming Chen DPhil[2] on behalf of the China Kadoorie Biobank Collaborative Group

Equal contribution

1. China National Clinical Research Centre for Neurological Diseases; Department of Neurology, Beijing Tiantan Hospital, Capital Medical University, Beijing, China
2. Clinical Trial Service Unit and Epidemiological Studies Unit (CTSU), Nuffield Department of Population Health, University of Oxford, Oxford, UK
3. Medical Research Council Population Health Research Unit (PHRU), Nuffield Department of Population Health, University of Oxford, Oxford, UK
4. Chinese Academy of Medical Sciences, Dong Cheng District, Beijing, China
5. Department of Epidemiology and Biostatistics, School of Public Health, Peking University Health Science Centre, Beijing, China
6. Emergency Department, Pengzhou Traditional Chinese Medical Hospital, Pengzhou, Sichuan, China
7. Pengzhou Tongyi Hospital, Pengzhou, Sichuan, China

Address for correspondence

Prof Robert Clarke
Clinical Trial Service Unit
NDPH
Big Data Institute Building
Old Road Campus
University of Oxford
E-mail: robert.clarke@ndph.ox.ac.uk

Dr. Yiping Chen
Clinical Trial Service Unit
NDPH
Big Data Institute Building
Old Road Campus
University of Oxford
E-mail: yiping.chen@ndph.ox.ac.uk

Word count: Abstract n=289; text n=3,107
3 Tables, 2 Figure 1 eTable and 4 eFigures

CKB Research Tracker no. 2018-0071

Key words: Ischaemic Stroke, hospital treatment, antiplatelet medication, blood pressure-lowering medication, lipid-lowering medication, traditional Chinese medicines

20 October 2021

For peer review only - http://bmjopen.bmj.com/site/about/guidelines.xhtml

Abstract

Objectives: To compare hospital treatments for major stroke types in Chinese adults by stroke pathological types, sex, age, calendar year, hospital tier, region and other factors.

Design: Cross-sectional analysis of medical records retrieved from 20,229 stroke cases in the China Kadoorie Biobank.

Setting: Ten diverse areas (5 urban, 5 rural) in China.

Participants: First-incident stroke cases who were recruited during an 11-year follow-up of 0.5M participants in the China Kadoorie Biobank.

Methods: Electronic copies of medical records of stroke cases were retrieved for clinical adjudication by local neurologists. Stroke cases were classified as ischaemic stroke (IS) (including lacunar infarction [LACI] and non-lacunar infarction [non-LACI]), intracerebral haemorrhage (ICH), subarachnoid haemorrhage (SAH) and unspecified stroke types.

Results: Among 20,299 first-ever stroke cases, 17,306 (85%) had IS, 7,123 had non-LACI, 6,690 had LACI, 3,493 had silent LACI, 2,623 (13%) had ICH, and 370 (2%) had SAH. Among IS cases, antiplatelet treatment was used by 64% (65% non-LACI, 66% LACI, 56% silent LACI), lipid-lowering by 50% (52% non-LACI, 53% LACI, 43% silent LACI), and blood pressure-lowering by ~42% of all IS types, with positive trends in the use of these treatments by calendar year and hospital tier. Among ICH cases, 53% used blood pressure-lowering and 10% used lipid-lowering treatments, respectively. In contrast, traditional Chinese medicines (TCM) were used by 59% of IS (50% non-LACI, 62% LACI, 74% silent LACI), 38% of ICH, and 30% of SAH cases, with positive trends by calendar year and by hospital tier.

Conclusions: Among IS cases, use of antiplatelet and lipid-lowering medications increased in recent years, but use of TCM still exceeded use of blood pressure-lowering treatment. In contrast, blood pressure-lowering treatment was widely used for ICH, but only half of all ICH cases used blood pressure-lowering treatment.

For peer review only - http://bmjopen.bmj.com/site/about/guidelines.xhtml

Strengths and limitations of this study

- The China Kadoorie Biobank (CKB) is a nationwide community-based prospective cohort study involving over 0.5 million men and women from 5 rural and 5 urban areas in China.
- Local neurologists classified 20,000 first-incident stroke cases that were reported over an 11-year follow-up into relevant pathological types.
- The data collection on use of medication was limited to in-hospital treatments only.
- The study was not representative of the Chinese population and data collection was limited to first-ever stroke cases.

For peer review only

For peer review only - http://bmjopen.bmj.com/site/about/guidelines.xhtml

Introduction

Stroke is a major cause of death and disability worldwide, with three-quarters (~6.3 million) of deaths each year occurring in developing countries including China.[1-3] In China, stroke is now the leading cause of death and disability, accounting for almost 2 million deaths in 2017.[3-5] While mortality rates from stroke have declined substantially over the last few decades in high-income countries, the annual costs for hospital treatment and rehabilitation of stroke were 40 billion Chinese Yuan (CNY) and approximately 6 billion US dollars (USD) in 2016, which were 10-fold higher than those for ischaemic heart disease (IHD).[3] The average per-patient cost of hospitalisation for stroke cases in China in 2018 was 14,291 Chinese Yuan (CNY) (9,409 CNY [1,309 USD] for ischaemic stroke and 19,149 CNY [2,772 USD] for haemorrhagic stroke).

In recent decades, evidence-based guidelines for treatment of different stroke pathological types have been developed, regularly updated, and widely disseminated in China. In contrast, whereas Traditional Chinese Medicines (TCM), for which no randomised evidence exists, continue to be extensively used for the treatment of hospitalised stroke cases.[6] Nevertheless, there is still substantial variation in access to and use of evidence-based treatments for stroke in different regions of China.[6] While such treatments have been extensively studied in Western countries,[7-9] little is known about the contemporary use of evidence-based medical and surgical treatments for hospitalised cases of stroke in China. Previous studies of hospital management of stroke in China have been constrained

For peer review only

For peer review only - http://bmjopen.bmj.com/site/about/guidelines.xhtml

by small sample sizes and involvement of only large teaching or tertiary hospitals, or restricted to studies in large urban areas only.[10-15]

We examined the use of medical and surgical treatments for first-ever incident stroke cases by pathological type, overall and by sex, age of onset, calendar year, hospital tier, region and other risk factors during an 11-year follow-up of 0.5 million adults in the China Kadoorie Biobank (CKB). [16,17]

Methods

Study population

Details of the study design and methods for recruitment and follow-up used in the CKB have been previously reported.[16,17] In brief, the 2004-8 baseline survey was conducted in 5 urban cities (Qingdao, Harbin, Liuzhou, Haikou, and Suzhou) and 5 rural counties (Henan, Hunan, Sichuan, Gansu, and Zhejiang) in China, selected to maximise regional differences in risk exposures and prevalence of major diseases. In each area, all permanent residents aged 35-74 years who were without disability were invited to participate. A total of 512,726 individuals (including some outside the target age range) agreed to participate in the study. The main analyses of the present report were restricted to adjudicated first-ever incident cases of stroke occurring prior to 31 December 2017 among 484,413 participants with no self-reported heart disease or stroke/transient ischaemic attack (TIA) at baseline (n=23,129) or stroke prior to their first adjudicated stroke (n=5,184).

For peer review only - http://bmjopen.bmj.com/site/about/guidelines.xhtml

Data on socio-demographic status, smoking and alcohol consumption, diet, physical activity and medical history were collected by trained health workers using laptop-based questionnaires at local study clinics. All participants also had clinical measurements recorded, including height, weight, waist and hip circumference, lung function, blood pressure and heart rate and a blood sample collected. Ethical approval for the China Kadoorie Biobank was obtained from the Oxford University Tropical Research Ethics Committee (OxTREC; [2005]; OxTREC Reference 025-04), and the Chinese Center for Disease Control and Prevention Ethical Review Committee (2004; Reference 005/2004). All participants provided written informed consent for participating and for accessing their medical records during follow-up by study investigators.

After the baseline survey, the vital status of participants was monitored regularly through Disease Surveillance Points (DSP) death registries, supplemented by annual checks with local residential records and active confirmation through local street committees or village administrators.[17] The occurrence of stroke among participants was identified by electronic linkage, using a unique personal identification number, to the national health insurance (HI) system that covered >97% of CKB participants, and to death and disease-specific (stroke, IHD, cancer and diabetes) registries accessible in the local study areas.

Selected medical records of hospitalised cases of first-ever reported stroke cases were retrieved and the diagnosis was verified by trained local CKB staff at each of the 261 different hospitals. The retrieved documents (e.g. initial covering page and discharge summary of the first admission record, with reports of brain imaging and use of pre-

For peer review only - http://bmjopen.bmj.com/site/about/guidelines.xhtml

specified medical treatments for stroke) were reviewed electronically by 26 accredited Chinese neurologists and stroke physicians from Tier III (top ranking) hospitals in China using bespoke IT systems. While indicators of medical treatments were pre-specified at three time periods (first 24 hours after admission, during hospital stay, and at discharge) in the medical notes, information on timing of such treatments was incomplete and consequently it was not possible to conduct separate analysis of use of drug treatment during each time period. Hence, recorded hospital treatment in any of the three periods was used in the present analysis.

Classification of stroke pathological types

All reported and adjudicated diagnoses of stroke pathological types were checked centrally by study staff and standardised by 10th Revision of the International Statistical Classification of Diseases and Related Health Problems (ICD-10) codes using bespoke software. The major stroke pathological types examined were ischaemic stroke (IS; ICD-10 I63, including lacunar infarction [LACI] and non-lacunar infarction [non-LACI]), intracerebral haemorrhage (ICH; I61), subarachnoid haemorrhage (SAH; I60), and unspecified stroke (I64). IS was defined as focal neurological dysfunction lasting for more than 24 hours with or without neuroimaging evidence of a cerebral infarct. LACI was classified by the evidence of an infarct <1.5 cm in diameter on CT (computed tomography) or MR (magnetic resonance) brain imaging with focal neurological deficits, including lacunar syndrome.[18] Silent lacunar infarct silent LACI) was characterised by imaging

For peer review only - http://bmjopen.bmj.com/site/about/guidelines.xhtml

evidence of a cerebral infarct ,1.5 cm in diameter in the absence of any focal neurological deficit. Non-LACI was defined as any IS type excluding LACI.

ICH was defined as a neurological dysfunction caused by haemorrhage into the brain parenchyma or the ventricular system, excluding those induced by injury, with or without neuroimaging evidence of haemorrhage. SAH was defined as a neurological dysfunction caused by a haemorrhage into the subarachnoid space, excluding those induced by injury, with or without relevant neuroimaging evidence. In addition, all silent LACI cases were reviewed centrally by two research clinicians with relevant training in neurology and included with other stroke cases in an “any stroke” endpoint.

Statistical analysis

The present study included a total of 20,299 first ever incident stroke cases with no prior history of a CVD event at baseline questionnaire or stroke event before the first adjudicated event. All cases were adjudicated online by senior neurologists in China and reviewed centrally by study clinicians in Oxford. The rates of incident stroke cases were standardised to the overall CKB population by sex, area, and age at diagnosis. Rates of use of medical and surgical treatments were estimated separately for any stroke and for stroke pathological types, by sex, and area (rural vs urban), separately. All analyses were conducted using R 3.5.0.

For peer review only - http://bmjopen.bmj.com/site/about/guidelines.xhtml

For peer review only

Patient and public involvement

Prior to recruitment in the CKB, local community leaders in China were consulted. The CKB study findings[19] are routinely reported in peer-review publications and any relevant public health messages disseminated[20] using local press, television and internet.

Results

Characteristics of cases with different stroke types

Among 484,413 participants included in the present analysis, the mean age (SD) at baseline was 51.5 (10.5) years, 59% were women, and 43% lived in urban areas (**Table 1**). During a median follow-up of 11 years (4.72 million person-years), a total of 61,902 incident stroke cases were reported, including 57,190 (92%) hospitalised cases. Overall, there were 20,299 first-ever hospitalised cases of primary stroke for which each diagnosis was confirmed by clinical adjudication, including 17,306 (85%) with IS (7,123 non-LACI, 6,690 LACI, and 3,493 silent LACI), 2,623 with ICH, and 370 with SAH, respectively. Overall, 93% of the reported stroke cases (92% for IS, 97% for ICH and 96% for SAH) had brain imaging.

Compared with those without stroke, individuals who experienced first-ever stroke during follow-up were older and more likely to be male, to reside in urban areas, and to have lower levels of education and household income at baseline (**Table 1**). Individuals with first-ever ICH were more likely than those with other stroke types to be men, to live in rural areas, and to have lower levels of full-time education (54.8% had no formal/primary

For peer review only

schooling: **Table 1**) and have low annual household income (30.6% had income less than 10,000 CNY: **Table 1**). Likewise, individuals with ICH had the highest proportion with self-reported hypertension (23.5%: **Table1**), and the highest mean levels of systolic blood pressure (SBP) (149 mmHg: **Table 1**). With the exception of LACI cases (who were more likely to be female and live in urban areas), individuals with non-LACI and LACI had broadly similar baseline characteristics (**Table 1**). In addition, the characteristics of individuals with LACI and silent LACI cases were comparable. However, compared with LACI, silent LACI cases had lower mean levels of SBP (133 mmHg vs 138 mmHg). The baseline characteristics of individuals with SAH were generally similar to those with ICH (**Table 1**). Interestingly, the overall prevalence of atrial fibrillation was very low, albeit higher in non-LACI than in LACI cases (0.3% vs 0.1%: **Table 2**).

Hospital treatment in IS cases

Overall, antiplatelet agents (aspirin or clopidogrel) were administered to 63.7% of all hospitalised IS cases, but use of antiplatelet agents was higher in cases with LACI (66.3%) and non-LACI (65.1%) than silent LACI cases (55.6%) (**Table 2**). Dual antiplatelet therapy was much less commonly used (non-LACI 8.3%, LACI 6.2%, and silent LACI 2.6%, respectively). Intravenous thrombolytic treatments (rtPA and urokinase) were used in 1.2% of non-LACI and 0.3% of LACI cases. Overall, 42% of all IS cases received blood pressure-lowering treatments, with similar proportions for different IS types (42.2%, 41.8%, and 40.8% for LACI, non-LACI, and silent LACI, respectively). Likewise, lipid-lowering treatments were used by only about half of all hospitalised cases of IS (52.6%, 51.5%, and

For peer review only - http://bmjopen.bmj.com/site/about/guidelines.xhtml

43.2% for LACI, non-LACI, and silent LACI, respectively). In contrast, 56% of IS cases (62.0%, 49.9%, and 73.6%, for LACI, non-LACI, and silent LACI, respectively) received TCM (**Table 2**).

For antiplatelet and lipid-lowering medications in addition to TCM, the rates of use for IS increased with age, while no such increasing trends with age were observed for use of blood-pressure lowering treatments **(Figure 1)**. All medical treatments for IS, with the exception of blood pressure-lowering therapy, increased by calendar year of follow-up between 2005 and 2017 **(Figure 2)**. While the use of blood pressure-lowering treatments did not differ significantly by hospital tiers, the use of TCM was higher in Tier III hospitals and increased by calendar year between 2005 to 2017 (**Figure 2**). Similarly, age-specific use of antiplatelet, blood pressure-lowering, and lipid-lowering agents and TCM among IS cases did not vary appreciably by sex or area of residence (**eFigures 1-6**), but use of TCM increased by hospital tier in both men and women and in rural and urban areas. Carotid endarterectomy and stenting were very rarely undertaken (**Table 2**).

Hospital management of ICH and SAH

Among participants hospitalised with ICH, 3.9% had surgical clot evacuation. A small proportion of cases with SAH underwent endovascular coiling (7.0%) or neurosurgical clipping of an aneurysm (5.7%). Approximately half of ICH (52.6%) or SAH (50.5%) cases received blood pressure-lowering medication, which did not vary significantly by sex or area (**eFigure 1**). Overall, the use of lipid-lowering agents was 10.4% for ICH whereas

For peer review only - http://bmjopen.bmj.com/site/about/guidelines.xhtml

approximately one-third of ICH (37.8%) and SAH (29.7%) cases received TCM while in hospital (**Table 3**).

Discussion

The present study, involving over 20,000 incident stroke cases, demonstrated that while there have been substantial improvements in access to and use of brain imaging for diagnosis of stroke types in hospitalised cases, the use of many evidence-based medical treatments, including antiplatelet, blood pressure-lowering, and lipid-lowering agents, remains low in China, even in top-rank hospitals. In contrast, the use of TCM has remained consistently high, especially in higher-ranking hospitals, highlighting the urgent need for better utilisation of evidence-based treatments, particularly in IS cases. Likewise, further trials are required to examine the efficacy and safety of such treatments in ICH cases. Importantly, further assessments of the use of TCM in the management of both IS and ICH are also indicated.[19]

Despite marked improvements in the hospital management of stroke in recent decades in China,[15] the use of evidence-based medications remains lower than in Western countries. Thrombolytic therapy (e.g. r-tPA), which was approved by the State Food and Drug Administration of China in 2001, was administered to <1% of potentially eligible cases in the present study, compared with 3.0-8.5% reported in the USA [20] and 1.6% reported by the Chinese National Stroke Registry (CNSR) in 2011.[21]

For peer review only - http://bmjopen.bmj.com/site/about/guidelines.xhtml

A recent American Heart Association registry study conducted during the same calendar period reported a higher use of antiplatelet agents in the USA vs China (83.9% vs 64%, respectively).[22] Since data on common adverse events including bleeding were not recorded in the present study, the possibility that the lower use of antiplatelet therapy was clinically indicated in this population cannot be entirely excluded. Likewise, the use of blood pressure-lowering and lipid-lowering medications was also higher in the USA compared with China (76.7% vs 41.7%, and 77.6% vs 50.3%, respectively).[22] Moreover, the use of drug treatments for secondary prevention of stroke in the CKB was significantly lower than those reported in the CNSR.[15] Lowering blood pressure is the most important treatment to improve outcomes of cases with SAH and ICH.[23,24] However, in the present study, only about half of all ICH and SAH cases (and only 42% of IS cases) received blood pressure-lowering treatment. One explanation for the discrepant findings on rates of use of treatment in stroke cases in the CKB and the CNSR may be that the former study was conducted in 10 diverse regions (5 rural regions and 5 urban) with 21% of hospitals involving lower-grade (Tier 1) hospitals, whereas recruitment of stroke cases in the latter study was restricted to those living in urban regions and those in higher-tier hospitals. For the same reason, the use of intravenous thrombolytic therapy was <1% in the CKB vs 1.5% in CNSR. Likewise, the prevalence of reported atrial fibrillation was lower than previously reported estimates from population studies in China, and only 7% of stroke cases had Holter monitoring investigations. In addition, medication data in the present study were obtained from retrieved hospital admission records rather than at later periods

For peer review only - http://bmjopen.bmj.com/site/about/guidelines.xhtml

of follow-up. Although in-hospital treatment data are reliable, intended use of medication following discharge from hospital may be underestimated.

While there is substantial underuse of evidence-based medical and surgical treatments in hospitalised stroke cases, TCM are widely used for all stroke types in China, particularly in Tier 3 hospitals, despite absent randomised evidence to support their efficacy and safety.[19,25] The overall rates of use of evidence-based medications were also lower among younger stroke cases consistent with previous reports.[26] The economic and social effects of stroke and its long-term sequelae in younger cases are considerable and these inequalities should be a priority for improvement. The suboptimal use of medical treatments may partly explain the high recurrence rates of IS recently reported in this study population.[27]

Rates of medical therapy did not vary appreciably among ischaemic stroke types (non-LACI, LACI, and silent LACI). However, relevant clinical guidelines address only the management of symptomatic stroke cases,[27,28] since there have been no randomised controlled trials including participants with silent LACI. The high prevalence of silent LACI – and its strong association with subsequent risks of symptomatic stroke, [29-31] cognitive decline and dementia, [32-34] psychiatric disorders,[35] and increased mortality,[36] highlight the need for new trials to assess the efficacy and safety of medical treatments in such cases.

The use of drug treatments has increased considerably in recent years, particularly after introduction of universal health coverage in 2009, reflecting the effects of health policy. A

For peer review only - http://bmjopen.bmj.com/site/about/guidelines.xhtml

recent report of the temporal trends in hospital admissions for stroke in China demonstrated that admissions for first stroke increased by 3-6% per year after 2009 following adjustment for age, other risk factors and incidence of other diseases.[37] These stroke admissions did not, however, vary appreciably between rural and urban areas or between hospital tiers, suggesting that low socio-economic status, which is more common in rural areas, is unlikely to explain the suboptimal use of evidence-based treatments for stroke in China.

Further studies on the adverse effects of medications (e.g., bleeding risks with antiplatelet agents or rhabdomyolysis in high-dose statin users) are also needed to improve our understanding of the reduced rates of use of evidence-based therapies for IS in Chinese populations. The consequences of their suboptimal use – and the relatively prevalent use of TCM – on short-term mortality and stroke recurrence following stroke also warrant further study.

The present study had several strengths, including the use of bespoke web-based adjudication systems to determine the diagnosis and clinical characteristics of over 20,000 reported stroke cases of all pathological types including silent LACI. Moreover, it included data from Tier I hospitals in rural areas that have not been included in previous studies. However, there were several limitations. Firstly, the CKB study was not a nationally representative study and, hence, the results cannot be representative of all areas in China. Secondly, it is possible that some selected clinical information relating to the acute phase of stroke (for example, administration of thrombolysis or aspirin treatment within 48 hours

For peer review only - http://bmjopen.bmj.com/site/about/guidelines.xhtml

of hospital admission) was recorded in the emergency room and omitted in the retrieved medical records. Not all hospitals recorded medications for intended treatment after discharge in the medical records, which may have led to underestimates of their actual use. Lastly, the present study did not collect records of side effects of treatments (e.g., bleeding associated with the use of antiplatelet agents). Consequently, the relatively low observed rates of use of medical treatment may reflect clinical discretion or caution. However, the possibility of missing clinical data was minimised by photographing all available relevant medical records and enlistment of specialist neurologists and stroke physicians from Tier III hospitals to adjudicate the diagnoses of stroke types.

Overall, this contemporary study of 20,000 hospitalised stroke cases demonstrated that use of all evidence-based medical treatments has increased substantially after the introduction of the nationwide universal healthcare system in China. Despite recent improvements in stroke management, there is still considerable underuse of many evidence-based treatments and widespread overuse of TCM for stroke in the absence of evidence on their efficacy and safety. Future studies should investigate the causes and consequences of variations in stroke care in clinical practice in China.[38] Moreover, further large randomised trials are needed to evaluate the efficacy and safety of established evidence-based treatments for silent LACI.

For peer review only - http://bmjopen.bmj.com/site/about/guidelines.xhtml

Disclosures: None of the authors have any disclosures that were relevant to this report.

Contributorship

HQ, IT, YC and RC designed the study. NW concluded the statistical analyses. HQ wrote the first draft and IT, YC and RC revised the report, tables and figures. PP, WT, SX, YG and XZ supervised the field work for data collection on drug treatment and ascertainment of outcomes. L Liu and YW provided critical advice on the analyses and interpretation and YG, LL and ZC supervised the overall conduct of the CKB study. All authors provided critical comments on the final report. RC and YC took responsibility for submission of the final version for publication.

Competing interests

None of the authors had any conflicts of interest in relation to this report.

Funding: The CKB baseline survey and the first re-survey were supported by the Kadoorie Charitable Foundation in Hong Kong. The long-term follow-up has been supported by Wellcome grants to Oxford University (212946/Z/18/Z, 202922/Z/16/Z, 104085/Z/14/Z, 088158/Z/09/Z) and grants from the National Key Research and Development Program of China (2016YFC0900500, 2016YFC0900501, 2016YFC0900504, 2016YFC1303904) and from the National Natural Silent LACIence Foundation of China (91843302). The UK Medical Research Council (MC_UU_00017/1,MC_UU_12026/2 MC_U137686851), Cancer Research UK (C16077/A29186; C500/A16896) and the British Heart Foundation (CH/1996001/9454), provide core funding to the Clinical Trial Service Unit and Epidemiological Studies Unit at Oxford University for the project.

For peer review only

Data availability statement

The China Kadoorie Biobank (CKB) is a global resource for the investigation of lifestyle, environmental, biochemical and genetic factors as determinants of common diseases. The CKB study group is committed to making the cohort data available to the scientific community in China, the UK and worldwide to advance knowledge about the causes, prevention and treatment of disease. Detailed information on data that are currently available for open access users and how to apply for these are provided on the study website (http://www.ckbiobank.org/site/Data+Access).

Researchers who are interested in obtaining the individual data from the China Kadoorie Biobank study that underlines this paper should contact ckbaccess@ndph.ox.ac.uk. A research proposal will be requested to ensure that analyses will be performed by bona fide researchers.

For peer review only - http://bmjopen.bmj.com/site/about/guidelines.xhtml

Figure Legends

Figure 1: Proportions of medical treatments in hospital by IS subtypes

Figure 2: Use of antiplatelet, antihypertensive, lipid−lowering and TCM treatments for ischaemic stroke by hospital tiers and calendar periods from 2005 to 2017

For peer review only

Table 1: Selected baseline characteristics of participants by types and subtypes of first adjudicated stroke

	IS			ICH	SAH	Any stroke	All CKB
	Non-LACI (n = 7,123)	LACI (n = 6,690)	Silent LACI (n = 3,493)	(n = 2,623)	(n = 370)	(n = 20,299)	(n = 484,413)
**Age, %**							
30 – 39	2.0	1.8	2.0	4.4	6.3	2.4	16.0
40 – 49	13.1	14.3	13.4	16.9	21.4	14.5	31.0
50 – 59	32.5	34.3	33.0	34.6	36.6	33.8	30.8
60 – 69	35.5	34.4	35.1	30.0	24.8	33.9	16.6
70 – 79	16.9	15.2	16.4	14.2	11.0	15.5	5.7
Mean (SD)	60.1 (9.6)	59.6 (9.5)	60.0 (9.6)	58.3 (10.0)	56.5 (10.2)	59.5 (9.7)	51.5 (10.5)
**Sex, %**							
Male	50.0	46.5	39.0	53.1	36.9	47.4	40.8
Female	50.0	52.3	59.8	46.9	60.3	52.6	59.2
**Area, %**							
Rural	58.0	40.6	39.0	63.3	51.6	49.2	56.8
Urban	42.0	59.4	61.0	36.7	48.4	50.8	43.2
**Highest education, %**							
No formal / primary school	54.4	52.4	53.2	54.8	46.4	53.4	50.7
Middle / high school	40.1	42.4	38.8	40.3	40.1	42.3	43.6
College / University	4.0	4.1	4.8	4.1	5.6	4.3	5.7
**Annual household income (RMB), %**							
<9,999	30.1	27.3	29.6	30.6	29.6	29.2	28.3
10,000-19,999	29.4	33.5	30.1	30.6	24.0	30.7	28.8
20,000-34,999	22.9	21.0	21.4	24.3	23.5	23.6	24.7
≥35000	16.0	17.0	15.9	13.7	15.1	16.5	18.1
**Current smoker, %**							

For peer review only - http://bmjopen.bmj.com/site/about/guidelines.xhtml

Males	69.4	67.1	55.1	62.5	52.1	66.5	62.0
Female	3.5	3.6	1.8	2.2	4.3	3.0	2.3
**Current alcohol drinker, %**							
Males	35.2	38.6	33.9	38.8	24.8	36.5	33.9
Female	2.5	1.4	2.0	3.7	1.3	2.5	2.1
**Regular dietary intake †, %**							
Meat	79.2	82.0	79.1	81.7	72.3	82.3	82.9
Fish	44.8	45.4	42.4	42.8	41.6	45.6	46.8
Dairy	16.8	18.6	19.5	16.2	19.1	18.3	19.7
Fruit	55.1	54.8	54.9	54.8	58.6	56.3	59.5
**Prevalent disease ‡, %**							
Diabetes	6.8	5.9	4.1	3.2	2.0	5.3	2.7
Hypertension	21.8	19.3	19.3	23.5	14.3	20.8	9.7
**Clinical measures, mean (SD)**							
SBP (mmHg)	140.3 (23.9)	138.4 (23.9)	132.8 (22.6)	149.0 (24.5)	124.7 (22.6)	142.1 (24.2)	130.4 (20.9)
BMI (kg/m^2)	24.3 (3.6)	24.0 (3.4)	23.3 (3.4)	24.0 (3.6)	21.7 (3.5)	24.4 (3.5)	23.6 (3.4)
RPG (mmol/L)	6.8 (3.5)	6.4 (3.3)	6.1 (2.9)	6.2 (2.9)	5.7 (3.2)	6.5 (3.2)	6.0 (2.3)
**Hospital tier, %**							
Tier 1 (162 hospitals) *	2.7	4.5	2.6	2.6	3.4	3.2	
Tier 2 (53 hospitals)	4.2	6.2	3.7	4.7	3.1	4.7	
Tier 3 (46 hospitals)	91.6	88.1	90.6	91.9	85.7	92.0	

Means and percentages are directly standardised to age, sex and study area structure of the CKB study population after excluding 23,129 participants with prior CHD or stroke/TIA and 5,184 participants with a reported stroke prior to first adjudicated stroke, as appropriate.

† 1-3 days per week or more often

‡ Self-reported at baseline survey

* includes 126 community health centres

Abbreviations: LACI: lacunar infarction, SD: standard deviation, SBP: systolic blood pressure, BMI: body mass index, RPG: random plasma glucose

For peer review only - http://bmjopen.bmj.com/site/about/guidelines.xhtml

Table 2: Hospital managements for subtypes of IS

Treatment, n (%)	Non-LACI (n = 7,123)		LACI (n = 6,690)		Silent LACI (n = 3,492)		All IS (n = 17,305)		P
**Any antiplatelet agent**	4,638	(65.1)	4,434	(66.3)	1,943	(55.6)	11,015	(63.7)	<0.001
Aspirin	3,829	(53.8)	3,722	(55.6)	1,761	(50.4)	9,312	(53.8)	<0.001
Clopidogrel	456	(6.4)	431	(6.4)	132	(3.8)	1,019	(5.9)	<0.001
Dual antiplatelet therapy	589	(8.3)	413	(6.2)	90	(2.6)	1,092	(6.3)	<0.001
**Anticoagulant agent**	689	(9.7)	569	(8.5)	146	(4.2)	1,404	(8.1)	<0.001
**Any antihypertensive agent**	2,976	(41.8)	2,820	(42.2)	1,426	(40.8)	7,222	(41.7)	0.439
1 Antihypertensive agent	2,217	(31.1)	2,137	(31.9)	1,046	(30.0)	5,400	(31.2)	0.119
2 Antihypertensive agent	746	(10.5)	668	(10.0)	354	(10.1)	1,768	(10.2)	0.629
≥3 Antihypertensive agent	170	(2.4)	156	(2.3)	110	(3.2)	436	(2.5)	0.028
**Any lipid-lowering agent**	3,670	(51.5)	3,522	(52.6)	1,508	(43.2)	8,700	(50.3)	<0.001
Statin	3,601	(50.6)	3,441	(51.4)	1,461	(41.8)	8,503	(49.1)	<0.001
Other lipid lowering agent	133	(1.9)	130	(1.9)	63	(1.8)	326	(1.9)	0.879
**Thrombolytic Therapy**									<0.001
r-tPA	25	(0.4)	5	(0.1)	0	(0.0)	30	(0.2)	<0.001
Urokinase	60	(0.8)	15	(0.2)	0	(0.0)	75	(0.4)	<0.001
**Defibrase**	192	(2.7)	179	(2.7)	43	(1.2)	414	(2.4)	<0.001
**Any TCM**	3,545	(49.8)	4,151	(62.0)	2,571	(73.6)	10,267	(59.3)	0.095
**Atrial fibrillation**	24	(0.3)	8	(0.1)	4	(0.1)	36	(0.2)	<0.001

For peer review only

For peer review only - http://bmjopen.bmj.com/site/about/guidelines.xhtml

Table 3: Hospital management for ICH and SAH

Hospital management, n (%)	ICH (n = 2,623)		SAH (n = 370)	
**Brain Imaging (CT or MRI)**		(97.0)		(96.0)
**Any antihypertensive agent**	1,379	(52.6)	187	(50.5)
1 Antihypertensive agent	899	(34.3)	146	(39.5)
2 Antihypertensive agent	436	(16.6)	42	(11.4)
≥3 Antihypertensive agent	151	(5.8)	8	(2.2)
**Any lipid-lowering agent**	272	(10.4)	12	(3.2)
Statin	266	(10.1)	12	(3.2)
Other lipid lowering agent	8	(0.3)	0	(0.0)
**Any TCM**	992	(37.8)	110	(29.7)
**Surgery**	230	(8.8)	47	(12.7)
Clot evacuation	103	(3.9)	3	(0.8)
Coiling	0	(0.0)	26	(7.0)
Aneurysm clipping surgery	0	(0.0)	21	(5.7)

For peer review only

For peer review only - http://bmjopen.bmj.com/site/about/guidelines.xhtml

References

1. Feigin VL, Roth GA, Naghavi M, et al. Global burden of stroke and risk factors in 188 countries, during 1990-2013: a systematic analysis for the Global Burden of Disease Study 2013. Lancet Neurol 2016; **15**(9): 913-24.
2. Feigin VL, Krishnamurthi RV, Parmar P, et al. Update on the Global Burden of Ischemic and Hemorrhagic Stroke in 1990-2013: The GBD 2013 Study. Neuroepidemiology 2015; **45**(3): 161-76.
3. Chen WW, Gao RL, Liu LS, et al Summary of China Cardiovascular Diseases Report. *Chinese Circulation Journal;* **32** : 521-530
4. Institute for Health Metrics and Evaluation (IHME). GBD Compare. Seattle, WA: IHME,University of Washington, 2015. Available from http://vizhub.healthdata.org/gbd-compare. (accessed February 18, 2020).
5. China National Health Commission. China Health and Family Planning Statistical Yearbook 2019. *Peking Union Medical College Press* 2019.
6. Wu S, Wu B, Liu M, et al. Stroke in China: advances and challenges in epidemiology, prevention, and management. Lancet Neurol 2019; **18**(4): 394-405.
7. Bangalore S, Schwamm L, Smith EE, et al. Secondary Prevention after Ischemic Stroke or Transient Ischemic Attack. The American journal of medicine 2014.
8. Fonarow GC, Reeves MJ, Smith EE, et al. Characteristics, performance measures, and in-hospital outcomes of the first one million stroke and transient ischemic attack admissions in get with the guidelines-stroke. Circ Cardiovasc Qual Outcomes 2010; **3**(3): 291-302.
9. Feigin VL, Lawes CM, Bennett DA, Anderson CS. Stroke epidemiology: a review of population-based studies of incidence, prevalence, and case-fatality in the late 20th century. Lancet neurology 2003; **2**(1): 43-53.
10. Wang Y, Li Z, Zhao X, et al. Effect of a Multifaceted Quality Improvement Intervention on Hospital Personnel Adherence to Performance Measures in Patients With Acute Ischemic Stroke in China: A Randomized Clinical Trial. JAMA 2018; **320**(3): 245-54.
11. Heeley E, Anderson CS, Huang Y, et al. Role of health insurance in averting economic hardship in families after acute stroke in China. Stroke; a journal of cerebral circulation 2009; **40**(6): 2149-56.
12. Ding D, Lu CZ, Fu JH, Hong Z. Predictors of vascular events after ischemic stroke: the China ischemic stroke registry study. Neuroepidemiology 2010; **34**(2): 110-6.
13. Liu X, Xu G, Wu W, Zhang R, Yin Q, Zhu W. Subtypes and one-year survival of first-ever stroke in Chinese patients: The Nanjing Stroke Registry. Cerebrovasc Dis 2006; **22**(2-3): 130-6.
14. He Q, Wu C, Luo H, et al. Trends in In-Hospital Mortality among Patients with Stroke in China. PloS one 2014; **9**(3): e92763.
15. Li Z, Wang C, Zhao X, et al. Substantial Progress Yet Significant Opportunity for Improvement in Stroke Care in China. Stroke 2016; **47**(11): 2843-9.
16. Chen Z, Lee L, Chen J, et al. Cohort profile: the Kadoorie Study of Chronic Disease in China (KSCDC). Int J Epidemiol 2005; **34**(6): 1243-9.

For peer review only - http://bmjopen.bmj.com/site/about/guidelines.xhtml

17. Chen Z, Chen J, Collins R, et al. China Kadoorie Biobank of 0.5 million people: survey methods, baseline characteristics and long-term follow-up. Int J Epidemiol 2011; **40**(6): 1652-66.
18. Asdaghi N, Jeerakathil T, Hameed B, et al. Oxfordshire community stroke project classification poorly differentiates small cortical and subcortical infarcts. Stroke 2011; **42**(8): 2143-8.
19. Chen Z, Sandercock P, Xie JX, Peto R, Collins R, Liu LS. Hospital management of acute ischemic stroke in China. Journal of stroke and cerebrovascular diseases : the official journal of National Stroke Association 1997; **6**(5): 361-7.
20. Reeves MJ, Arora S, Broderick JP, et al. Acute stroke care in the US: results from 4 pilot prototypes of the Paul Coverdell National Acute Stroke Registry. Stroke; a journal of cerebral circulation 2005; **36**(6): 1232-40.
21. Wang Y, Liao X, Zhao X, et al. Using recombinant tissue plasminogen activator to treat acute ischemic stroke in China: analysis of the results from the Chinese National Stroke Registry (CNSR). Stroke 2011; **42**(6): 1658-64.
22. Bushnell CD, Olson DM, Zhao X, et al. Secondary preventive medication persistence and adherence 1 year after stroke. Neurology 2011; **77**(12): 1182-90.
23. Connolly ES, Jr., Rabinstein AA, Carhuapoma JR, et al. Guidelines for the management of aneurysmal subarachnoid hemorrhage: a guideline for healthcare professionals from the American Heart Association/american Stroke Association. Stroke 2012; **43**(6): 1711-37.
24. Hemphill JC, 3rd, Greenberg SM, Anderson CS, et al. Guidelines for the Management of Spontaneous Intracerebral Hemorrhage: A Guideline for Healthcare Professionals From the American Heart Association/American Stroke Association. Stroke 2015; **46**(7): 2032-60.
25. Wei JW, Huang Y, Wang JG, et al. Current management of intracerebral hemorrhage in China: a national, multi-centre, hospital register study. BMC Neurol 2011; **11**: 16.
26. Sappok T, Faulstich A, Stuckert E, Kruck H, Marx P, Koennecke HC. Compliance with secondary prevention of ischemic stroke: a prospective evaluation. Stroke 2001; **32**(8): 1884-9.
27. Kernan WN, Ovbiagele B, Black HR, et al. Guidelines for the prevention of stroke in patients with stroke and transient ischemic attack: a guideline for healthcare professionals from the American Heart Association/American Stroke Association. Stroke 2014; **45**(7): 2160-236.
28. Smith EE, Saposnik G, Biessels GJ, et al. Prevention of Stroke in Patients With Silent Cerebrovascular Disease: A Silent LACIentific Statement for Healthcare Professionals From the American Heart Association/American Stroke Association. Stroke 2017; **48**(2): e44-e71.
29. Fanning JP, Wong AA, Fraser JF. The epidemiology of silent brain infarction: a systematic review of population-based cohorts. BMC Med. 2014;12:119.
30. Gupta A, Giambrone AE, Gialdini G, Finn C, Delgado D, Gutierrez J, et al. Silent Brain Infarction and Risk of Future Stroke: A Systematic Review and Meta-Analysis. Stroke. 2016;47(3):719-25.
31. Vermeer SE, Hollander M, van Dijk EJ, Hofman A, Koudstaal PJ, Breteler MM. Silent brain infarcts and white matter lesions increase stroke risk in the general population: the Rotterdam Scan Study. Stroke. 2003;34(5):1126-9.
32. Vermeer SE, Prins ND, den Heijer T, Hofman A, Koudstaal PJ, Breteler MM. Silent brain infarcts and the risk of dementia and cognitive decline. N Engl J Med. 2003;348(13):1215-22.

For peer review only

33. Bos D, Wolters FJ, Darweesh SKL, Vernooij MW, de Wolf F, Ikram MA, et al. Cerebral small vessel disease and the risk of dementia: A systematic review and meta-analysis of population-based evidence. Alzheimers Dement. 2018;14(11):1482-92.
34. Smith EE, O'Donnell M, Dagenais G, Lear SA, Wielgosz A, Sharma M, et al. Early cerebral small vessel disease and brain volume, cognition, and gait. Ann Neurol. 2015;77(2):251-61.
35. van Agtmaal MJM, Houben A, Pouwer F, Stehouwer CDA, Schram MT. Association of Microvascular Dysfunction With Late-Life Depression: A Systematic Review and Meta-analysis. JAMA Psychiatry. 2017;74(7):729-39.
36. Bokura H, Kobayashi S, Yamaguchi S, Iijima K, Nagai A, Toyoda G, et al. Silent brain infarction and subcortical white matter lesions increase the risk of stroke and mortality: a prospective cohort study. J Stroke Cerebrovasc Dis. 2006;15(2):57-63.
37. Chen Y, Wright N, Guo Y, et al Mortality and recurrent vascular events after first incident stroke: a 9-year community-based study of 0·5 million Chinese adults. *Lancet Global Health* 2020, 8(4):e580-90
38. Levy M, Chen Y, Clarke R et al Socioeconomic differences in health-care use and outcomes for stroke and ischemic heart disease in China during 2009–16: a prospective cohort study of 0·5 million adults*; Lancet Global Health* 2020; 8(4): e591-e602

For peer review only - http://bmjopen.bmj.com/site/about/guidelines.xhtml

Figure 1: Proportions of medical treatments in hospital by IS subtype

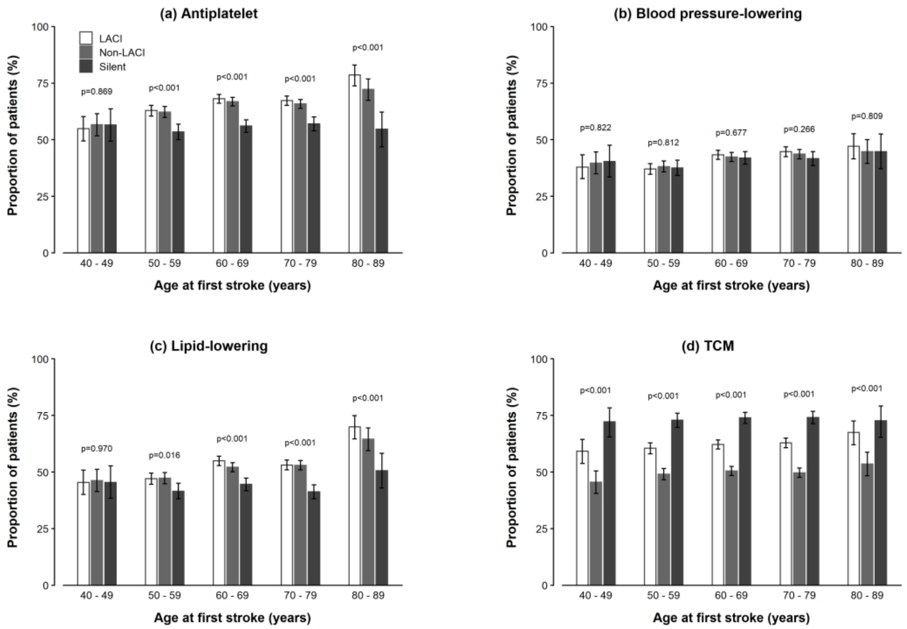

Figure 1: Proportions of medical treatments in hospital by IS type

296x210mm (320 x 320 DPI)

Figure 2: Antiplatelet, antihypertensive, lipid-lowering and TCM treatments for ischaemic stroke by hospital tiers and calendar periods from 2005 to 2017

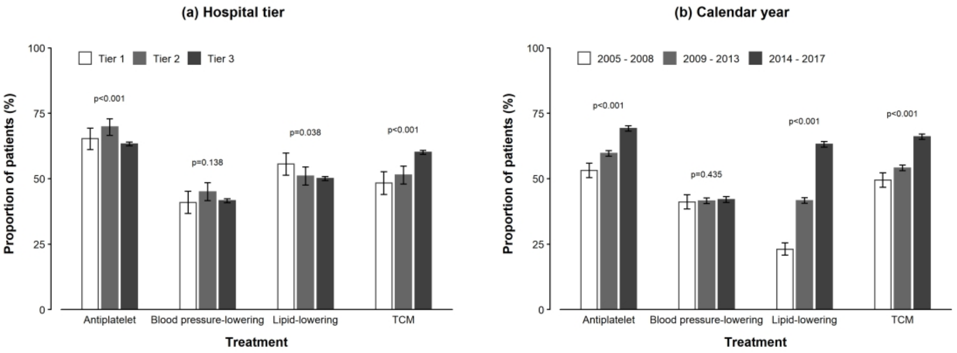

Figure 2: Antiplatelet, antihypertensive, lipid-lowering and TCM treatments for ischaemic stroke by hospital tiers and calendar periods from 2005 to 2017

296x210mm (320 x 320 DPI)

Supplementary Material for: "Hospital management of major stroke types in Chinese adults: a population-based study of 20,000 hospitalised stroke cases"

Members of the China Kadoorie Biobank collaborative group

eFigure 1: Proportions of use of antiplatelet treatments in hospital by IS subtype, sex and area

eFigure 2: Proportions of use of blood pressure-lowering treatments in hospital by IS subtype, sex and area

eFigure 3: Proportions of use of lipid-lowering treatments in hospital by IS subtype, sex and area

eFigure 4: Proportions of use of TCM in hospital by IS subtype, sex and area

eFigure 5: Use of antiplatelet, antihypertensive, lipid-lowering and TCM treatments for ischaemic stroke by hospital tiers, sex and area

eFigure 6: Use of antiplatelet, antihypertensive, lipid-lowering and TCM treatments for ischaemic stroke by calendar periods from 2005 to 2017

For peer review only

Members of the China Kadoorie Biobank collaborative group:

International Steering Committee: Junshi Chen, Zhengming Chen (PI), Robert Clarke, Rory Collins, Yu Guo, Liming Li (PI), Chen Wang, Jun Lv, Richard Peto, Robin Walters.

International Co-ordinating Centre, Oxford: Daniel Avery, Fiona Bragg, Derrick Bennett, Ruth Boxall, Ka Hung Chan, Yumei Chang, Yiping Chen, Zhengming Chen, Johnathan Clarke; Robert Clarke, Huaidong Du, Zammy Fairhurst-Hunter, Hannah Fry, Wei Gan, Simon Gilbert, Alex Hacker, Parisa Hariri, Mike Hill, Michael Holmes, Pek Kei Im, Andri Iona, Maria Kakkoura, Christiana Kartsonaki, Rene Kerosi, Kuang Lin, Mohsen Mazidi, Iona Millwood, Qunhua Nie, Alfred Pozarickij, Paul Ryder, Sam Sansome, Dan Schmidt, Paul Sherliker, Rajani Sohoni, Becky Stevens, Iain Turnbull, Robin Walters, Lin Wang, Neil Wright, Ling Yang, Xiaoming Yang, Pang Yao.

National Co-ordinating Centre, Beijing: Yu Guo, Xiao Han, Can Hou, Chun Li, Chao Liu, Jun Lv, Pei Pei, Canqing Yu.

10 Regional Co-ordinating Centres: Guangxi Provincial CDC: Naying Chen, Duo Liu, Zhenzhu Tang. Liuzhou CDC: Ningyu Chen, Qilian Jiang, Jian Lan, Mingqiang Li, Yun Liu, Fanwen Meng, Jinhuai Meng, Rong Pan, Yulu Qin, Ping Wang, Sisi Wang, Liuping Wei, Liyuan Zhou. **Gansu** Provincial CDC: Caixia Dong, Pengfei Ge, Xiaolan Ren. Maiji CDC: Zhongxiao Li, Enke Mao, Tao Wang, Hui Zhang, Xi Zhang. **Hainan** Provincial CDC: Jinyan Chen, Ximin Hu, Xiaohuan Wang. Meilan CDC: Zhendong Guo, Huimei Li, Yilei Li, Min Weng, Shukuan Wu. **Heilongjiang** Provincial CDC: Shichun Yan, Mingyuan Zou, Xue Zhou. Nangang CDC: Ziyan Guo, Quan Kang, Yanjie Li, Bo Yu, Qinai Xu. **Henan** Provincial CDC: Liang Chang, Lei Fan, Shixian Feng, Ding Zhang, Gang Zhou. Huixian CDC: Yulian Gao, Tianyou He, Pan He, Chen Hu, Huarong Sun, Xukui Zhang. **Hunan** Provincial CDC: Biyun Chen, Zhongxi Fu, Yuelong Huang, Huilin Liu, Qiaohua Xu, Li Yin. Liuyang CDC: Huajun Long, Xin Xu, Hao Zhang, Libo Zhang. **Jiangsu** Provincial CDC: Jian Su, Ran Tao, Ming Wu, Jie Yang, Jinyi Zhou, Yonglin Zhou. Suzhou CDC: Yihe Hu, Yujie Hua, Jianrong Jin Fang Liu, Jingchao Liu, Yan Lu, Liangcai Ma, Aiyu Tang, Jun Zhang. **Qingdao** Qingdao CDC: Liang Cheng, Ranran Du, Ruqin Gao, Feifei Li, Shanpeng Li, Yongmei Liu, Feng Ning, Zengchang Pang, Xiaohui Sun, Xiaocao Tian, Shaojie Wang, Yaoming Zhai, Hua Zhang, Licang CDC: Wei Hou, Silu Lv, Junzheng Wang. **Sichuan** Provincial CDC: Xiaofang Chen, Xianping Wu, Ningmei Zhang, Weiwei Zhou. Pengzhou CDC: Xiaofang Chen, Jianguo Li, Jiaqiu Liu, Guojin Luo, Qiang Sun, Xunfu Zhong. **Zhejiang** Provincial CDC: Weiwei Gong, Ruying Hu, Hao Wang,Meng Wan, Min Yu. Tongxiang CDC: Lingli Chen, Qijun Gu, Dongxia Pan, Chunmei Wang, Kaixu Xie, Xiaoyi Zhang.

Event Adjudication Clinicians: Beijing Tiantan Hospital,Capital Medical University Shuya Li, Haiqiang Qin, Yongjun Wang, **Peking University People's Hospital** Qiling Chen, Jihua Wang, **The 1st Affiliated Hospital of Harbin Medical University** Xiaojia Sun, Lei Wang, Xun Wang, Liming Zhang, Shanshan Zhou, **The 2nd Affiliated Hospital of Harbin Medical University** Hongyuan Chen, Li Chen, Haiyan Gou, Weizhi Wang, Yanmei Zhu, Yulan Zhu, **The 2nd Hospital of Hebei Medical University** Ning Zhang, **Huashan Hospital** Xin Cheng, Qiang Dong, Yi Dong, Kun Fang, Yiting Mao, **Jinling Hospital** Yu An, Peiling Chen, Yinghua Chen, Zhihong Liu, Lihua Zhang **The People's Hospital of Liaoning Province** Xiaohong Chen, Naixin Jv, Xiaojiu Li, Liyang Liu, Yun Lu, Xiaona Xing, **Qingdao Fuwai Cardiovascular Hospital** Shihao You, **Shengjing Hospital of China Medical University** Xiaoli Cheng, Chaojun Gua, Jinping Jiang, Jingyi Liu, Shumei Ma, **Shenyang Military General Hospital** Xuefeng Yang, **The First People's Hospital of Shenyang** Xiaomo Du, Jian Xu, Xuecheng Yang, Xiaodi Zhao, **West China Hospital, Sichuan University** Zilong Hao, Ming Liu, Deren Wang, **The Second Affiliated Hospital of Suzhou University** Xiaoting Li, **Suzhou Kowloon Hospital Shanghai Jiao Tong University School of Medicine** Lili Hui, Zhanling Liao, Feng Liu, **Qingdao Fuwai Cardiovascular Hospital** Chunning Feng, Dejiang Ji, Fengxia Qu, Wenwen Yuan, **The First Affiliated Hospital of Zhengzhou University** Xin Fu, **Zhongshan Hospital,** Jing Ding, Peng Du, Lirong Jin, Yueshi Mao, Xin Wang.

For peer review only

eFigure 1: Proportions of use of antiplatelet treatments in hospital by IS subtype, sex and area

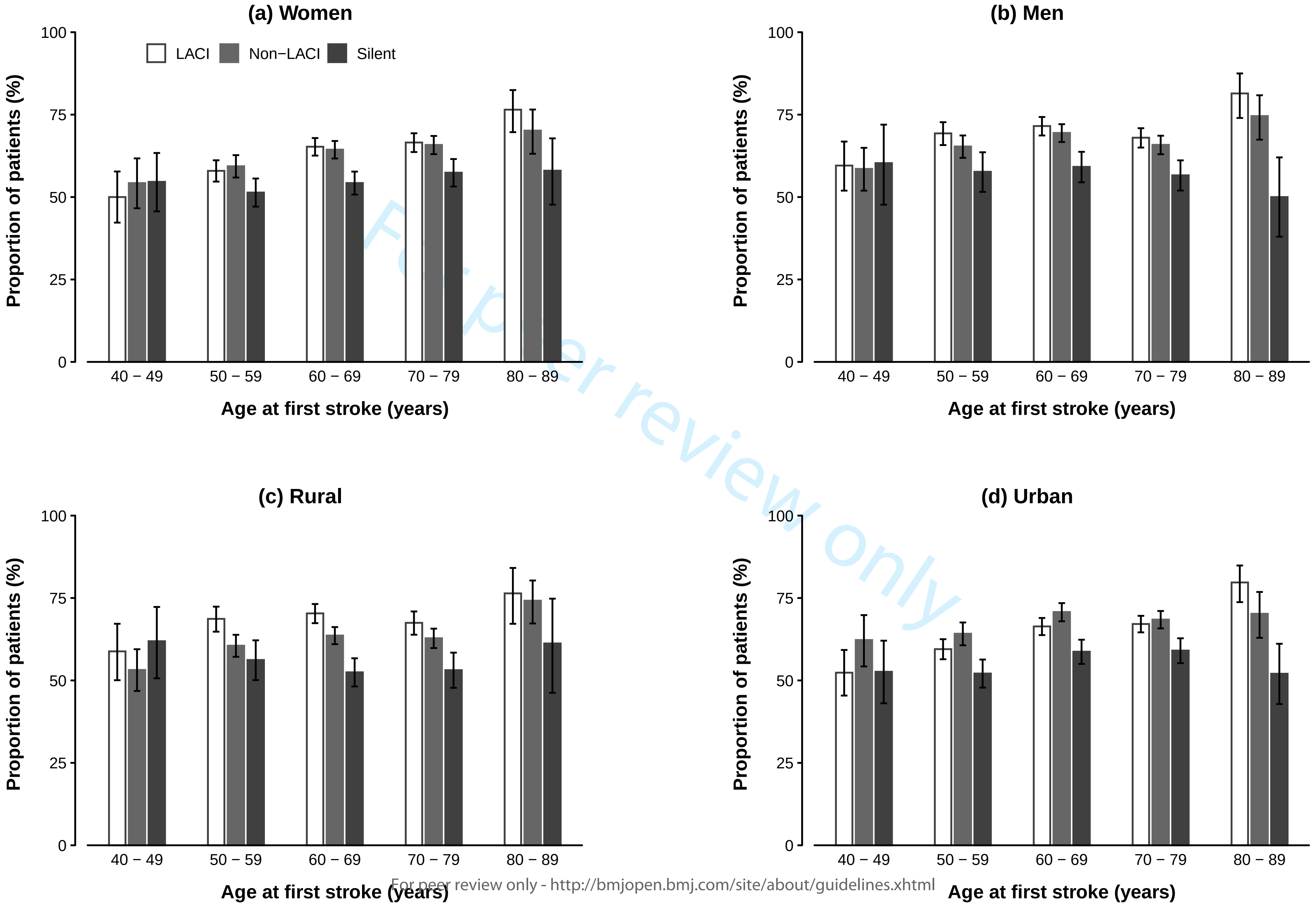

For peer review only

eFigure 2: Proportions of use of blood pressure−lowering treatments in hospital by IS subtype, sex and area

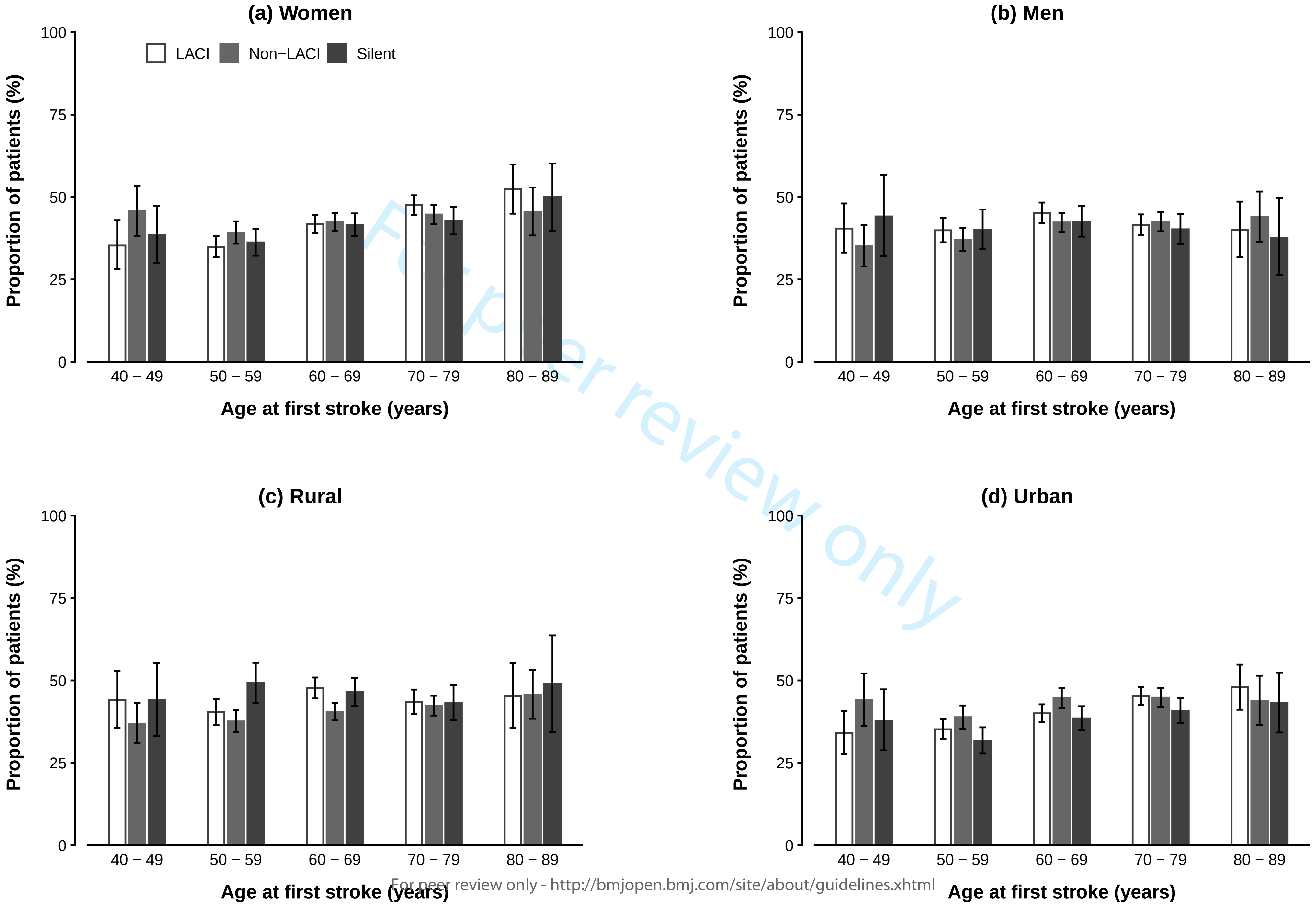

For peer review only

eFigure 3: Proportions of use of lipid−lowering treatments in hospital by IS subtype, sex and area

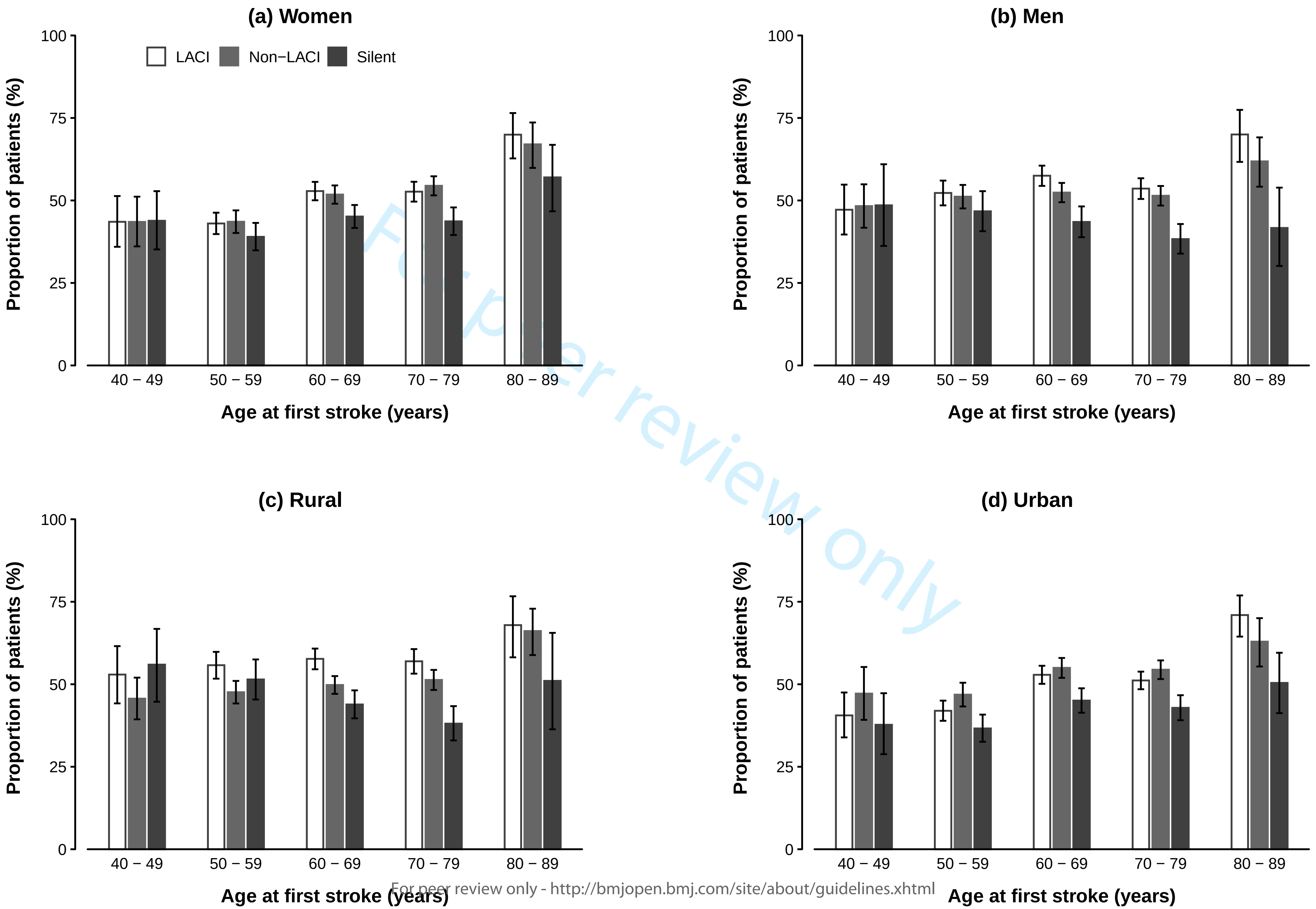

For peer review only

eFigure 4: Proportions of use of TCM in hospital by IS subtype, sex and area

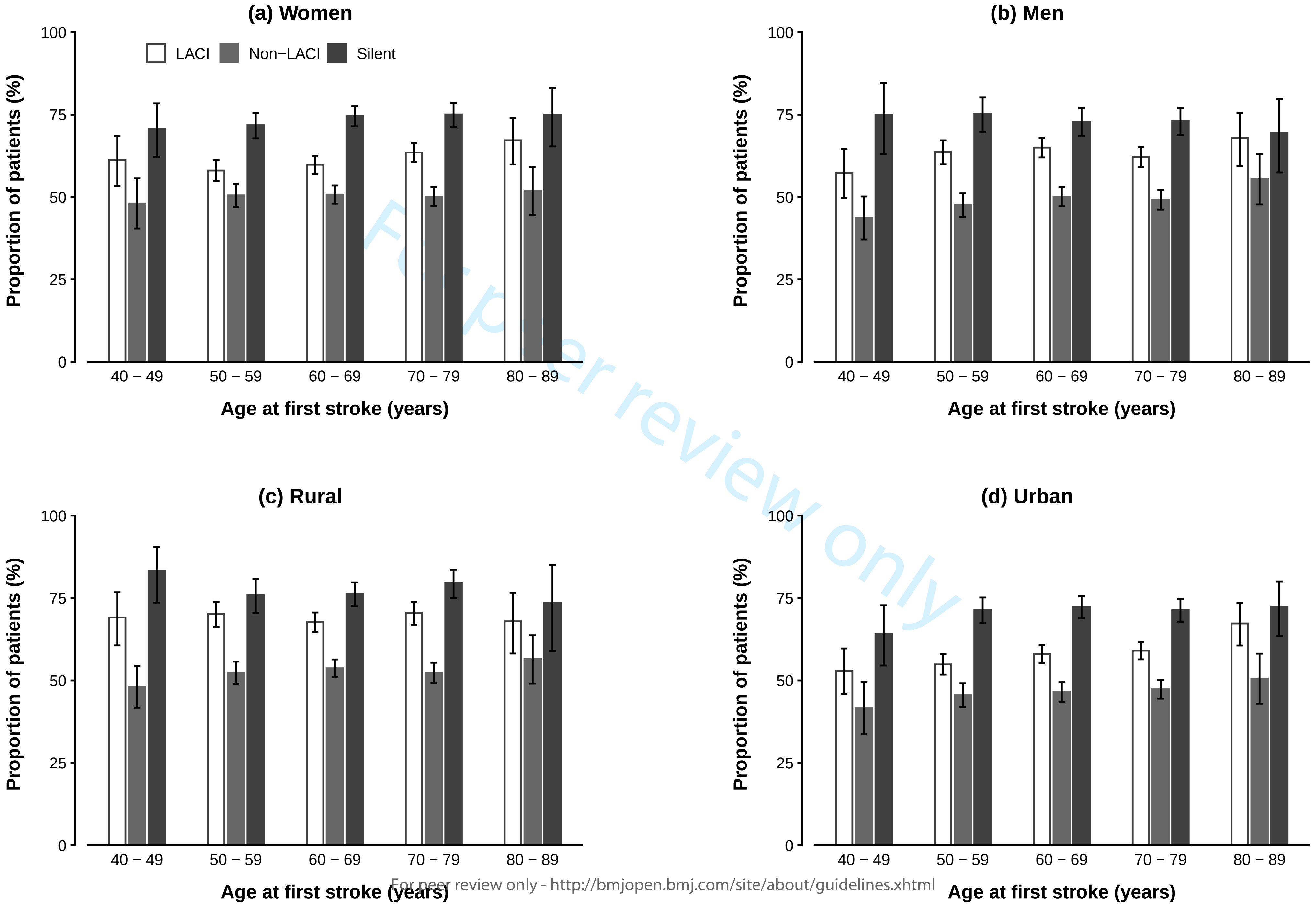

For peer review only

eFigure 5: Use of antiplatelet, antihypertensive, lipid−lowering and TCM treatments for ischaemic stroke by hospital tiers, sex and area

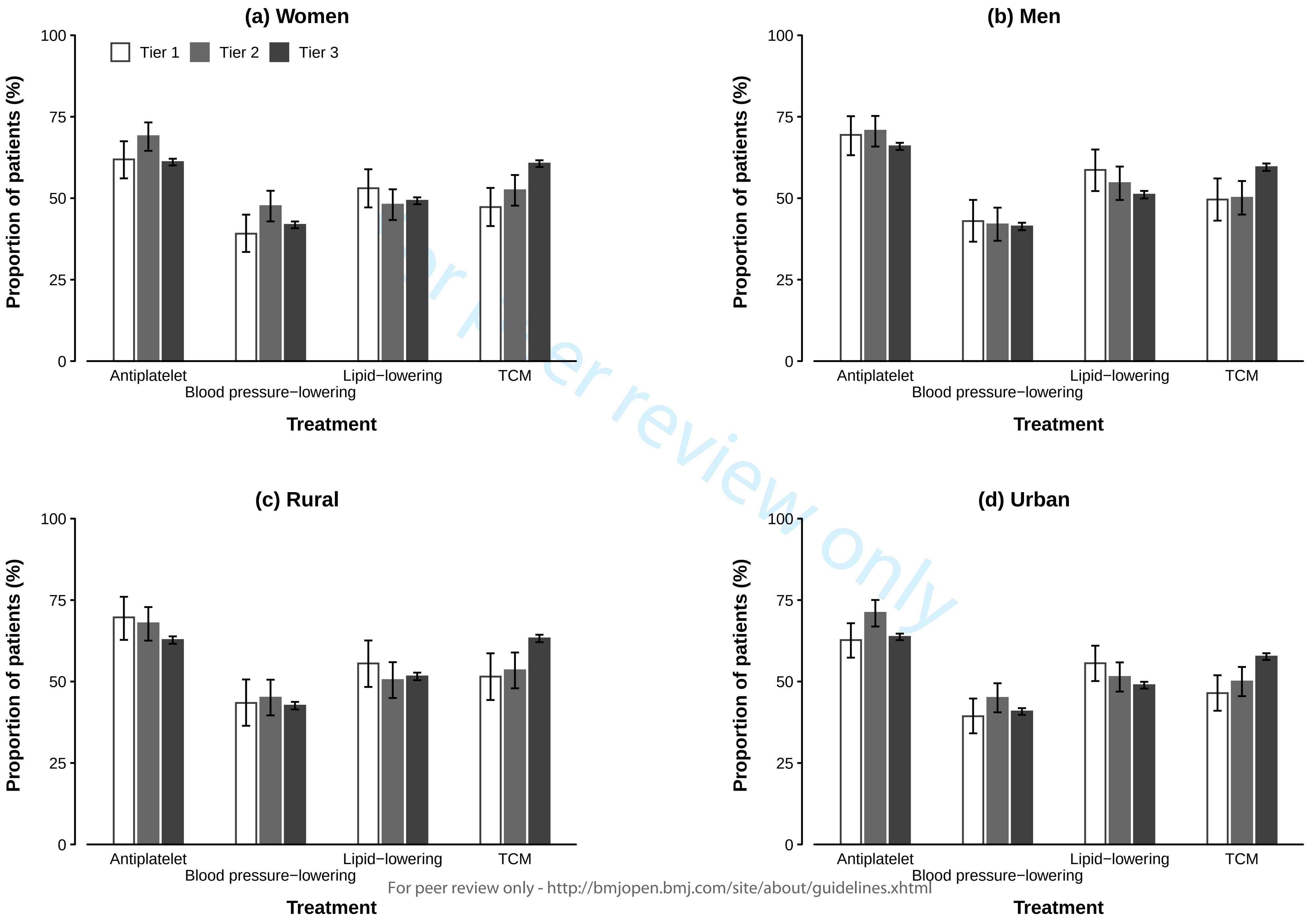

eFigure 6: Use of antiplatelet, antihypertensive, lipid−lowering and TCM treatments for ischaemic stroke by calendar periods from 2005 to 2017, sex and area

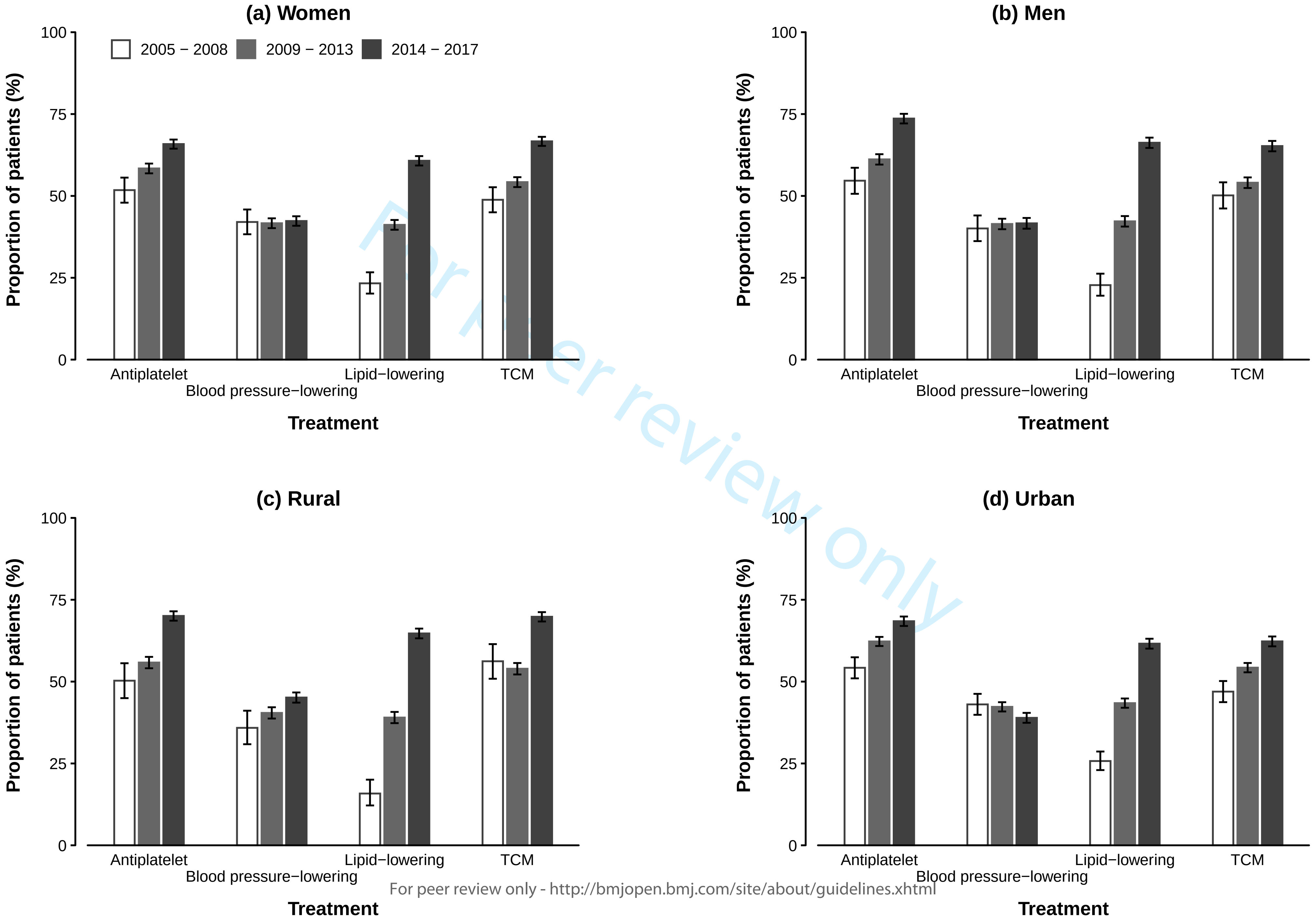

For peer review only

STROBE Statement—checklist of items that should be included in reports of observational studies

	Item No	Recommendation	Page No
**Title and abstract**	1	(*a*) Indicate the study's design with a commonly used term in the title or the abstract	1
		(*b*) Provide in the abstract an informative and balanced summary of what was done and what was found	2
**Introduction**			
Background/rationale	2	Explain the scientific background and rationale for the investigation being reported	3
Objectives	3	State specific objectives, including any prespecified hypotheses	3
**Methods**			
Study design	4	Present key elements of study design early in the paper	4
Setting	5	Describe the setting, locations, and relevant dates, including periods of recruitment, exposure, follow-up, and data collection	4
Participants	6	(*a*) *Cohort study*—Give the eligibility criteria, and the sources and methods of selection of participants. Describe methods of follow-up<br>*Case-control study*—Give the eligibility criteria, and the sources and methods of case ascertainment and control selection. Give the rationale for the choice of cases and controls<br>*Cross-sectional study*—Give the eligibility criteria, and the sources and methods of selection of participants	4-5
		(*b*) *Cohort study*—For matched studies, give matching criteria and number of exposed and unexposed<br>*Case-control study*—For matched studies, give matching criteria and the number of controls per case	
Variables	7	Clearly define all outcomes, exposures, predictors, potential confounders, and effect modifiers. Give diagnostic criteria, if applicable	5-6
Data sources/ measurement	8*	For each variable of interest, give sources of data and details of methods of assessment (measurement). Describe comparability of assessment methods if there is more than one group	*5-6*
Bias	9	Describe any efforts to address potential sources of bias	5
Study size	10	Explain how the study size was arrived at	4
Quantitative variables	11	Explain how quantitative variables were handled in the analyses. If applicable, describe which groupings were chosen and why	
Statistical methods	12	(*a*) Describe all statistical methods, including those used to control for confounding	7
		(*b*) Describe any methods used to examine subgroups and interactions	7
		(*c*) Explain how missing data were addressed	7
		(*d*) *Cohort study*—If applicable, explain how loss to follow-up was addressed<br>*Case-control study*—If applicable, explain how matching of cases and controls was addressed<br>*Cross-sectional study*—If applicable, describe analytical methods taking account of sampling strategy	7
		(*e*) Describe any sensitivity analyses	

Continued on next page

For peer review only - http://bmjopen.bmj.com/site/about/guidelines.xhtml

Results

Participants	13*	(a) Report numbers of individuals at each stage of study—eg numbers potentially eligible, examined for eligibility, confirmed eligible, included in the study, completing follow-up, and analysed	7
		(b) Give reasons for non-participation at each stage	
		(c) Consider use of a flow diagram	
Descriptive data	14*	(a) Give characteristics of study participants (eg demographic, clinical, social) and information on exposures and potential confounders	7-8
		(b) Indicate number of participants with missing data for each variable of interest	
		(c) *Cohort study*—Summarise follow-up time (eg, average and total amount)	
Outcome data	15*	*Cohort study*—Report numbers of outcome events or summary measures over time	*8-10*
		Case-control study—Report numbers in each exposure category, or summary measures of exposure	
		Cross-sectional study—Report numbers of outcome events or summary measures	
Main results	16	(*a*) Give unadjusted estimates and, if applicable, confounder-adjusted estimates and their precision (eg, 95% confidence interval). Make clear which confounders were adjusted for and why they were included	8-10
		(*b*) Report category boundaries when continuous variables were categorized	
		(*c*) If relevant, consider translating estimates of relative risk into absolute risk for a meaningful time period	
Other analyses	17	Report other analyses done—eg analyses of subgroups and interactions, and sensitivity analyses	

Discussion

Key results	18	Summarise key results with reference to study objectives	10-14
Limitations	19	Discuss limitations of the study, taking into account sources of potential bias or imprecision. Discuss both direction and magnitude of any potential bias	13-14
Interpretation	20	Give a cautious overall interpretation of results considering objectives, limitations, multiplicity of analyses, results from similar studies, and other relevant evidence	14
Generalisability	21	Discuss the generalisability (external validity) of the study results	14

Other information

Funding	22	Give the source of funding and the role of the funders for the present study and, if applicable, for the original study on which the present article is based	Author statement

*Give information separately for cases and controls in case-control studies and, if applicable, for exposed and unexposed groups in cohort and cross-sectional studies.

Note: An Explanation and Elaboration article discusses each checklist item and gives methodological background and published examples of transparent reporting. The STROBE checklist is best used in conjunction with this article (freely available on the Web sites of PLoS Medicine at http://www.plosmedicine.org/, Annals of Internal Medicine at http://www.annals.org/, and Epidemiology at http://www.epidem.com/). Information on the STROBE Initiative is available at www.strobe-statement.org.

For peer review only
